# Dynamic transitions of initiator binding coordinate the replication of the two chromosomes in *Vibrio cholerae*

**DOI:** 10.1038/s41467-024-55598-9

**Published:** 2025-01-08

**Authors:** Théophile Niault, Ariel Talavera, Eric Le Cam, Sonia Baconnais, Ole Skovgaard, Florian Fournes, Léa Wagner, Hedvig Tamman, Andrew Thompson, Dannele Echemendia-Blanco, Noa Guzzi, Abel Garcia-Pino, Didier Mazel, Marie-Eve Val

**Affiliations:** 1 Institut Pasteur, Université Paris Cité, CNRS UMR3525, Unité Plasticité du Génome Bactérien, Département Génomes et Génétique, Paris, France; 2 Sorbonne Université, Collège Doctoral, Paris, France; 3 Cellular and Molecular Microbiology, Faculté des Sciences, Université libre de Bruxelles (ULB), Boulevard du Triomphe, Brussels, Belgium; 4 Genome Integrity and Cancer UMR 9019 CNRS, Université Paris Saclay, Gustave Roussy, Villejuif, France; 5 Department of Science and Environment, Systems and Models, Roskilde University, Roskilde, Denmark; 6 SOLEIL Synchrotron, Saint-Aubin - BP48, Gif sur Yvette, France; 7 WEL Research Institute, Wavre, Belgium

**Keywords:** Origin firing, Bacterial genetics

## Abstract

The replication of the two chromosomes in the pathogenic bacterium *Vibrio cholerae* is coordinated by the binding of initiator protein RctB to a checkpoint sequence, *crtS*. Replication of *crtS* on the primary chromosome (Chr1) triggers replication of the secondary chromosome (Chr2), but the details are poorly understood. Here, we analyze RctB binding patterns in the *V. cholerae* genome across various cell cycle stages. We find that RctB primarily binds to sites inhibiting replication initiation at the Chr2 origin (*ori2*). This inhibitory effect is counteracted when *crtS* is replicated on Chr1, causing a shift in RctB binding to sites that activate replication at *ori2*. Structural analyzes indicate the formation of diverse oligomeric states of RctB, coupled to the allosteric effect of DNA, which determine *ori2* accessibility. We propose a synchronization model where, upon replication, *crtS* locally destabilizes the RctB inhibition complex, releasing the Chr2 replication origin.

## Abstract (web summary)

The replication of the two chromosomes in the bacterium *Vibrio cholerae* is coordinated through the binding of initiator protein RctB to a checkpoint sequence, *crtS*. Here, Niault et al. study RctB binding dynamics across the *V. cholerae* cell cycle to shed light into the underlying mechanisms.

## Introduction

Replication initiation is a crucial step in the bacterial life cycle, subject to complex regulatory controls to adapt to fluctuating growth conditions^1,2^. This complexity increases further for bacteria with multipartite genomes, which require an additional layer of control to replicate multiple chromosomes simultaneously^3^. Although substantial evidence across a variety of bacterial species supports the idea that the replication of multiple chromosomes must be coordinated^4–9^, *Vibrio cholerae* is the only species where a specific mechanism synchronizing the replication of two chromosomes has been identified^10–12^. In this pathogen, a replication checkpoint sequence, called *crtS*, ensures the synchronous replication and termination of the primary (Chr1) and secondary chromosome (Chr2) within a single replication cycle^12^. The objective of the present study is to elucidate the molecular mechanism by which *crtS* orchestrates this synchronization process.

*V. cholerae* Chr2 originates from an iteron-type plasmid, evident from its replication origin (*ori2*) and initiator (RctB)^13^. RctB is divided into four structural regions (I–IV)^14^ (Supplementary Fig. 1a). Its two central domains (II, III) are structurally similar to the Rep iteron plasmid initiators domains (WH1, WH2) while domains (I, IV) are unique to RctB^14^. Domains, I, II and III, interact with DNA via Helix-Turn-Helix (HTH) motifs and are essential for *ori2* replication^14^. The C-terminal domain (IV) is essential for down-regulating Chr2 initiation and has been shown in vitro to play a crucial role in self-interaction, promoting cooperative DNA binding and oligomerization^15,16^.

RctB recognizes three types of double-stranded DNA sites: iterons, 29/39 m and *crtS*. To promote replication initiation at *ori2*, RctB binds to an array of six regularly spaced iterons (six 12-bp repeats), which prompts DNA unwinding at an adjacent AT-rich region known as the DNA Unwinding Element (DUE) (Supplementary Fig. 1b)^17^. At this location, RctB engages with single strand of the open DUE on six regularly spaced direct repeats (5’-ATCA)^18^. A nucleoprotein complex, composed of RctB, IHF and DnaA, enables the recruitment of the replicative helicase DnaB, similar to what occurs in iteron plasmids^19^. Each iteron site contains a GATC motif which is specifically recognized and methylated by the Dam methylase. Iteron sites must be methylated on both DNA strand for RctB to bind^20^. RctB also recognizes 29-mer or 39-mer (29/39 m) sites, named after their size, which differ from iterons in both sequence and function (Supplementary Fig. 1b). The 29/39 m sites play a regulatory role in inhibiting *ori2* initiation^21^. Two 39 m sites and one 29 m site flanking the minimal *ori2* (containing the six iteron array and the DUE) participate in RctB-mediated iterons handcuffing (pairing via initiator bridges)^21,22^. The 29 m site is also part of the *rctB* gene promoter and contributes to *rctB* self-repression^22^. Additionally, RctB binds to a unique 62 bp sequence on Chr1, named *crtS*^11,12,23^. This sequence is crucial for synchronizing the replication of Chr1 and Chr2^12^. The replication of *crtS* triggers the initiation of Chr2, and its position relative to Chr1 origin (*ori1*) sets the replication timing for Chr2^12^. If *crtS* is deleted, *V. cholerae* undergoes lethal loss of Chr2 due to under-initiation at *ori2*^12^. Although the action of *crtS* has been indirectly linked to the inhibitory function of the 29/39 m sites^16^, the exact molecular dynamics guiding the coordination of Chr1 and Chr2 replication via *crtS* throughout the cell cycle remain largely undefined.

In this study, we conducted a comprehensive, genome-wide analysis of RctB binding, unveiling key elements of the *crtS*-triggered mechanism for Chr2 replication initiation at various stages of the *V. cholerae* cell cycle. We identified novel RctB binding sites and established a dynamic pattern of RctB binding throughout the cell cycle. Our findings reveal that RctB predominantly binds to the 29/39 m sites within *ori2*, effectively preventing the initiation of Chr2 replication for a large portion of the cell cycle. Using transmission electron microscopy (TEM), we observed that RctB forms large nucleoprotein complexes at *ori2*, linking the 29/39 m sites together. We show that RctB Domain IV can mediate alternative dimerization interfaces, suggesting their role in the assembly of oligomeric bridging structures when bound to 29/39 m sites. Following *crtS* replication, we observed a marked shift in RctB binding preferences at *ori2*, transitioning towards the iterons and the DUE, thereby enabling Chr2 replication initiation. Using an integrative approach combining ChIP-seq, live cell microscopy and structural studies, we propose a model of the firing of Chr2 replication in which one *crtS* duplication event directly triggers the activation of one *ori2* by destabilizing an inhibition complex.

## Results

### Genome-wide binding profile of RctB in *V. cholerae*

**Fig. 1:**
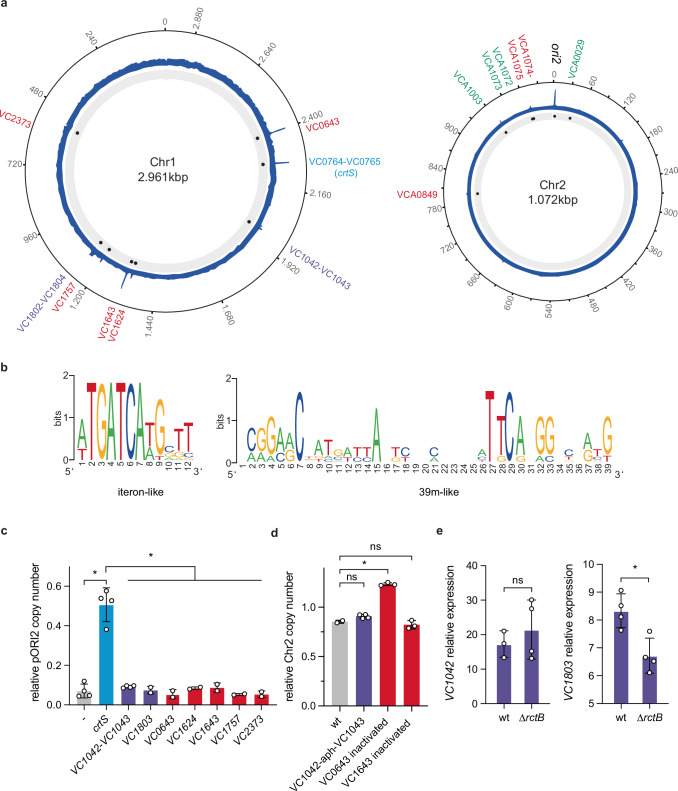
Chr2 initiator RctB binds preferentially to inhibitory sites on *ori2.* **a** RctB ChIP signal plotted on the circular map of *V. cholerae* N16961 using shinyCircos^63^ with reference sequences of Chr1 (CP028827.1) and Chr2 (CP028828.1)^64^, genes annotation (VCNNNN and VCANNNN) is based on the Heidelberg et al. genome^65^. The RctB ChIP signal is shown in blue. Significant enrichment peaks are marked with a blue dot with the proximal genes indicated in red (39m-like motifs), green (iteron-like motifs), light blue (*crtS*) and purple (no similarity to known motifs). **b** ChIP peak pattern analysis using the MEME suite^24^. Two significantly enriched motifs correspond to known RctB binding sequences: iterons (left) and 39 m (right). **c** pORI2 copy number (pORI2/oriC) in *E. coli* strains containing different RctB binding regions inserted into the *lacZ* locus. Each *E. coli* strain carries a plasmid (pORI2) that replicates using the *V. cholerae* Chr2 origin (*ori2*). The ratio of pORI2 to the chromosomal origin (*oriC*) serves as a measure of *ori2* replication activity. By inserting specific RctB binding regions into the genome, we can assess how these regions influence the replication of pORI2. Measurements were conducted with at least two biological replicates. Individual data points are shown, and the bars represent mean values. **d** Chr2 copy number relative to Chr1 (ori2/ori1) in non-replicating V. cholerae. For VC0643 and VC1643 contained in CDS RctB binding sites have been inactivated without perturbing the amino acid sequence, for VC1042-1043 the intergenic region containing RctB has been deleted. Measurements were conducted with at least two biological replicates. Individual data points are shown, and the bars represent mean values. **e** Expression of VC1042 and VC1803 genes relative to the housekeeping gene *gyrA* in the N16961 wild-type strain (WT), and MCH2 (∆*rctB*) *V. cholerae* mutant carrying fused chromosomes^46^. Measurements were performed with at least three biological replicates. Individual data points are shown, with bars representing the mean ± standard deviation. Statistical significance was assessed using a unpaired t-test, with non-significant differences indicated as “ns” and significant differences denoted by an asterisk (*). For VC1042, WT vs. ΔrctB: ns, *p* = 0.469. For VC1803, WT vs. ΔrctB: significant (*), *p* = 0.0103.

We conducted a ChIP-seq of RctB in *V. cholerae* to investigate its genome-wide binding profile within an exponentially growing population. In comparison to a previous ChIP-on-chip study^11^, we found that RctB binds to more regions across both chromosomes. These include 8 regions on Chr1 and 6 regions on Chr2, one of which encompasses *ori2* (Fig. 1a, Supplementary Data 1). Most of these regions contain either iterons-like sites with a 5’-TGATCA palindrome or 29/39m-like sites with a 5’-TTACGG motif, as revealed by MEME analysis^24^ (Fig. 1b). We also identified three regions that do not share any sequence similarity with these motifs (Supplementary Data 1), including *crtS* in the intergenic region between VC0764 and VC0765 on Chr1 (Figs. 1a, Supplementary Fig. 2)^23^. Given that our ChIP-seq was executed on non-synchronized, exponentially growing bacterial cultures, the heights of the peaks offer valuable insights into the frequency with which RctB binds at various sites throughout the cell cycle. To assess the impact of the newly discovered RctB binding sites on Chr2 replication, we used a plasmid-based reporter system, pORI2, that exclusively relies on *ori2* for replication^23^. Each RctB binding regions (spanning 250 bp on each side and centered around the peak) was inserted into the *lacZ* gene of *Escherichia coli* chromosome, and pORI2 copy number was measured using quantitative digital PCR (dPCR). Our results show that *crtS* stands out as the only strong enhancer of *ori2* initiation (Fig. 1c). Mutations in three of the most prominent binding sites (VC0643, VC01643, and VC1042-VC1043) had minimal effects on Chr2 replication in *V. cholerae*; although the 39m-like site within VC0643 did display a weak, yet statistically significant, negative effect on Chr2 copy number (Fig. 1d). Given RctB’s ability to repress its own transcription by binding to its promoter^22^, we explored whether RctB could repress other genes. We monitored the transcription levels of VC1042 and VC1803 genes in *V. cholerae*, both of which have an RctB binding site within their promoters. RT-dPCR analyzes revealed minimal to no differences in the expression of these genes, regardless of RctB presence (Fig. 1e). Although the RctB binding in the VC1803 promoter exhibited a weak, yet statistically significant, positive regulatory effect on VC1803 transcription. VC1803 is a component of Vibrio pathogenicity island 2 (VPI-2) and contains a Cro/C1 repressor-like HTH domain profile commonly found in transcriptional repressors of temperate bacteriophages, suggesting potential gene regulation by RctB within the pathogenicity island. In conclusion, while *crtS* plays a pivotal role in regulating Chr2 replication, the functional relevance of the newly discovered RctB binding sites on Chr1 remains uncertain. RctB appears to regulate its own transcription exclusively, in contrast to the initiator DnaA, which acts more broadly as a general transcription factor^25^.

### RctB binds predominantly to 29/39 m inhibitory sites within *ori2*

**Fig. 2:**
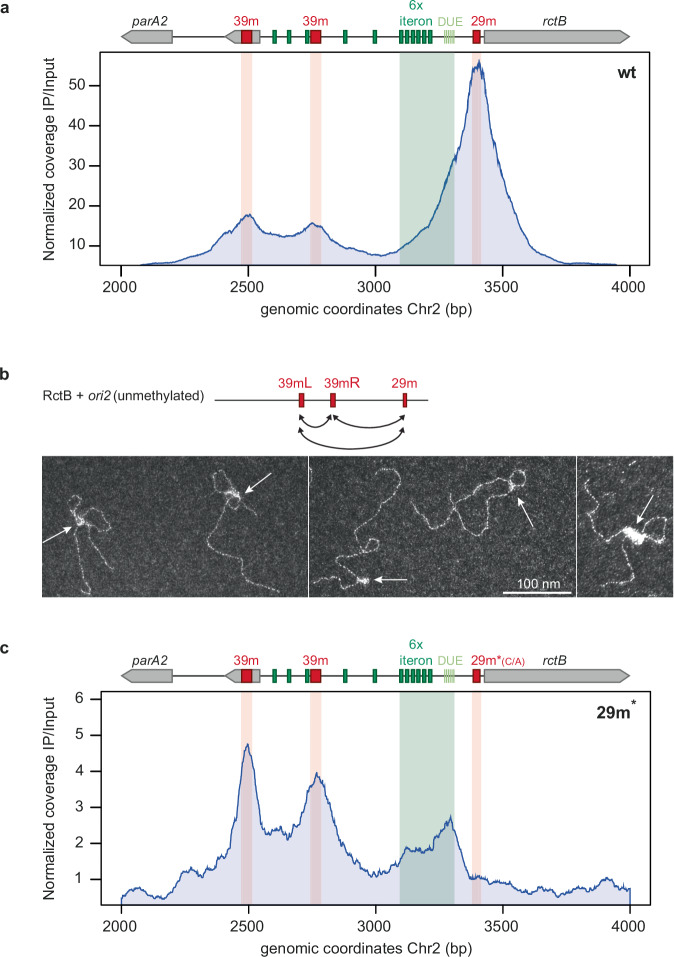
RctB predominantly binds to 29/39 m sites, forming a nucleoprotein complex that introduces DNA loops into *ori2* preventing binding to iterons and DUE. **a** ChIP-seq of RctB in *V. cholerae* N16961 focused on *ori2*. The y-axis represents the normalized ChIP signal of RctB (IP/input coverage), the x-axis displays a 2Kbp window centered on *ori2*, with genomic coordinates of CP028828.1. The genetic context and RctB binding sites are depicted above the plot (Supplementary Fig. 1b for more details on ori2). **b** TEM observations of nucleoprotein complexes formed by the binding of RctB to unmethylated DNA containing *ori2* (including the 39 mL, 39mR, and 29 m sites). RctB binding to DNA forms loops within *ori2*, bridging the inhibitory sites (indicated by white arrows). Potential interactions leading to loop formation are represented by double-headed black arrows. Observations were performed using at least three independent DNA and RctB incubation experiments. **c** ChIP-seq of RctB on *ori2* in a mutant having a point mutation in the 29 m site (TTGGAACTATAGTGATATTA[**C** > **A**]GGTAAGTG) preventing RctB binding at this site. Same legend as 2a.

We observed that the RctB ChIP signal was about 10-fold higher at the *ori2* region than at other chromosomal loci (Supplementary Fig. 2). Notably, RctB showed a strong preference for binding to the 29/39 m inhibitory sites (Fig. 2a). To further explore this binding pattern, we performed ChIP-seq on RctB during the stationary phase, when cells are not replicating (Supplementary Fig. 3). Interestingly, the RctB binding pattern at *ori2* in non-replicating cells closely mirrored what we observed during the replicating state of exponential growth. This predominant binding at the inhibitory sites suggests that Chr2 replication initiation is largely impeded throughout most of the cell cycle, reflecting similar behavior in both replicating and non-replicating cultures. Given that exponential-phase cultures consist of a mixture of cells at different stages of the replication cycle (asynchronous culture), we hypothesized that RctB interactions with the iterons and DUE are likely very transient, occurring only during short intervals of the replication cycle. This transient nature may explain why these interactions are not readily detected in a mixed population of replicating cells.

In both conditions, the signal at the 29 m site was approximately 3-fold higher than that at the two 39 m sites. We hypothesized that this discrepancy might arise from the ParB2 protein, essential for Chr2 segregation, competing with RctB for binding to both 39 m sites, as previously reported^26^. The ChIP-seq results of ParB2 on *ori2* are consistent with competitive binding of ParB2 and RctB at both 39 m sites and not at the 29 m site (Supplementary Fig. 4).

To further investigate the binding requirements of RctB at *ori2*, we performed a ChIP-seq analysis of RctB in a strain deleted for *dam* methylase (*Δdam*#4). RctB cannot bind to iterons in this mutant because they are not methylated, precluding initiation at *ori2*^20,27^. The *∆dam*#4 mutant is viable due to the integration of Chr2 into Chr1, enabling its replication from *ori1* as the DnaA initiator does not require Dam methylation^27^ (Supplementary Fig. 5a). In the ∆*dam#4* mutant, the RctB binding pattern at *ori2* was identical to that of the wild-type strain (Supplementary Fig. 5b). This result demonstrates that RctB molecules can form a complex at *ori2* strictly through their interaction with 29/39 m sites, without any engagement with iterons. It also confirms that RctB binding to *crtS* is independent of Dam as shown in ref. ^23^.

### RctB binding to 29/39 m sites generates DNA loops in *ori2* and prevents its binding to iterons

To directly visualize the complex formed by RctB bound to 29/39 m on *ori2*, we used positive-staining transmission electron microscopy (TEM)^28^. An unmethylated DNA fragment containing *ori2* was chosen to allow RctB to bind exclusively to the 29/39 m sites. Our observations revealed that RctB formed large nucleoprotein complexes bridging the 29 m and 39 m sites together, causing looping within the origin (Figs. 2b, white arrows and Supplementary Fig. 6, Supplementary Fig. 7). Such loops in *ori2* could create a steric hindrance preventing the initiation of replication by interfering with RctB binding to the iteron array and the proper unwinding of the DNA strands. To test this hypothesis, we performed a ChIP-seq of RctB in a mutant with the C_21_ > A mutation at the 29 m site (29 m*_(C/A)_) as this mutation has already been shown to reduce RctB binding in vivo^16^. The binding pattern of RctB at *ori2* was drastically altered (Fig. 2c). RctB still bound to both 39 m sites but not to the mutated 29 m. Additionally, we observed a clear binding of RctB to the array of six iterons and the adjacent DUE. Given that initiation of *ori2* replication requires RctB interaction with both the iteron array and the DUE^18^, our observations suggest that preventing RctB binding to 29 m effectively alleviates the constraint on *ori2* initiation.

### RctB^IV^ harbors two dimerization interfaces

**Fig. 3:**
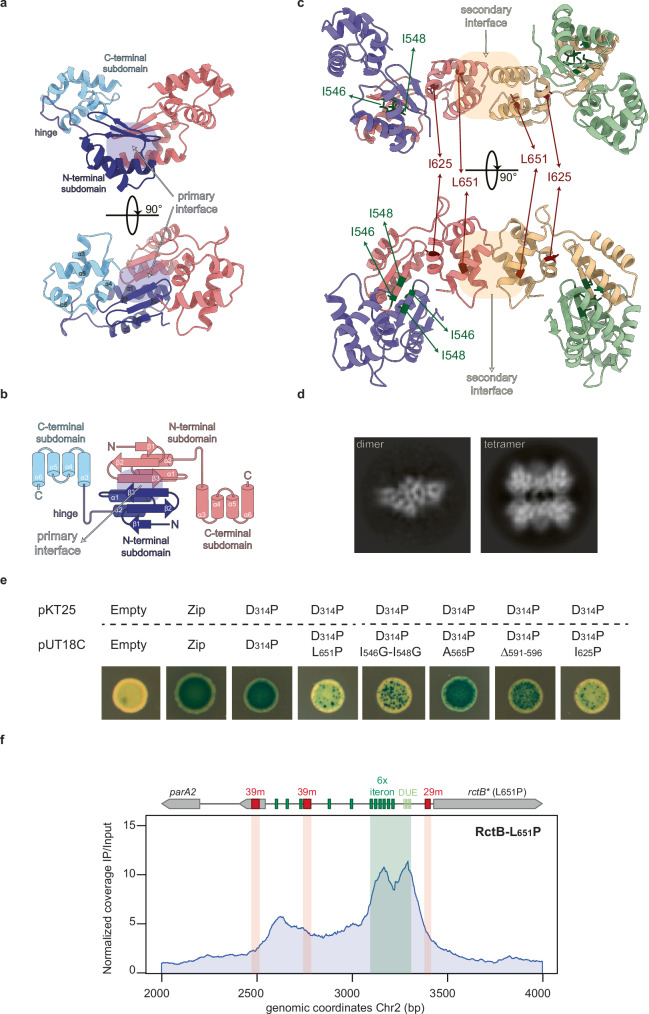
RctB domain IV structure reveals two dimerization interface involved in 29/39m-mediated inhibition. **a** Crystal structure of the RctB^IV^ dimer. Each monomer is composed of an αβ N-terminal subdomain (dark blue) connected to a four α-helices bundle (light blue) via a hinge region. **b** Topological representation of RctB^IV^ dimer. The primary dimer interface (blue shadow) is formed by the extension of the central β-sheet of each monomer through β3 and the antiparallel interactions of the stacking α1. **c** Crystallographic tetramer formed through lattice contacts mediated by the C-terminal subdomain of neighboring dimers. Important residues contributing to the primary interface are shown in dark green. The secondary interface (yellow shadow) with the I625 and L651 functionally relevant residues shown in red. **d** 2D class averages of RctB obtained from cryo-EM **e** Bacterial-Two-hybrid of RctB-D314P vs. RctB domain IV mutants. Empty (negative control), Zip (positive control). Blue colonies indicate a positive protein-protein interaction, while white colonies indicate no interaction. Any blue dots within white colonies are due to *lacZ*+ revertant and should not be considered as evidence of interaction. **f** ChIP-seq of the RctB-L651P mutant at *ori2*. Same legend as for Fig. 2a.

The C-terminal region of RctB, named domain IV (RctB^IV^), is critical to downregulate Chr2 copy number through its involvement, directly or indirectly, in handcuffing activities and binding to 29/39 m^15,16,29^. It is also crucial for *crtS*-mediated control of Chr2 replication^16^. In vitro, RctB is able to self-oligomerize when bound to DNA through interactions mediated by domain IV^16,23^. Thus, we hypothesized that the coordination of Chr1 and Chr2 replication likely depends on the ability of RctB to oligomerize on *ori2* via domain IV. While RctB has remained refringent to structural studies, likely due to the presence of intrinsically unstructured regions in the protein, the structure of domain I and domains II-III have been solved^14,30^. Therefore, we used a truncated version of RctB to determine the structure of domain IV^14,30^ and generated the stable fragment RctB^IV^ (amino acids 532 to 658) that could be used in structural biology. The structure of RctB^IV^ reveals that it is organized into two structural subdomains connected by an extended linker or hinge region (Figs. 3a, Supplementary Fig. 8a). The N-terminal subdomain consists of a 3-stranded β-sheet stacking two α-helices and the C-terminal subdomain has 4 α-helices in a bundle (Fig. 3b). In the crystal, RctB^IV^ retains a dimeric association, confirmed by Size Exclusion Chromatography (SEC) (Supplementary Fig. 8b). This dimerization is primarily mediated by a β-sheet (β3) spanning residue 545 to 549 and involves an interface of 392.4 Å^2^ (Fig. 3a-b, primary interface). The crystal lattice contacts of the C-terminal subdomain suggest the presence of a secondary interaction interface involving 315.4 Å^2^, which could mediate further oligomerization via this region (Fig. 3c, secondary interface). A DALI^31^ search confirmed the C-terminal subdomain as a protein recognition domain capable of forming transient homodimeric interfaces, with Z-scores between 3.7 and 5.4. This secondary interface closely resembles a representative structural homolog detected by DALI (a putative transcription regulator from *Neisseria gonorrhoeae*)^32^. Cryo electron microscopy on full length RctB shows two main class averages which, given their dimensions, are consistent with the dimeric and tetrameric forms of RctB, respectively (Fig. 3d). These findings, together with the presence of multiple intermolecular interfaces in RctB^IV^, inferred from the X-ray structure, suggest that RctB^IV^ may associate in multiple ways and potentially allows RctB to form higher-order oligomeric structures.

To further explore the quaternary structural arrangement of the full-length RctB in the context of our structure of domain IV, we used AlphaFold 3^33^ to calculate the structure of the full length RctB dimer. As shown in Supplementary Fig. 9a–c the predicted dimeric structure recapitulates both the previous dimerization interface via domains II^14^ and the dimerization via domain IV (this work). The formation of this oligomeric structure involves a total interface of 4327 Å^2^. This dimeric arrangement suggests the homodimer is likely a very stable state. The two independent dimerization cores perfectly superpose with the X-ray structures of the truncated domains, which are connected by highly flexible interdomain linking regions. Indeed, SEC of pure full length RctB, shows a complex pattern of two peaks consistent with the dimeric and tetrameric forms of RctB (Supplementary Figs. 9d–e).

### Domain IV dimerization interfaces mediate RctB oligomerization

To further characterize these interfaces, we used the bacterial two-hybrid assay in *E. coli*^34^. We performed a structure-guided point mutation analysis to probe the potential interfaces involved in the oligomerization of RctB^IV^. Given that RctB has a separate dimerization interface in domain II^14^, we substituted residue D314 with a proline (D_314_P) to prevent domain II-mediated interactions that would obscure RctB^IV^-mediated interactions (as done in ref. ^16^). In Fig. 3e, blue colonies (D_314_P/D_314_P) indicated that one RctB molecule could interact with other RctB molecule through another domain, as already reported to be via domain IV^16^. Our present results showed that substitutions in the β3 strand of RctB^IV^ caused the formation of white colonies (D_314_P/D_314_P-I_546_G-I_548_G), indicating that β3 is important for RctB interactions while substitutions that destabilize the α1 helix appear to have little impact on dimerization as evidenced by the formation of blue colonies (D_314_P/D_314_P-A_565_P). Deletion of the linker connecting the two subdomains (591-596), gives blue colonies (D_314_P/D_314_P-∆_591_-_596_) but to a lesser extent than the A_565_P substitution, suggesting that RctB self-association may be sensitive to local structural rearrangements between the two subdomains. The I_625_P and L_651_P substitutions in two different α-helices (α3 and α6) of the C-terminal subdomain also abrogate RctB^IV^-RctB^IV^ interaction. Thus, RctB^IV^ can form homodimers via two different regions: at a primary interface formed between the β3 sheets and at the secondary interface between α-helices. Collectively these results suggest that RctB^IV^ could be an anchor not only for dimerization but also for the formation of transient oligomeric structures when bound to DNA or even to bridge two chromosomal regions.

### RctB oligomerization is required for the binding to inhibitory sites at *ori2*

To better understand the role of RctB^IV^-mediated interactions on the genome-wide in vivo binding of RctB, we performed ChIP-seq of RctB-L_651_P (Fig. 3f). These analyzes revealed a completely different binding profile to *ori2* compared with RctB wt (Fig. 2a). RctB-L_651_P binding to *ori2* is fully shifted to the iterons array and the DUE and is absent from the 29/39 m sites. However, we observed similar binding profile at *crtS* between RctB-L_651_P and RctB wt (Supplementary Fig. 10a). This suggests that, although not involved in DNA binding, RctB^IV^ likely mediates the differential recognition of 29/39 m inhibitory sites relative to iterons or *crtS*, probably via the formation of alternative oligomeric structures. As a control, we performed ChIP-seq of RctB-L_651_P in a ∆*dam* context. As expected, in the absence of Dam methylation, no binding of RctB-L_651_P to *ori2* was observed, although RctB still bound to *crtS* (Supplementary Fig. 10b). Based on these results, we propose that intermolecular domain IV mediated RctB interactions are crucial for the inhibition of replication at *ori2* through the bridging of 29/39 m sites.

### *crtS* exclusively affects the RctB inhibition complex organization at *ori2* to promote initiation

**Fig. 4:**
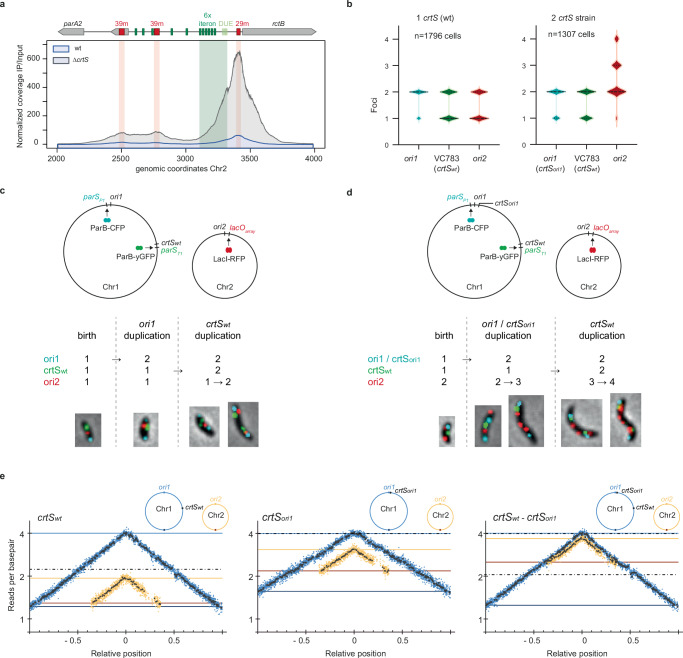
Stoichiometric relationship between *crtS* replication and *ori2* initiation. **a** ChIP-seq of RctB at *ori2* in a *V. cholerae* Δ*crtS* mutant compared to wt. **b** Live fluorescence microscopy in *V. cholerae* with 1 *crtS* (at the native locus) and with 2 *crtS* (at the native locus and near *ori1*). Violin plot showing the distribution of cells with 1, 2, 3, and 4 foci. n=Total number of cells analyzed. **c**, **d** Schematic representation of binding sites for fluorescent proteins that were inserted near *ori1*, VC783 (near *crtS*) and *ori2*. Representative images of cells at different stages of their replication cycle. Cyan foci (*ori1*), Green foci (*crtS*_*WT*_), Red foci (*ori2*). Detailed snapshots analyzes are in Supplementary Fig. 14. **e** MFA of exponentially growing *V. cholerae* cultures using a corrected reference sequence of Chr1^12^. For comparison, MFA from wt (crtS_WT_) and mutant with relocated *crtS* near ori1 (crtS_ori1_) from^12^ are displayed. Sequencing data from mutant with two *crtS* sites (crtS_WT_ – crtS_ori1_) are from this study. Log2 of the number of reads starting at each base (normalized against reads from a stationary phase wt control) is plotted against their relative position on Chr1 (in blue) and Chr2 (in yellow). Positions of ori1 and ori2 are set to 0. Any window containing repeated sequences is omitted; thus, the large gap observed in the right arm of Chr2 consists of filtered repeated sequences. Blue dots (Chr1) and yellow dots (Chr2) indicate the averages of 1000-bp windows; black dots indicate the averages of 10,000-bp windows. Dark blue, light blue, orange, and yellow lines indicate ori1, ter1, ori2, and ter2 numbers of reads, respectively. The dashed black lines indicate the number of reads of the loci where the *crtS* sites are located. Supplementary movies 1, 2, and 3 further explain these MFA analyzes.

The triggering mechanism of *ori2* replication by *crtS* remains poorly understood. In a ∆*crtS* strain, Chr2 is under-initiated, resulting in cell filamentation and lower Chr2 copy number than Chr1^12^. To better understand the consequences of the loss of *crtS*, we conducted a ChIP-seq analysis of RctB in that strain (Fig. 4a). In this background, RctB also predominantly binds to the 29/39 m inhibitory sites at *ori2*, but the ChIP signal was approximately 10-fold higher, indicating a much stronger inhibition of replication in the absence of *crtS*. Based on our new findings, we propose that *crtS* could trigger the initiation of Chr2 replication by destabilizing the nucleoprotein complex that impedes *ori2* initiation. When RctB carried the L651P mutation, there was no difference in its binding profile at *ori2*, irrespective of the presence or absence of *crtS*, indicating that *crtS* solely impacts the binding of RctB to the 29/39 m (Supplementary Fig. 10a). Given that RctB binds to other 39m-like sites outside of *ori2* (Supplementary Data 1), we hypothesized that the absence of *crtS* could enhance the binding of RctB to these sites as well. However, we observed no increase in RctB binding to the other 39 m in the ∆*crtS* mutant (Supplementary Fig. 11). This suggests that *crtS* has an exclusive activity on *ori2*. To investigate this further, we examined RctB binding on *crtS*-containing DNA fragments by TEM. The binding of RctB on *crtS* was restricted to the site and induced a kink in the DNA (Supplementary Fig. 12a). When we mixed DNA substrates containing unmethylated *ori2* and *crtS* with RctB, we detected sporadic RctB-mediated contacts between *ori2* and *crtS* (less than 1% of observed complexes) (Supplementary Fig. 12b), suggesting the possibility of interactions between RctB molecules bound to *crtS* and the ones bound to 29/39 m inhibitory sites. These findings align with the high frequency of contacts between chromosomal regions proximal to *crtS* and *ori2*, observed in vivo by chromosome conformation capture^12^.

### The coupled replication of *crtS* and activation of *ori2* is stoichiometric

Past studies^12,23,35^ demonstrated that introducing an additional *crtS* copy into Chr1 increases Chr2 copy number. To delve deeper into this relationship, we focused on strains with two *crtS* sites. First, we inserted one or two copies of *crtS* into the *att*Tn7 site (located near VC0487 on Chr1) and deleted the endogenous *crtS* site. As anticipated, the strain with two *crtS* exhibited a higher Chr2 copy number compared to the strain with a single *crtS* when measured on stationary phase culture (1.7 vs. 1) (Supplementary Fig. 13a). However, when comparing the ChIP-seq profiles of RctB at *ori2* between these strains, we did not observe any significant differences; in both cases, RctB mainly binds to the 29/39 m sites (Supplementary Fig. 13b). This observation indicates that having two copies of *crtS* does not alter the binding of RctB to activate replication at *ori2*. Consequently, any activation of *ori2* after *crtS* replication probably occurs transiently. To gain a temporal perspective, we used fluorescence microscopy to observe a *V. cholerae* mutant with two distant *crtS* sites^12^ (Fig. 4b-d). This strain contains the endogenous site (*crtS*_*wt*_) and a second site added next to *ori1 (crtS*_*ori1*_*)*—thus delaying the replication timing of both *crtS* sites. We tracked the dynamics of the two *crtS* sites and *ori2* within the cell using three distinct fluorescent DNA-binding proteins, each specifically targeted to their binding sites in the genome: ParB_P1_-CFP bound to the parS_P1_ site near *crtS*_*ori1*_, ParB_pMT1_-yGFP bound to the parS_pMT1_ site near *crtS*_*wt*_, and LacI-RFP bound to the lacO array near *ori2*^36^. Compared to the wild type, which contains only one or two foci of *ori2*, the two-*crtS* containing mutant has up to four *ori2* foci (Fig. 4b). We tracked the longitudinal position of *ori2* foci relative to the Chr1 sites in growing cells and observed that the two-*crtS* containing cells are born with two *ori2* foci. As cell size increases, we observed a sequential increase in the number of *ori2* foci, going from two to three and then to four *ori2* (Figs. 4c,4d, Supplementary Fig. 14). These results suggest that the early replication of *crtS*_*ori1*_ triggers the replication of only one of the two *ori2*. The replication of the second site *crtS*_*wt*_ activates the initiation of the second *ori2*.

We took a closer look at the stoichiometry by Marker Frequency Analysis (MFA) on three exponentially growing strains with different *crtS* contents: the wild type (*crtS*_*wt*_), one mutant with *crtS* relocated at *ori1* (*crtS*_*ori1*_), and the two-*crtS* containing mutant (*crtS*_*wt*_, *crtS*_*ori1*_) (Fig. 4e). We confirmed that the position of *crtS* on Chr1 affects the timing of replication initiation of Chr2, and thereby influences the copy number of Chr2. In the strain with *crtS*_*wt*_, the Chr2 copy number is 0.484, while in the strain with *crtS*_*ori1*_, it is 0.601 (Fig. 4e, left and center), consistent with previous observations^12^. The strain with two *crtS* sites (*crtS*_*wt*_ and *crtS*_*ori1*_) have an *ori2* copy number of 0.900 closely approximating the sum of the *ori2* copy numbers in the single *crtS* strains: *crtS*_*wt*_ and *crtS*_*ori1*_ (0.484 + 0.601). These findings support a model in which each *crtS* duplication event specifically activates one single *ori2*. This model is illustrated in Supplementary movie 1 (*crtS*_*wt*_), movie 2 (*crtS*_*ori1*_) and movie 3 (*crtS*_*wt*_, *crtS*_*ori1*_).

### Replication of *crtS* promotes the transient binding of RctB to replication-activating sites at *ori2*

Based on these results, we hypothesized that to activate replication at *ori2*, RctB should interact with the iterons in a transient way, while remaining bound to the inhibitory 29/39 m sites for most of the cell cycle. Our previous approach did not allow us to capture short-lived events. Therefore, we implemented the ChIP-seq of RctB in a replication-synchronized population to monitor the RctB binding profile over the course of the cell cycle. Similar to *E. coli*, an exponentially growing population of *V. cholerae* can be synchronized using serine hydroxamate (SHX, a serine analog that chemically stimulates the stringent response)^37–39^. During treatment with SHX, replication forks can still progress but the initiation of new rounds of replication is hindered. Transfer of these cells to a fresh medium (without SHX) allows a theoretical synchronous restart of replication. Indeed, previous work showed that after SHX treatment, *V. cholerae* Chr1 re-initiates first and then Chr2^38^ presumably due to control by *crtS*^12^. In our hands, the addition of SHX to 1.5 mg/mL final concentration to a mid-log growing culture of *V. cholerae* (OD_600_ = 0.5) caused an instantaneous growth arrest (Supplementary Fig. 15a). To ensure completion of ongoing replication, we measured the ratio of loci at the origin and terminus of Chr1 (ori1/ter1) by dPCR (Supplementary Fig. 15b). After 180 min of SHX treatment, all cells successfully completed replication with a ratio of ori1/ter1 = 1 meaning that Chr1 was fully replicated. Once SHX was removed, the cells restart to replicate within a time window of 20-30 minutes, similar to what was observed^39^. While SHX synchronization in *V. cholerae* is not perfect and does not achieve full synchronization, it is a useful method for studying replication dynamics in this organism. We used this method to arrest replication initiation, allow completion of the replication cycle, and then re-initiate replication. This approach enabled us to perform ChIP-seq of RctB at different time points after SHX removal (15, 30, 45, 60 minutes) to track the binding cycle of RctB.

**Fig. 5:**
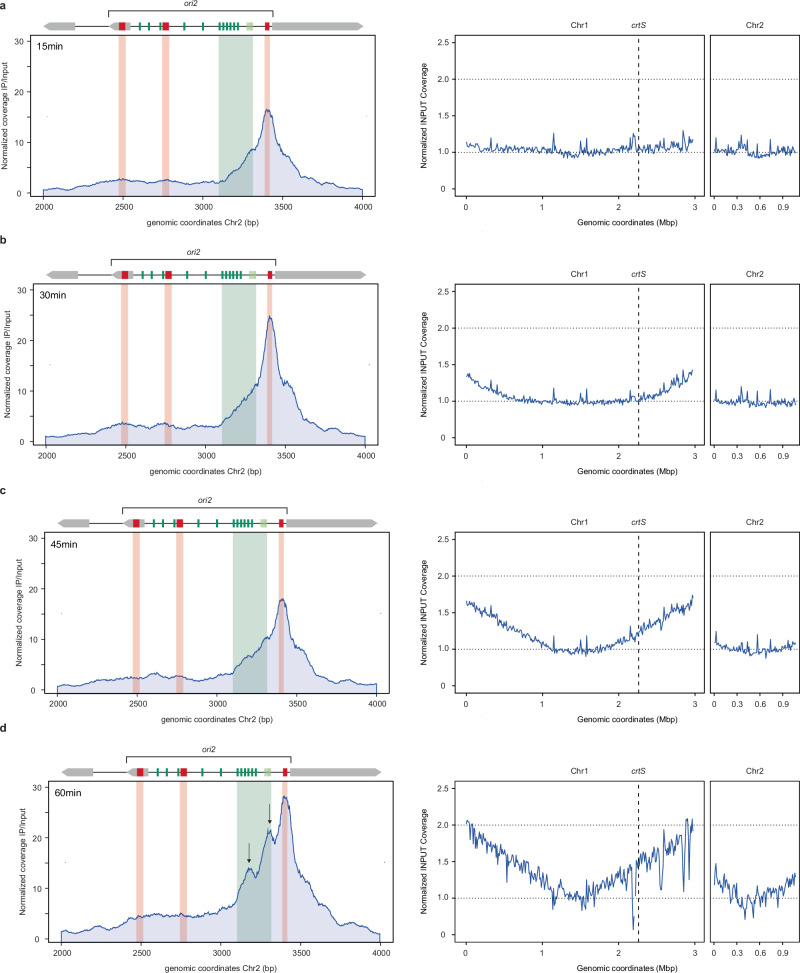
RctB binding pattern on *ori2* shift toward iterons after *crtS* replication. **a**–**d** Left Panel: RctB ChIP-seq performed at different time points after 180 minutes of SHX treatment on a mid-log phase bacterial culture (OD600nm = 0.5), followed by resuspension in fresh M9 medium at recovery intervals of 15, 30, 45, and 60 minutes (**a** = 15 min, **b** = 30 min, **c** = 45 min, **d** = 60 min). The *y*-axis represents normalized RctB ChIP-seq coverage (IP/Input), with genomic coordinates of Chr2 (CP028828.1) on the x-axis. The progressively increasing signal on the iteron and DUE regions is marked by black arrows. Right Panel: Marker Frequency Analysis of input samples, showing replication progression. Coverage is plotted on the y-axis, with genomic coordinates of Chr1 (CP028827.1) and Chr2 (CP028828.1) on the x-axis. The curves are smoothed using 10 kb bins. Coverage values are normalized to the *ter1* region. In non-replicating cells, the MFA is expected to globally remain flat at 1, while in replicating cells, the ori1 value reaches 2, and the ter1 value remains at 1.

Using reads from the input file (control without immunoprecipitation), we performed MFA to track replication fork progression on both chromosomes (Fig. 5a–d, right panel). Within the 15 minutes after removal of SHX (Fig. 5a), replication has not restarted, as shown by the flat MFA profile of Chr1 and Chr2, RctB predominantly binds to 29/39 m on *ori2*. After 30 minutes (Fig. 5b), Chr1 starts replicating and at 45 minutes (Fig. 5c) the *crtS* site seems to be just replicated (the Ter region of Chr1 still appears not replicated), while Chr2 replication has not yet started. At both time points, RctB ChIP-seq patterns at *ori2* is nearly unchanged (Fig. 5a-d, left panel). At 60 minutes (Fig. 5d), Chr2 is finally replicating. On the ChIP-seq profile, two additional peaks appear: one on the six-iteron array and one on the DUE (Fig. 5d, black arrows). Here, we detected a change in RctB binding dynamics at *ori2* over time, where RctB initially interact with inhibitory sites (29/39 m). Subsequently, there is an increased engagement of RctB with activating sites (iteron) and the DUE. This shift suggests that rather than completely dissociating from the inhibitory sites, RctB molecules undergo a structural rearrangement that alleviates the inhibition, making the iterons and DUE accessible. This rearrangement likely disrupts the inhibitory loop, facilitating the opening of *ori2* and initiating Chr2 replication. As expected, this process is triggered after *crtS* replication on Chr1. We did not observe fluctuations in the binding of RctB at *crtS* before or after the passage of the replication fork, nor during stationary versus exponential phase of growth (Supplementary Figs. 3 & Supplementary Fig. 16). Thus, RctB binds similarly to *crtS* throughout the cell cycle.

## Discussion

Our work provides cell-cycle-integrated insights into the synchronized replication of Vibrio’s two chromosomes. We identified an inhibition complex at *ori2* that prevents replication initiation for a significant portion of the cell cycle. We also highlighted the pivotal role of *crtS* in temporarily disrupting this complex, enabling Chr2 replication. Based on the binding dynamics of RctB throughout the cell cycle, we propose a model for the regulated control of Chr2 replication. In this model, *ori2* is inherently bistable, with the replication of *crtS* acting as a decisive ON switch. Before *crtS* replication, *ori2* is sequestered by an inhibition complex, with RctB bridging the 29/39 m sites, and inducing DNA loops that effectively keep *ori2* in an OFF state. The replication of *crtS* destabilizes this complex, causing transient exposure of *ori2* to an ON state compatible with replication initiation. This shift promotes the recruitment of RctB to the six iterons, the DUE, and ultimately, the initiation of Chr2 replication.

A crucial feature of the stabilization of the OFF state is the oligomerization properties of RctB via the domain IV which defines the formation of the 29/39 m bridges and precludes Chr2 replication. In a broader context, analogous types of nucleoprotein complexes have been observed intermolecularly in the regulation of iteron plasmid copy number, involving the initiators RepA_pPS10_^40^ and Pi_R6K_^41^. In our model, we propose that initiation silencing occurs through intramolecular DNA looping at the origin. This is akin to the mechanism in the F plasmid, where RepE initiators establish a bridge between the iterons of the negative control element (*incC*) and those of the origin (*oriF*) that further inhibit the formation of an open complex^42^. Vibrio distinguishes itself by having tailored specific 29/39 m sites to serve this regulatory role.

**Fig. 6:**
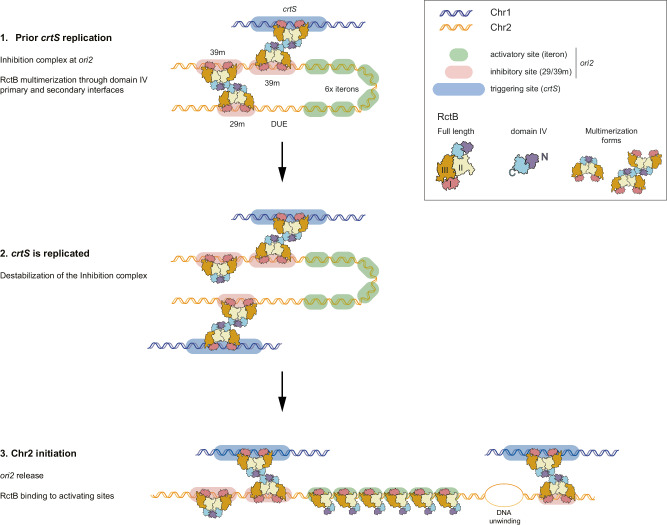
Model for the Cell-Cycle-Integrated Control of Chr2 Replication in *V. cholerae.* **1** Prior *crtS* replication, *ori2* is sequestered by an inhibition complex, formed by RctB multimers bridging 29/39 m sites. RctB oligomerization domain IV is involved in the formation of this nucleoprotein complex. **2** Upon *crtS* replication, we hypothesize that the second copy of *crtS* destabilizes the inhibition complex, possibly at the domain IV secondary interface. **3** *Ori2* is thus released and the iteron array becomes accessible for RctB binding. This leads to the opening of the DUE, as shown^18^.

The replication of *crtS* triggers a shift in RctB binding favoring interactions with the iterons and DUE, conditions that are compatible with *ori2* initiation. Further mechanistic research is needed to explore the molecular interactions between RctB and DNA, as well as how *crtS* disrupts the inhibition complex. However, our model is consistent with the bistable state of *ori2* being influenced by *crtS* replication, the structural properties of RctB and the dramatic changes in the DNA structure and organization induced by RctB. Given that *crtS* specifically influences RctB binding at *ori2*, without affecting other genomic sites, and considering the stoichiometric relationship between *crtS* replication and *ori2* activation, our data strongly suggest a direct physical interaction leading to *ori2* activation by *crtS*. This is consistent with our previous observations of frequent inter-chromosomal interactions proximal to *crtS* and *ori2*^12^. Moreover, we observed that the regulatory control exerted by *crtS* over *ori2* is lost when a single 29 m or 39 m site is mutated^16^. This synergistic effect implies that *crtS* likely disrupts the 29/39 m bridging, rather than affecting each site individually. We propose that each newly duplicated *crtS* site have RctB bound to it. Both *crtS*-RctB complexes can each interact with one 29/39m-RctB complex. These interactions destabilize the DNA loop which inhibit *ori2* initiation. Once *ori2* is exposed, the iteron array and DUE become accessible, so that free RctB molecules can bind and initiate replication (Fig. 6).

In contrast to plasmids, where initiators bind solely to iterons, RctB capacity to interact with multiple sites offers a more refined control over replication initiation. This multifaceted binding ability is determined by factors such as methylation-dependent binding to iterons^20^, domain IV-oligomerization-dependent binding to 29/39 m (this study), and Lrp-enhanced binding to *crtS*^43,44^. All these factors introduce dynamic binding behaviors throughout the cell cycle, ensuring that Chr2 replication is finely tuned and synchronized with other cellular processes.

Other Bacterial species with multipartite genome, such as *Agrobacterium tumefaciens*^9^ and *Pseudoalteromonas spongiae*^8^, exhibit synchronous termination of secondary chromosome replication with that of the primary chromosome, as demonstrated by marker frequency analysis (MFA) during exponential growth. This suggests that coordinated replication timing between chromosomes is a conserved evolutionary feature in species with multiple chromosomes. The mechanisms we have uncovered for the regulation of the secondary chromosome in *V. cholerae* raise interesting questions about how plasmids were domesticated and controlled as they evolved into stable secondary chromosomes. Future studies could further explore these evolutionary parallels by conducting ChIP-seq experiments on the initiators of secondary chromosomes in other bacteria. Investigating whether these initiators bind to checkpoints on the primary chromosome and whether their binding dynamics vary throughout the cell cycle, could shed light on the evolutionary transition from plasmid to chromosome. Such research would expand our understanding of how bacteria have evolved to stably maintain and regulate multipartite genomes, offering insights into the broader principles of genome organization and evolution.

## Methods

### Strains and growth conditions

Vibrio cholerae N16961 El tor (7PET) is the strain studied here. As a heterologous system for dPCR experiments Escherichia coli K12 MG1655 was also used. Different *E.coli* strains were used for cloning DH5α, TOP10, Π3813 and β3914^45^ for conjugation. LB (Luria Broth) was used as a standard rich medium for both *E. coli* and *V. cholerae*.

### Strains and plasmids constructions

*V. cholerae* N16961 and *E. coli* MG1655 strains were genetically modified using conditionally replicative suicide vectors, as described in ref. ^46^. Suicide vectors carrying R6K origin are constructed in *E. coli* pi3813 that express the Pir protein necessary for plasmid replication. Plasmids are then transformed to donor strain *E. coli* b3914 which encodes chromosomally the RP4 conjugation machinery and the Pir protein.

For conjugation, both donor and recipient strains were cultured separately until reaching an OD600 of 1. Cultures were mixed and spotted on LB agar plates supplemented with diaminopimelic acid (DAP), followed by incubation at 37 °C for 3 hours to allow conjugation. Afterward, plasmid integration was selected on LB plates containing chloramphenicol (Cm, 5 µg/mL) and 1% glucose. To induce plasmid excision, cells were grown in LB with 1% glucose and subsequently plated on LB agar containing 0.2% arabinose. Strain modifications were verified by PCR using DreamTaq polymerase (Thermo Fisher Scientific) and confirmed by Sanger sequencing. DNA fragments were amplified using Phusion Master Mix (Thermo Fisher Scientific). Plasmids were constructed using Gibson assembly^47^. For cases requiring specific point mutations, these were introduced via PCR using oligonucleotides with the desired mutations incorporated at the 5’ end. For ChIP-seq experiment (on RctB and on ParB2), we replaced the wild-type alleles on the chromosome with a C-terminal 3xFLAG through allelic exchange. For the dPCR experiments, we introduced RctB secondary binding sites into the *lacZ* gene of MG1655, we took an extending of 250 base pairs on each side of the peak detected from ChIP-seq analysis. Subsequently, these modified strains were transformed with a pORI2 plasmid, following the procedure outlined in ref. ^23^. Plasmids and strains used in this study are listed in Supplementary Table 1 and Supplementary Table 2.

### High Resolution ChIP-seq

ChIP-seq were performed as described in ref. ^48^ in *V. cholerae* expressing protein-3xFLAG under its native promoter. We confirmed that 3xFLAG did not impair protein function by dPCR measurement of Chr2 copy number. Cells were grown in 100 mL Mueller Hinton (MH) media with shaking at 37 °C until they reach mid-log growth phase (OD_600_ 0.5). Then cells were fixed by crosslinking with 1% formaldehyde for 30 min at room temperature under agitation, followed by quenching with 0.5 M glycine for 15 min. Bacteria were harvested, washed two times in ice-cold Tris-Buffered saline (20 mM Tris/HCl pH 7.5, 150 mM NaCl) and pellets were stored at -80 °C. Pellets were lyzed in buffer containing lysozyme and protease inhibitor pill (cOmplete Protease Inhibitor, Roche). Chromatin was then fragmented by sonication (Covaris S220) for in milliTUBE (1 ml with AFA Fiber). Correct DNA fragmentation centered around 300 bp was confirmed using 1.8% agarose gel electrophoresis. Following sonication, protein-DNA complexes were immunoprecipitated with anti-FLAG magnetic beads (M8823 Sigma). Correct immunoprecipitation was confirmed by western blot using anti-FLAG antibodies (F7425 Sigma). Once recovered, immunoprecipitated DNA was de-crosslink at 65 °C overnight. Then DNA was blunt-ended, A-tailed, ligated to adapters, amplified by PCR (13 cycles) and purified using TruSeq ChIP Library Preparation Kit (illumina) and AMPureXP beads (Beckman Colter). ChIP-seq libraries quality was measured by Agilent Bioanalyzer 2100 instrument using a DNA High Sensitivity chip. Finally, libraries were sequenced using a MiniSeq Mid Output Kit on a Miniseq sequencing machine (Illumina). Paired-end reads from two independent RctB ChIP-seq experiments were mapped to *Vibrio cholerae* N16961 reference genome (Chr1: CP028827.1 and Chr2: CP028828.1) with Bowtie2 using galaxy.pasteur platform. Peak calling was performed using MACS2, where aligned reads were significantly enriched compared to a control (strain without FLAG). Identified peaks were checked by hand, using IGV genome browser to confirm correct peak assignment. MEME-ChIP (version 5.4.1) online browser was used for motif enrichment finding. For normalization, the IP and INPUT files were first adjusted to the same number of reads using R (Supplementary Software). Then the coverage value for each position in the IP file was divided by the average coverage value of a 2 kb sliding window from the INPUT file.

The genomic replication pattern of the different strains was examined using a computational approach based on Marker Frequency Analysis (MFA). The reads from the INPUT sample (not immunoprecipitated DNA) of the ChIP-seq data were mapped to *V. cholerae* N16961 reference genome (Chr1: CP028827.1 and Chr2: CP028828.1) using BOWTIE2, the resultant mapping was saved in two separate Binary Alignment Map (BAM) files corresponding to the two chromosomes. Subsequently, a R script was used for data processing and visualization. The depth of coverage was extracted from the BAM files using the Rsamtools package. The genome was divided into 10 kbp bins, and the average coverage for each bin was computed. Coverage values were then normalized with respect to the average coverage of the termination (ter) region, which is located at the midpoint of the first chromosome (+/- 5 kb). Coverage values more than three standard deviations from the mean were considered outliers and removed from the dataset. For visualization, a ggplot2-based plot was generated, showing the normalized coverage for each bin against the genomic position in Mbp. Linear regression analysis was performed separately on each half of the chromosomes, and the best-fit lines were superimposed on the plot (Supplementary Fig. 17).

### Marker frequency analysis

Grow conditions, DNA extraction, sequencing and analysis for MFA was done as described previously^12^. *V. cholerae* cells were grown in LB medium supplemented with 1 % glucose at 30 °C under agitation until reaching exponential phase (OD450 ~ 0.15). For each sample, 30 mL of culture was collected to isolate genomic DNA (gDNA). Genomic DNA was extracted using the DNeasy® Tissue Kit (Qiagen) following the manufacturer’s protocol. The extracted gDNA was quantified using a Qubit Fluorometer (Life Technologies). After DNA sonication and library generation were prepared, and more than 5 million unique reads per sample were generated on an Ion Proton sequencer (Life Technologies) using PI chips, with an average read length around 100 bp. Data available at the European Nucleotide Archive (Supplementary Information).

### Digital PCR quantification (dPCR)

Quantifications of (*ori1* and *ori2*) in *V. cholerae* and (*oriC* and pORI2) in *E. coli* were performed as described in ref. ^16^ using multiplex dPCR (Stilla Technologies). dPCR was performed directly on cell lysate: 1 mL of overnight culture was washed twice with 1 mL PBS, pellets were then frozen at −20 °C and resuspended in 200uL PBS, samples were then boiled for 10 min. Samples were centrifuge for 10 min, supernatant was recovered, and DNA content was measured using Qubit device (ThermoFisher). PCR reactions were performed with 0.1 ng of DNA using the PerfeCTa MultiPlex qPCR ToughMix (Quantabio) on a Sapphire chip (Stilla Technologies). Digital PCR was conducted on a Naica Geode and Image acquisition on the Naica Prism3 reader. Images were analyzed Crystal Miner software (Stilla Technologies). The dPCR run was performed using the following steps: droplet partition (40 °C, atmospheric pressure AP to + 950 mbar, 12 min), initial denaturation (95 °C, +950 mbar, for 2 min), followed by 45 cycles at (95 °C for 10 s and 60 °C for 30 s), droplet release (down 25 °C, down to AP, 33 min).

### Digital PCR quantification of RNA (RT-dPCR)

Quantification of RctB mRNA was performed using multiplex digital RT-dPCR (Stilla Technologies)^49^. Primers and probes are listed in Supplementary Table 3. *V. cholerae* RNA was prepared as described in ref. ^50^. *Vibrio cholerae* cells were grown in 10 mL LB medium at 37 °C to an OD600nm = 0.5. A 2 mL aliquot of cells was collected, pelleted by centrifugation, and lysed by resuspension in TRIzol™ (Invitrogen). The lysates were stored at −20 °C until further processing. RNA extraction was performed using the RNeasy® Kit (Qiagen), following the manufacturer’s instructions. To remove residual DNA, RNA samples were treated with Turbo DNase™ (Invitrogen). For RT-dPCR, reactions were set up with 1 ng of total RNA using the qScriptTM XLT One-Step RT-qPCR ToughMix® (Quantabio). RT-dPCR and data analysis were conducted as described above for dPCR. The RT-dPCR run was performed in the following steps: droplet partition (40 °C, AP to +950 mbar, 12 min), cDNA synthesis (50 °C, +950 mbar, 10 min), initial denaturation (95 °C, +950 mbar, for 1 min), followed by 45 cycles at (95 °C for 10 s and 60 °C for 15 s), droplet release (down 25 °C, down to AP, 33 min). Expression values were normalized to the expression of the housekeeping gene *gyrA* as described in ref. ^50^.

### SHX *Vibrio cholerae* synchronization

*V. cholerae* expressing RctB-3xFLAG from its natural promoter was streaked from a −80 °C stock onto an MH agar plate. An isolated colony was then picked and cultured overnight in M9 media containing 0.2% casamino acids and 0.4% glucose at 30 °C. Cells from this overnight culture were subsequently diluted 100-fold in fresh media and grown in 40 mL of M9 media with 0.2% casamino acids and 0.4% glucose at 30 °C. When the cells reached an OD600nm = 0.5, they were treated with SHX (DL-serine hydroxamate, Sigma) at a final concentration of 1.5 mg/mL for 180 minutes. This treatment prevented new rounds of initiation and allowed cells to complete ongoing replication. Cells stalled in G1 were then centrifuged at 6,000 g for 15 minutes, and the pellets were resuspended in 40 mL of fresh M9 media before being returned to 30 °C for growth. Cells were fixed with 1% formaldehyde at various time points: 15, 30, 45, and 60 minutes after the wash. Finally, samples were processed according to the ChIP-seq protocol.

### Transmission electron microscopy

Five different DNA fragments were used containing different combination of RctB binding sites (F1 39mL-39mR-29m; F2 39mL-39mR; F3 29 m; F4 crtS; F5 random DNA). DNA fragments were produced using PCR reaction with Phusion Master Mix (Thermo) and then purified using AMPure XP beads (Beckman Colter) (primers used are listed in Supplementary Table 3). RctB, DnaK and DnaJ proteins were produced and purified as in PMC6212839. Incubation: RctB (1200 nM) was first pre-incubated with DnaK (100 nM) and DnaJ (100 nM) in 20 µL final buffer of 10 mM Tris-HCl pH 7.5, 50 mM NaCl, 1 mM MgCl2 in presence of 100 µM ATP, 10 min at 4 °C. RctB at 120 nM final was then incubated with 1,4 nM DNA molecule in the same binding buffer 2 or 30 min at 20 °C. Positive staining and darkfield imaging mode: 5 μL drop of the incubation reaction was deposited on a 600-mesh copper grid previously covered with a thin carbon film and preactivated by glow discharge in the presence of amylamine (Sigma-Aldrich, France). The grids were rinsed and positively stained with 2% (w/v) aqueous uranyl acetate, carefully dried with filter paper, and observed in crystallographic darkfield mode in zero-loss filtered imaging using a Zeiss 912 transmission electron microscope. Images were captured at 85,000× magnification with a Tengra CCD camera and analyzed with iTEM software (both Olympus Soft Imaging Solution). Nucleoprotein complexes analysis: Mapping of the binding localization was performed based on the asymmetry of the positions of RctB binding sites (crtS or 29/39 m). For measurement, the DNA image was divided into segments of approximately 10 nm. The presence or absence of RctB in each segment is indicated as a percentage.

### Expression and purification of RctB and RctB^IV^ for crystallization and biophysical studies

RctB and were RctB^IV^ cloned into a pET-24b plasmid downstream an N-terminal 10xHis_SUMO tag. BL21 *E.coli* cells were transformed with these constructs for expression and purification. Transformed cells were first grown at 37 °C until reaching an OD ~ 0.6 then expression was induced with 0.25 mM Isopropyl β-D-1-thiogalactopyranoside (IPTG) overnight at 16 °C. Afterwards cells were harvested by centrifugation at 3000 *g*. For purification, the cell lysate supernatants were loaded onto a Ni-Sepharose column. In both cases, proteins were eluted using a step gradient of imidazole. The fractions containing the protein of interest were then loaded into a Citiva 60/160 Seperdex 200 size exclusion chromatography column, equilibrated in 50 mM Hepes pH 7.5, 500 mM NaCl, 500 mM KCl, 2 mM MgCl_2_ and 1 mM TCEP. The 10xHis-SUMO tag was removed by incubating overnight the protein with the protease UlpI in a 1:100 molar ratio. The tag-free RctB and RctB^IV^ were then isolated from the cleaved tag and UlpI by loading the mixture into a Co-sepharose column and collecting the flow-through that contained the tag-free samples. For further studies RctB and RctB^IV^ were extensively dialyzed against 50 mM Hepes pH 7.5, 200 mM NaCl, 2 mM MgCl_2_ and 1 mM TCEP.

### RctB and RctB^IV^ analytical size exclusion chromatography (SEC)

Analytical SEC of RctB and RctB^IV^ were carried out on a Superdex 200 10/300 column (Citiva) equilibrated in 50 mM Hepes, 200 mM NaCl, 2 mM MgCl_2_ and 1 mM TCEP. The flow rate was 1 ml/min and the injection volume 0.5 ml. The calibration of the column was performed by running the Biorad gel filtration standards (Cat. No. 151–1901), containing a mixture of bovine thyroglobulin (670 kDa), bovine γ-globulin (158 kDa), chicken ovalbumin (44 kDa), equine myoglobin (17 kDa) and vitamin B12 (1.35 kDa).

### RctB^IV^ crystallization and structure determination

The screening of crystallization conditions of RctB^IV^ was carried out using the sitting-drop vapor-diffusion method with highly pure samples of RctB^IV^ at a concentration of 8 mgmL^-1^. The drops were set up in Swiss (MRC) 96-well two-drop UVP sitting-drop plates using the Mosquito HTS system (TTP Labtech). Drops of 0.1 μL protein and 0.1 μL precipitant solution were equilibrated to 80 μL precipitant solution in the reservoir. Commercially available screens PACT premier, LMB and SG1 (Molecular Dimensions) were used to test crystallization conditions. The condition resulting in protein crystals (PACTpr screen position E3: 200 mM NaI, 20 % PEG 3350) were repeated as 2 µL drops. Crystals were harvested using suitable cryo-protecting solutions (consisting of the mother liquor supplemented with 20% to 25% of glycerol) and vitrified in liquid N_2_ for transport and storage before X-ray exposure.

X-ray diffraction data was collected at the SOLEIL synchrotron (Gif-sur-Yvette, Paris, France) on the Proxima 1 (PX1) beamlines using an Eiger-X 16 M detector. Native crystals typically diffracted to 1.8 Å – 2.0 Å resolution. We screened a collection of heavy atoms for experimental phasing and observed that the combination of quick soaks (under 30 s) with NaBr at 1.5 M, and longer soaks (20 min to 30 min) in AgNO3 (10 mM) and GdCl (10 mM) provided sufficient isomorphous signal for experimental phasing. For this, data were collected at the peak and inflexion point of the Br edge and Ag and Gd K-edge. After phase improvement with Pirate and density modification with Parrot, the maps were used initially used in Buccaneer for model building and then as starting point for the MR-Rosetta^51^ suit which completed one third of the structure. Further cycles of automated model building with AutoBuild from the Phenix package^52^ extended the model to 90%. After several iterations of manual building with Coot^53^ and maximum likelihood refinement as implemented in Buster/TNT^54^, the model was extended to cover all the residues (R/Rfree of 19.2%/26.7 %). Supplementary Table 4 details all the X-ray data collection and refinement statistics.

### Structure prediction

The prediction of the dimeric structure of full-length RctB was performed with AlphaFold 3^33^. The calculation was done using the full sequence of the protein and the default parameters of the program.

### Single particle cryo-electron microscopy (cryo-EM)

For the cryo-EM experiments RctB was purified in 50 mM Hepes, 200 mM NaCl, 2 mM MgCl_2_ and 1 mM TCEP. RctB (3 μl) was applied to gold Quantifoil grids (UltrAuFoil 1.2/1.3 Au 300) under 100 % relative humidity at 10 °C at 0.25 mg/ml. Two grids were blotted for 2 and 3 seconds, respectively, and plunged into liquid ethane using a Vitrobot IV (ThermoFisher Scientific). Particles were imaged from the aforementioned grids on a Glacios microscope (ThermoFisher Scientific) operating at 200 kV with a Falcon 4i direct electron detector. In total, 3800 micrographs, with 0.96 Å/pxl, were collected using a defocus range from −1 μm to −3 μm and total dose of 50 e^-^/Å^2^. The micrographs movies were motion corrected using MotionCor2^55^, and contrast transfer function, CTF, were estimated using CTFFind-4.1^56^. Particles were picked using Topaz^57^ and subjected to reference free 2D classification using cryoSPARC^58^. Several rounds of 2D classifications rendered a total of 133108 particles.

### Live-cell fluorescence microscopy

The genes encoding for the CFP-ParB_P1_, yGFP-ParB_pMT1_ and LacI-RFP-T fluorescent DNA binding proteins were inserted into the *V. cholerae* chromosome^36,59,60^. Their cognate binding sites were inserted near *ori1* (*parS*_*P1*_), near VC783 (*parS*_*pMT1*_), and near *ori2* (*lacO* array). For microscopy observations, cultures were prepared following the procedures described in ref. ^12^. Initially, strains were streaked onto MH plates from a -80 °C stock and then grown overnight in MH rich liquid media. The following day, cultures were diluted at a ratio of 1:1000 in M9 with 1% fructose and grown to exponential phase (OD_600_ = 0.2). A 2 µL aliquot of this culture was spotted on an agar pad (M9, 1% fructose, 1% agarose), for microscopy observation. Cell imaging was performed using an Axio Observer 7 inverted videomicroscope (Zeiss). Analysis of the acquired snapshots was conducted using Fiji software with the MicrobeJ plugin^61^.

### Bacterial two hybrid

BTH101 cells were co-transformed with plasmids encoding T18 and T25 fused to RctB variants following the method described in refs. ^34,62^ and plated on LB agar plates containing kanamycin (25 µg/mL) and carbenicillin (100 µg/mL). Approximatively 500 co-transformants were pooled, resuspended in PBS, diluted to OD_600_ = 1 and spotted (10 µL) onto LB agar plates containing kanamycin (25 µg/mL), carbenicillin (100 µg/mL), Xgal (40 µg/mL) and IPTG (0.5 mM). Plates were incubated for 48 h at 30 °C, followed by an additional 24 h at 4 °C. Blue spots indicate an interaction between the tested proteins and white spots indicate no interaction. Empty pKT25 and pUT18C vectors were used as negative control, and pKT25T-Zip/pUT18C-Zip pair was used as positive control.

### Reporting summary

Further information on research design is available in the Nature Portfolio Reporting Summary linked to this article.

## Supplementary information

Supplementary Information

Reporting Summary

Description of Additional Supplementary Files

Supplementary Data 1

Supplementary Movie 1

Supplementary Movie 2

Supplementary Movie 3

Supplementary Software

Transparent Peer Review file

## Source data

Source Data

## Data Availability

The sequencing data generated in this study have been deposited in the European Nucleotide Archive under project accession code PRJEB63079 (https://www.ebi.ac.uk/ena/browser/view/PRJEB63079). For ChIPseq negative control (no FLAG tag): ERR12420106. For RctB ChIP-seq in exponential phase: ERR12420290 and ERR12651384 (IP) - ERR12420289 and ERR12651383 (INPUT). For RctB ChIP-seq in stationary phase: ERR12420287 and ERR12651392 (IP) and ERR12420288 and ERR12651391 (INPUT). For RctB ChIP-seq in 29 m C > A mutated context: ERR12420285 and ERR12651382 (IP) - ERR12420286 and ERR12651381 (INPUT). For ParB2 ChIP-seq: ERR12420283 and ERR12651380 (IP) - ERR12420284 and ERR12651379 (INPUT). For RctB-L651P ChIP-seq in wt context: ERR12420292 and ERR12651386 (IP) - ERR12420291 and ERR12651385 (INPUT). For RctB ChIP-seq in ∆crtS context: ERR12421767 and ERR12651396 (IP) -ERR12421766 and ERR12651395 (INPUT). For RctB-L651P ChIP-seq in ∆crtS context: ERR12492377 and ERR12651390 (IP) - ERR12492376 and ERR12651389 (INPUT). For RctB ChIP-seq in a context with crtS relocated to attTN7 site: ERR12421765 (IP) and ERR12421764 (INPUT). For RctB ChIP-seq in a context with two crtS sites inserted in attTN7 site, ERR12421763 (IP) and ERR12421762 (INPUT). For RctB ChIP-seq in ∆dam context: ERR12493891 and ERR12651394 (IP) - ERR12493890 and ERR12651393 (INPUT). For RctB-L651P ChIP-seq in ∆dam context ERR12493889 and ERR12651388 (IP) and ERR12493888 and ERR12651387 (INPUT). For RctB ChIP-seq in synchronized population: 15 min after release ERR12421789 and ERR13771528 (IP) - ERR12421788 and ERR13771529 (INPUT), 30 min after release ERR12421791 and ERR13771530 (IP) and ERR12421790 and ERR13771531 (INPUT), 45 min after release ERR12421793 and ERR13771532 (IP) - ERR12421792 and ERR13771533 (INPUT), 60 min after release ERR12421795 and ERR13771534 (IP) - ERR12421794 and ERR13771535 (INPUT). For MFA analysis: wt strain ERR12492335, mutant strain with crtS near ori1 ERR12492336 and mutant with two crtS sites at wt and at ori1 ERR12492337. The structural data for RctB domain IV have been deposited in the Protein Data Bank (PDB) under entry 8RV3. All additional data generated in this study are provided in the Source Data file. Source data are provided with this paper.

## References

[1] Reyes-Lamothe, R. & Sherratt, D. J. The bacterial cell cycle, chromosome inheritance and cell growth. *Nat. Rev. Microbiol.* **17**, 467–478 (2019). 31164753 10.1038/s41579-019-0212-7

[2] Beaufay, F., Coppine, J. & Hallez, R. When the metabolism meets the cell cycle in bacteria. *Curr. Opin. Microbiol.* **60**, 104–113 (2021). 33677348 10.1016/j.mib.2021.02.006

[3] Fournes, F., Val, M. E., Skovgaard, O. & Mazel, D. Replicate once per cell cycle: replication control of secondary chromosomes. *Front. Microbiol.* **9**, 1833 (2018). 30131796 10.3389/fmicb.2018.01833 PMC6090056

[4] Du, W. L. et al. Orderly replication and segregation of the four replicons of Burkholderia cenocepacia J2315. *PLoS Genet.* **12**, e1006172 (2016). 27428258 10.1371/journal.pgen.1006172 PMC4948915

[5] Frage, B. et al. Spatiotemporal choreography of chromosome and megaplasmids in the Sinorhizobium meliloti cell cycle. *Mol. Microbiol.* **100**, 808–823 (2016). 26853523 10.1111/mmi.13351

[6] Deghelt, M. et al. G1-arrested newborn cells are the predominant infectious form of the pathogen Brucella abortus. *Nat. Commun.* **5**, 4366 (2014). 25006695 10.1038/ncomms5366 PMC4104442

[7] Dubarry, N., Willis, C. R., Ball, G., Lesterlin, C. & Armitage, J. P. In Vivo Imaging of the Segregation of the 2 Chromosomes and the Cell Division Proteins of Rhodobacter sphaeroides Reveals an Unexpected Role for MipZ. *mBio* **10**, 10.1128/mBio.02515-18 (2019). 10.1128/mBio.02515-18 PMC6315104 30602584

[8] Xie, B. B. et al. Evolutionary Trajectory of the Replication Mode of Bacterial Replicons. *mBio* **12**, 10.1128/mBio.02745-20 (2021). 10.1128/mBio.02745-20 PMC7858055 33500342

[9] Ren, Z. et al. Conformation and dynamic interactions of the multipartite genome in Agrobacterium tumefaciens. *Proceedings of the National Academy of Sciences of the United States of America* **119**, 10.1073/pnas.2115854119 (2022). 10.1073/pnas.2115854119 PMC8833148 35101983

[10] Rasmussen, T., Jensen, R. B. & Skovgaard, O. The two chromosomes of Vibrio cholerae are initiated at different time points in the cell cycle. *EMBO J.* **26**, 3124–3131 (2007). 17557077 10.1038/sj.emboj.7601747 PMC1914095

[11] Baek, J. H. & Chattoraj, D. K. Chromosome I controls chromosome II replication in Vibrio cholerae. *PLoS Genet.* **10**, e1004184 (2014). 24586205 10.1371/journal.pgen.1004184 PMC3937223

[12] Val, M. E. et al. A checkpoint control orchestrates the replication of the two chromosomes of Vibrio cholerae. *Sci. Adv.* **2**, e1501914 (2016). 27152358 10.1126/sciadv.1501914 PMC4846446

[13] Egan, E. S. & Waldor, M. K. Distinct replication requirements for the two Vibrio cholerae chromosomes. *Cell* **114**, 521–530 (2003). 12941279 10.1016/s0092-8674(03)00611-1

[14] Orlova, N. et al. The replication initiator of the cholera pathogen’s second chromosome shows structural similarity to plasmid initiators. *Nucleic acids Res.* **45**, 3724–3737 (2017). 28031373 10.1093/nar/gkw1288 PMC5397143

[15] Jha, J. K., Demarre, G., Venkova-Canova, T. & Chattoraj, D. K. Replication regulation of Vibrio cholerae chromosome II involves initiator binding to the origin both as monomer and as dimer. *Nucleic acids Res.* **40**, 6026–6038 (2012). 22447451 10.1093/nar/gks260 PMC3401445

[16] Fournes, F. et al. The coordinated replication of Vibrio cholerae’s two chromosomes required the acquisition of a unique domain by the RctB initiator. *Nucleic acids Res.* **49**, 11119–11133 (2021). 34643717 10.1093/nar/gkab903 PMC8565311

[17] Niault, T., Czarnecki, J., Lambérioux, M., Mazel, D. & Val, M. E. Cell cycle-coordinated maintenance of the Vibrio bipartite genome. *EcoSal Plus* **11**, 10.1128/ecosalplus.esp-0008-2022 (2023). 10.1128/ecosalplus.esp-0008-2022 PMC10729929 38277776

[18] Chatterjee, S., Jha, J. K., Ciaccia, P., Venkova, T. & Chattoraj, D. K. Interactions of replication initiator RctB with single- and double-stranded DNA in origin opening of Vibrio cholerae chromosome 2. *Nucleic acids research*, 10.1093/nar/gkaa826 (2020). 10.1093/nar/gkaa826 PMC7641748 33035310

[19] Wegrzyn, K. E., Gross, M., Uciechowska, U. & Konieczny, I. Replisome assembly at bacterial chromosomes and iteron plasmids. *Front. Mol. Biosci.* **3**, 39 (2016). 27563644 10.3389/fmolb.2016.00039 PMC4980987

[20] Demarre, G. & Chattoraj, D. K. DNA adenine methylation is required to replicate both Vibrio cholerae chromosomes once per cell cycle. *PLoS Genet.* **6**, e1000939 (2010). 20463886 10.1371/journal.pgen.1000939 PMC2865523

[21] Venkova-Canova, T. & Chattoraj, D. K. Transition from a plasmid to a chromosomal mode of replication entails additional regulators. *Proc. Natl Acad. Sci. USA* **108**, 6199–6204 (2011). 21444815 10.1073/pnas.1013244108 PMC3076835

[22] Venkova-Canova, T., Saha, A. & Chattoraj, D. K. A 29-mer site regulates transcription of the initiator gene as well as function of the replication origin of Vibrio cholerae chromosome II. *Plasmid* **67**, 102–110 (2012). 22248922 10.1016/j.plasmid.2011.12.009 PMC3319240

[23] de Lemos Martins, F., Fournes, F., Mazzuoli, M. V., Mazel, D. & Val, M. E. Vibrio cholerae chromosome 2 copy number is controlled by the methylation-independent binding of its monomeric initiator to the chromosome 1 crtS site. *Nucleic acids Res.* **46**, 10145–10156 (2018). 30184118 10.1093/nar/gky790 PMC6212839

[24] Bailey, T. L., Johnson, J., Grant, C. E. & Noble, W. S. The MEME Suite. *Nucleic acids Res.* **43**, W39–W49 (2015). 25953851 10.1093/nar/gkv416 PMC4489269

[25] Menikpurage, I. P., Woo, K. & Mera, P. E. Transcriptional activity of the bacterial replication initiator DnaA. *Front. Microbiol.* **12**, 662317 (2021). 34140937 10.3389/fmicb.2021.662317 PMC8203912

[26] Venkova-Canova, T., Baek, J. H., Fitzgerald, P. C., Blokesch, M. & Chattoraj, D. K. Evidence for two different regulatory mechanisms linking replication and segregation of vibrio cholerae chromosome II. *PLoS Genet.* **9**, e1003579 (2013). 23818869 10.1371/journal.pgen.1003579 PMC3688505

[27] Val, M. E. et al. Fuse or die: how to survive the loss of Dam in Vibrio cholerae. *Mol. Microbiol.* **91**, 665–678 (2014). 24308271 10.1111/mmi.12483

[28] Benureau, Y. et al. Method combining BAC film and positive staining for the characterization of DNA intermediates by dark-field electron microscopy. *Biol. Methods Protoc.* **5**, bpaa012 (2020). 32913896 10.1093/biomethods/bpaa012 PMC7474861

[29] Jha, J. K., Ghirlando, R. & Chattoraj, D. K. Initiator protein dimerization plays a key role in replication control of Vibrio cholerae chromosome 2. *Nucleic acids Res.* **42**, 10538–10549 (2014). 25159619 10.1093/nar/gku771 PMC4176361

[30] Jha, J. K. et al. The DnaK Chaperone Uses Different Mechanisms To Promote and Inhibit Replication of Vibrio cholerae Chromosome 2. *mBio* **8**, 10.1128/mBio.00427-17 (2017). 10.1128/mBio.00427-17 PMC5395669 28420739

[31] Holm, L., Laiho, A., Toronen, P. & Salgado, M. DALI shines a light on remote homologs: one hundred discoveries. *Protein Sci.: a Publ. Protein Soc.* **32**, e4519 (2023). 10.1002/pro.4519 PMC9793968 36419248

[32] Das, D. et al. The structure of the first representative of Pfam family PF09836 reveals a two-domain organization and suggests involvement in transcriptional regulation. *Acta Crystallogr Sect. F. Struct. Biol. Cryst. Commun.* **66**, 1174–1181 (2010). 20944208 10.1107/S1744309109022672 PMC2954202

[33] Abramson, J. et al. Accurate structure prediction of biomolecular interactions with AlphaFold 3. *Nature* **630**, 493–500 (2024). 38718835 10.1038/s41586-024-07487-w PMC11168924

[34] Karimova, G., Pidoux, J., Ullmann, A. & Ladant, D. A bacterial two-hybrid system based on a reconstituted signal transduction pathway. *Proc. Natl Acad. Sci. USA* **95**, 5752–5756 (1998). 9576956 10.1073/pnas.95.10.5752 PMC20451

[35] Ramachandran, R., Ciaccia, P. N., Filsuf, T. A., Jha, J. K. & Chattoraj, D. K. Chromosome 1 licenses chromosome 2 replication in Vibrio cholerae by doubling the crtS gene dosage. *PLoS Genet.* **14**, e1007426 (2018). 29795553 10.1371/journal.pgen.1007426 PMC5991422

[36] Dalia, A. B. & Dalia, T. N. Spatiotemporal analysis of DNA integration during natural transformation reveals a mode of nongenetic inheritance in bacteria. *Cell* **179**, 1499–1511 e1410 (2019). 31835029 10.1016/j.cell.2019.11.021 PMC6913884

[37] Ferullo, D. J., Cooper, D. L., Moore, H. R. & Lovett, S. T. Cell cycle synchronization of Escherichia coli using the stringent response, with fluorescence labeling assays for DNA content and replication. *Methods* **48**, 8–13 (2009). 19245839 10.1016/j.ymeth.2009.02.010 PMC2746677

[38] Kemter, F. S. et al. Synchronous termination of replication of the two chromosomes is an evolutionary selected feature in Vibrionaceae. *PLoS Genet.* **14**, e1007251 (2018). 29505558 10.1371/journal.pgen.1007251 PMC5854411

[39] Kemter, F. S. et al. Stringent response leads to continued cell division and a temporal restart of DNA replication after initial shutdown in Vibrio cholerae. *Mol. Microbiol.* **111**, 1617–1637 (2019). 30873684 10.1111/mmi.14241

[40] Molina-Garcia, L. et al. Functional amyloids as inhibitors of plasmid DNA replication. *Sci. Rep.* **6**, 25425 (2016). 27147472 10.1038/srep25425 PMC4857107

[41] McEachern, M. J., Bott, M. A., Tooker, P. A. & Helinski, D. R. Negative control of plasmid R6K replication: possible role of intermolecular coupling of replication origins. *Proc. Natl Acad. Sci. USA* **86**, 7942–7946 (1989). 2682632 10.1073/pnas.86.20.7942 PMC298188

[42] Zzaman, S. & Bastia, D. Oligomeric initiator protein-mediated DNA looping negatively regulates plasmid replication in vitro by preventing origin melting. *Mol. cell* **20**, 833–843 (2005). 16364910 10.1016/j.molcel.2005.10.037

[43] Ciaccia, P. N., Ramachandran, R. & Chattoraj, D. K. A requirement for global transcription factor lrp in licensing replication of vibrio cholerae chromosome 2. *Front. Microbiol.* **9**, 2103 (2018). 30250457 10.3389/fmicb.2018.02103 PMC6139311

[44] Doan, A. et al. The replication enhancer crtS depends on transcription factor Lrp for modulating binding of initiator RctB to ori2 of Vibrio cholerae. *Nucleic acids research*, 10.1093/nar/gkad1111 (2023). 10.1093/nar/gkad1111 PMC10810183 38000366

[45] Le Roux, F., Binesse, J., Saulnier, D. & Mazel, D. Construction of a Vibrio splendidus mutant lacking the metalloprotease gene vsm by use of a novel counterselectable suicide vector. *Appl. Environ. Microbiol.* **73**, 777–784 (2007). 17122399 10.1128/AEM.02147-06 PMC1800747

[46] Val, M. E., Skovgaard, O., Ducos-Galand, M., Bland, M. J. & Mazel, D. Genome engineering in Vibrio cholerae: a feasible approach to address biological issues. *PLoS Genet.* **8**, e1002472 (2012). 22253612 10.1371/journal.pgen.1002472 PMC3257285

[47] Gibson, D. G. et al. Enzymatic assembly of DNA molecules up to several hundred kilobases. *Nat. methods* **6**, 343–345 (2009). 19363495 10.1038/nmeth.1318

[48] Diaz, R. E., Sanchez, A., Anton Le Berre, V. & Bouet, J. Y. High-resolution chromatin immunoprecipitation: ChIP-sequencing. *Methods Mol. Biol.* **1624**, 61–73 (2017). 28842876 10.1007/978-1-4939-7098-8_6

[49] Madic, J. et al. Three-color crystal digital PCR. *Biomolecular detection quantification* **10**, 34–46 (2016). 27990348 10.1016/j.bdq.2016.10.002 PMC5154636

[50] Lo Scrudato, M. & Blokesch, M. The regulatory network of natural competence and transformation of Vibrio cholerae. *PLoS Genet.* **8**, e1002778 (2012). 22737089 10.1371/journal.pgen.1002778 PMC3380833

[51] Terwilliger, T. C. et al. phenix.mr_rosetta: molecular replacement and model rebuilding with Phenix and Rosetta. *J. Struct. Funct. Genomics* **13**, 81–90 (2012). 22418934 10.1007/s10969-012-9129-3 PMC3375004

[52] Afonine, P. V. et al. Towards automated crystallographic structure refinement with phenix.refine. *Acta Crystallogr. Sect. D., Biol. Crystallogr.* **68**, 352–367 (2012). 22505256 10.1107/S0907444912001308 PMC3322595

[53] Emsley, P. & Cowtan, K. Coot: model-building tools for molecular graphics. *Acta Crystallogr. Sect. D., Biol. Crystallogr.* **60**, 2126–2132 (2004). 15572765 10.1107/S0907444904019158

[54] Smart, O. S. et al. Exploiting structure similarity in refinement: automated NCS and target-structure restraints in BUSTER. *Acta Crystallogr. Sect. D., Biol. Crystallogr.* **68**, 368–380 (2012). 22505257 10.1107/S0907444911056058 PMC3322596

[55] Zheng, S. Q. et al. MotionCor2: anisotropic correction of beam-induced motion for improved cryo-electron microscopy. *Nat. methods* **14**, 331–332 (2017). 28250466 10.1038/nmeth.4193 PMC5494038

[56] Rohou, A. & Grigorieff, N. CTFFIND4: Fast and accurate defocus estimation from electron micrographs. *J. Struct. Biol.* **192**, 216–221 (2015). 26278980 10.1016/j.jsb.2015.08.008 PMC6760662

[57] Bepler, T. et al. Positive-unlabeled convolutional neural networks for particle picking in cryo-electron micrographs. *Nat. methods* **16**, 1153–1160 (2019). 31591578 10.1038/s41592-019-0575-8 PMC6858545

[58] Punjani, A., Rubinstein, J. L., Fleet, D. J. & Brubaker, M. A. cryoSPARC: algorithms for rapid unsupervised cryo-EM structure determination. *Nat. methods* **14**, 290–296 (2017). 28165473 10.1038/nmeth.4169

[59] David, A. et al. The two Cis-acting sites, parS1 and oriC1, contribute to the longitudinal organisation of Vibrio cholerae chromosome I. *PLoS Genet.* **10**, e1004448 (2014). 25010199 10.1371/journal.pgen.1004448 PMC4091711

[60] Woldringh, C. L., Hansen, F. G., Vischer, N. O. & Atlung, T. Segregation of chromosome arms in growing and non-growing Escherichia coli cells. *Front. Microbiol.* **6**, 448 (2015). 26029188 10.3389/fmicb.2015.00448 PMC4428220

[61] Ducret, A., Quardokus, E. M. & Brun, Y. V. MicrobeJ, a tool for high throughput bacterial cell detection and quantitative analysis. *Nat. Microbiol.* **1**, 16077 (2016). 27572972 10.1038/nmicrobiol.2016.77 PMC5010025

[62] Battesti, A. & Bouveret, E. The bacterial two-hybrid system based on adenylate cyclase reconstitution in Escherichia coli. *Methods* **58**, 325–334 (2012). 22841567 10.1016/j.ymeth.2012.07.018

[63] Yu, Y., Ouyang, Y. & Yao, W. shinyCircos: an R/Shiny application for interactive creation of Circos plot. *Bioinformatics* **34**, 1229–1231 (2018). 29186362 10.1093/bioinformatics/btx763

[64] Matthey, N., Drebes Dorr, N. C. & Blokesch, M. Long-Read-Based Genome Sequences of Pandemic and Environmental Vibrio cholerae Strains. *Microbiology resource announcements* **7**, 10.1128/MRA.01574-18 (2018). 10.1128/MRA.01574-18 PMC6298558 30574591

[65] Heidelberg, J. F. et al. DNA sequence of both chromosomes of the cholera pathogen Vibrio cholerae. *Nature* **406**, 477–483 (2000). 10952301 10.1038/35020000 PMC8288016

