# Supplementary material for: Dynamic transitions of initiator binding coordinate the replication of the two chromosomes in Vibrio cholerae
Source: Nat Commun. 2025 Jan 8;16:485. doi: 10.1038/s41467-024-55598-9 (PMC11711613; doi:10.1038/s41467-024-55598-9)
Supplement: Supplementary file 1 — Supplementary Information [file 41467_2024_55598_MOESM1_ESM.pdf]

SUPPLEMENTARY INFORMATION

Dynamic transitions of initiator binding coordinate the replication of the two chromosomes in *Vibrio cholerae*

Théophile Niault, Ariel Talavera, Eric Le Cam, Sonia Baconnais, Ole Skovgaard, Florian Fournes, Léa Wagner, Hedvig Tamman, Andrew Thompson, Dannele Echemendia Blanco, Noa Guzzi, Abel Garcia Pino, Didier Mazel and Marie-Eve Val

SUPPLEMENTARY TABLES

Supplementary Table 1. Plasmids

Experiment	Relevant figures	Description	Name / Reference
pORI2-derivatives for copy number monitoring	1c	pSW23T::*ori2*$_{V.cholerae}$, *oriT*$_{RP4}$, *ori*$_{R6K}$, Cm^r	pORI2 [1]
Expression vectors for protein purification	-	pET24b(+)	Novagen
	3a-c	pET24b::*rctBIV*	This study
	3d	pET24b::*rctB*	This study
Expression vectors for Bacterial two-hybrid	3e (pKT25 Empty)	pKT25 (P_{lac}-*cyaA*T25) *ori*$_{p15A}$, Kan^r	pKT25 [2]
	3e (pKT25 Zip)	pKT25-*zip* (Leucine zipper)	pKT25-zip [2]
	3e (pKT25 D314P)	pKT25::*rctB*$_{D314P}$	pFF102 [3]
	3e (pUT18C Empty)	pUT18C (P_{lac}-*cyaA*T18) *ori*$_{ColE1}$, Ap^r	pUT18C [2]
	3e (pUT18C Zip)	pUT18C-*zip* (Leucine zipper)	pUTC18-zip [2]
	3e (pUT18C D314P)	pUT18C:: *rctB*$_{D314P}$	pAT01 [3]
	3e (pUT18C D314P-L651P)	pUT18C:: *rctB*$_{D314P\text{-}L651P}$	pFF128 [3]
	3e (pUT18C D314P-I546G-I548G)	pUT18C::*rctB*$_{D314P\text{-}I546G\text{-}I548G}$	This study
	3e (pUT18C D314P-A565P)	pUT18C::*rctB*$_{D314P\text{-}A565P}$	This study
	3e (pUT18C D314P-Δ591-596)	pUT18C::*rctB*$_{D314P\text{-}\Delta 591\text{-}596}$	This study
	3e (pUT18C D314P-I625P)	pUT18C::*rctB*$_{D314P\text{-}I625P}$	This study
Vectors used for genetic engineering	Suicide plasmid for allele exchange	pMP7 (*oriV*$_{R6K\gamma}$ *oriT*$_{RP4}$ *araC*-P_{BAD}-*ccdB* ; *cat*)	pMP7 (#pSW7848) [4]
	Supp 4	pMP7::parB2-3xFLAG (Cter)	This study
	1a, 2a, 5	pMP7::rctB-3xFLAG (Cter)	This study
	3f	pMP7-*rctB*$_{L651P\text{-}3xFLAG(Cter)}$	This study
	4a (*crtS* excision)	pMP7-[frt-*crtS*-*arr2*-frt]	pMP184 [1]
		ori$_{pSCS101}$ *repA* *oriT*$_{RP4}$ *araC* P_{BAD}-*flp* ; *bla*	pMP108 [1]
	Transposition in *attTn7* site	Tn7 helper – *ori*$_{pSCS101}$ *repA*$_{ts}$ oriT$_{RP4}$ *araC* P_{BAD}-*tnsABCD* ; *bla*	pMVM1 [1]
		Tn7 shuttle – *oriV*$_{R6K\gamma}$ *oriT*$_{RP4}$:: [Tn7R-aadA7-MCS-Tn7L]; cat	pMP234 [3]
		pMP234 :: [Tn7R- aadA7-crtS-Tn7L]	This study
		pMP234:: [Tn7R- aadA7-2xcrtS-Tn7L]	This study

Supplementary Table 2. Bacterial strains

Experiment	Relevant figures	Description	Name (Reference)
Cloning strain	-	E. coli lacIq thi-1 supE44 endA1 recA1 hsdR17 gyrA462 zei-298::Tn10 (Tcr) Δ*thyA*::(*erm-pir*116)	π3813 [5]
Donor strain for conjugation	-	E. coli F- RP4-2-Tc::Mu ΔdapA::(erm-pir) *gyr*A462 *zei*-298::Tn10	β3914 [5]
Bacterial two-hybrid	3e	*E. coli* F−, *cya*-99, *ara*D139, *gal*E15, *gal*K16, *rps*L1, *hsd*R2, *mcr*A1, *mcr*B1	BTH101 [2]
Protein purification	3a-d	E. coli BL21	Thermo Scientific
Vibrio cholerae N16961	-	N16961 *hap*R+	N16961rep [6]
	Supp 5	N16961 Δ*dam* (fused chromosomes)	Δdam#4 [7]
	1e	N16961 Δ*rctB* (fused chromosomes)	MCH1 [4]
Chr2 copy number	1d	N16961 VC1042-aph-VC1043	This study
		N16961 VC0643 inactivated, TTACGCAGAGTG > TTGCGAAGGGTA	This study
		N16961 VC1643 inactivated, TTACGGCTAACG > TCACAGCCAACG	This study
ChIP-seq	1a, 2a, 5<br>Supp 2, 3, 11	N16961, rctB-3xFLAG (Cter)	This study
	2c	N16961, rctB::rctB-3xFLAG (Cter); 29m(C>A)	This study
	3f	N16961, rctB::rctB-L651P-3xFLAG (Cter)	This study
	Supp 4	N16961, parB2::parB2-3xFLAG (Cter)	This study
	Supp 5	Δdam#4, rctB::rctB-3xFLAG (Cter)	This study
	Supp 7	N16961, rctB::rctB-L651P-3xFLAG (Cter); ΔcrtS	This study
	Supp 7	Δdam#4 , rctB::rctB-L651P-3xFLAG (Cter)	This study
	4a<br>Supp 8	N16961, rctB::rctB-3xFLAG (Cter); ΔcrtS	
	Supp 10	N16961, rctB::rctB-3xFLAG (Cter); ΔcrtS; attTn7::crtS	
	Supp 10	N16961, rctB::rctB-3xFLAG (Cter); ΔcrtS; attTn7::2xcrtS	
pORI2 copy number	1c	E. coli MG1655-*rpsL**, Strep^r	J665 [1]
		J665 lacZ::crtS	J666 [1]
		J665 lacZ::VC1042-VC1043	This study
		J665 lacZ::VC1803	This study
		J665 lacZ::VC0643	This study
		J665 lacZ::VC1624	This study
		J665 lacZ::VC1643	This study
		J665 lacZ::VC1757	This study
		J665 lacZ::VC2373	This study
Fluorescent microscopy	4c	N16961[parSP1@VC2759][parST1@VC0783][lacO@VCA1092][lacZ::(lacI-RFP-T,parBT1-yGFP)][attTn7::CFP-parBP1]	This study
	4d	N16961-VC0023-crtS-VC0024 [parSP1@VC2759][parST1@VC0783][lacO@VCA1092][lacZ::(lacI-RFP-T,parBT1-yGFP)][attTn7::CFP-parBP1]	This study
Marker Frequency Analysis	4e	crtS$_{wt}$ (N16961ChapRΔlacZ)	WT [8]
		crtS$_{ori1}$ (N16961ChapRΔlacZ ΔcrtS with crtS inserted between VC0023/VC0024)	crtS$_{VC23}$ [8]
		crtS$_{wt}$-crtS$_{ori1}$ (N16961ChapRΔlacZ with crtS inserted between VC0023/VC0024)	crtS$_{WT/VC23}$ [8]

Supplementary Table 3. Oligonucleotides

Primer and probe sequences used in dPCR and RT-dPCR reactions			
Target	Orientation	Primers (5 '→ 3')	Dual Labeled Probes (5 '→ 3')
ori1 V. cholerae	FWD	GCTGCTCGACAAATGGAAC	[FAM]-TCCGATGGAAATGTTGGTGAAACACATTCT-[BHQ1]
	REV	AAGATGCGGACTGACCAC	
ori2 V. cholerae	FWD	TGTCTCGTCGTCATACCG	[HEX]-ATCTGATCCGCGAACTTCGTCGTCTCT-[BHQ1]
	REV	CTTCACTCCCCTTCCCTTC	
oriC E. coli	FWD	CCACCGAGAAGAACATGGAG	[FAM]-ATTGTCCAGAAGGTGGCTGGGGGGTTTT-[BHQ1]
	REV	GCCGCAGGATTACATAGGAC	
pORI2	FWD	TTATGGTGAAAGTTGGAACCTC	[HEX]-GCCGATCAACGTCTCATTTTCGCCA-[BHQ1]
	REV	GCCGAATAAATACCTGTGACG	
VC1042	FWD	TCAAAATCCATCACCACTTCC	(FAM)-CCTTCACCCATCACATAACGCACACCA-(BHQ1)
	REV	TGCTTACCAAATCTTGCCTC	
VC1803	FWD	TCACTGCAAAGCTATGTGTC	(HEX)-AGCGTTGGTTACGCTTCATAAGACAC-(BHQ1)
	REV	TTTCTTCTCCAGTCAAAAGCC	
gyrA	FWD	AATGTGCTGGGCAACGACTG	[Cy5]-CACCCTCATGGTGACAGTGCGGTTT-[BHQ2]
	REV	GAGCCAAAGTTACCTTGGCC	

Primers used to generate TEM substrates			
DNA Fragment	name	Primers (5 '→ 3')	DNA Matrix
crtS substrate	MV577	GCCAATATCGCACGAATTCC	V. cholerae gDNA
	MV485	TCGCCCATTCACTTGATCCG	
ori2 substrate	MV109	AGGCGTAATGAACCTTGTGG	V. cholerae gDNA
	MV226	CACGCAGTGAGATCAGATTC	
39mL-39mR-29m	MV1002	CCTCTTACGTGCCGATCAAC	pORI2
	MV226	CACGCAGTGAGATCAGATTC	
39mL-39mR	MV1002	CCTCTTACGTGCCGATCAAC	pORI2
	MV1321	GAAAAATTTGTTGTTTTTAAATAACTG	

29m	MV277	GGCGCTGAAGATGTC	pORI2
	MV1399	GATCTCCGGTGGAAATAGG	
Control DNA	#3265	GATACTTCCATGGGCTTCAC	V. cholerae gDNA
	#3266	CACGCAGCAGATGCTGATTC	

Supplementary Table 4. X-ray data collection and processing

The $CC_{1/2}$ criterion was used to determine the resolution range. Values for the outer shell are given in parentheses.

**Sample**	**RctB^{IV}**
Diffraction source	Soleil PX1
Wavelength (Å)	0.9786
Temperature (K)	100.0
Detector	Eiger-X 16M
Crystal-detector distance (mm)	332.4
Rotation range per image (°)	0.1
Exposure time per image (s)	0.01
Space group	$P2_12_12$
a, *b*, *c* (Å)	64.9 94.8 47.6
α, β, γ (°)	90.0 90.0 90.0
Mosaicity (°)	0.2
Resolution range (Å)	53.55 – 2.34
Total N°. of reflections	145328 (7004)
N°. of unique reflections	11993 (611)
Completeness (%)	93.8 (95.6)
Redundancy	12.1 (11.5)
$\langle I/\sigma(I)\rangle$	10.6 (2.8)
$CC_{1/2}$	0.99 (0.89)
R_{pim}	0.064 (0.282)
Overall *B* factor / Wilson plot (Å^2)	28.9
R-factor (%)	19.2
R_{free}-factor (%)	26.7
Ramachandran profile (%)	
Core	96.3
Allowed	3.7
Outliers	0.0
R.m.s. deviations	
Bond lengths (Å)	0.023
Bond angles (°)	2.02
Number of atoms	2180
Macromolecules	1917
Solvent	214
Other	49
B-factors (Å2)	
All atoms	46.3
Macromolecules	46.7
Solvent atoms	42.0
Other atoms	49.7
PDB ID	8RV3

SUPPLEMENTARY FIGURES

Supplementary Figure 1. Iteron-type initiators and *ori2* structure

a. Comparison of protein domain organization between Chr2 initiator (RctB) and iteron-type plasmid initiators (RepA, RepE, Pi). The different structural domains are indicated (WH = winged helix). **b.** Representation of Chr2 origin of replication (ori2) and nucleotide sequence of iterons, 29/39m and DNA unwinding element (DUE). RctB binding sites are indicated by dark green (iterons) and red (29/39m) rectangles. *Ori2* contains eleven iterons, six of which are essential for replication, while the others have a regulatory function. The three 29/39m sites act as strong negative regulators of *ori2* initiation. Single-stranded RctB binding sites (5'-ATCA) in the DUE are represented as light green bars. Other protein binding sites are indicated: IBS (IHF binding site), DnaA box, *parS2* (ParB2 binding site). Conserved bases in the sequence alignments are shown in bold. Dam-methylated GATC motifs within the iterons are shown in purple. Within the DUE, RctB binding sites to single-stranded DNA (5'-ATCA) are shown in green.

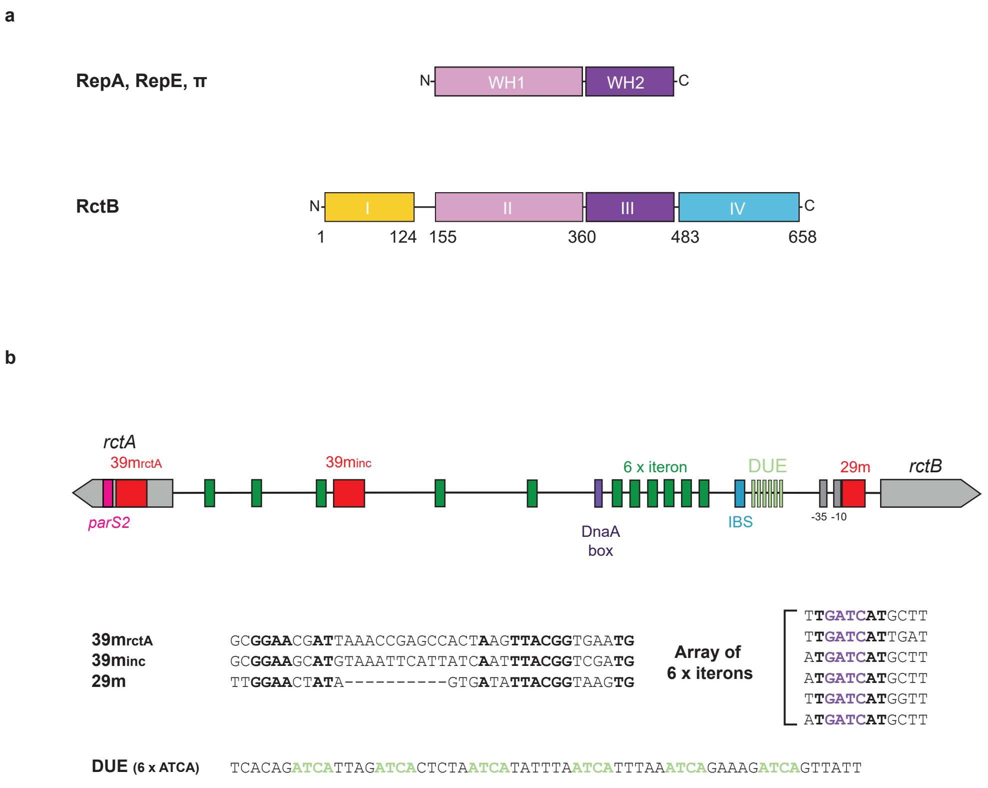

Supplementary Figure 1

Supplementary Figure 2: RctB ChIP Signal across all detected binding loci.

Y-axis: Normalized RctB ChIP Signal (IP Coverage/INPUT Coverage); X-axis: Genomic Coordinates on V. cholerae N16961 Reference Genome (CP028827.1 and CP028828.1). A 2,000-bp window is centered around the peak. ChIP signals are shown in blue, and the genetic context is depicted above the graph with CDS represented by grey arrows.

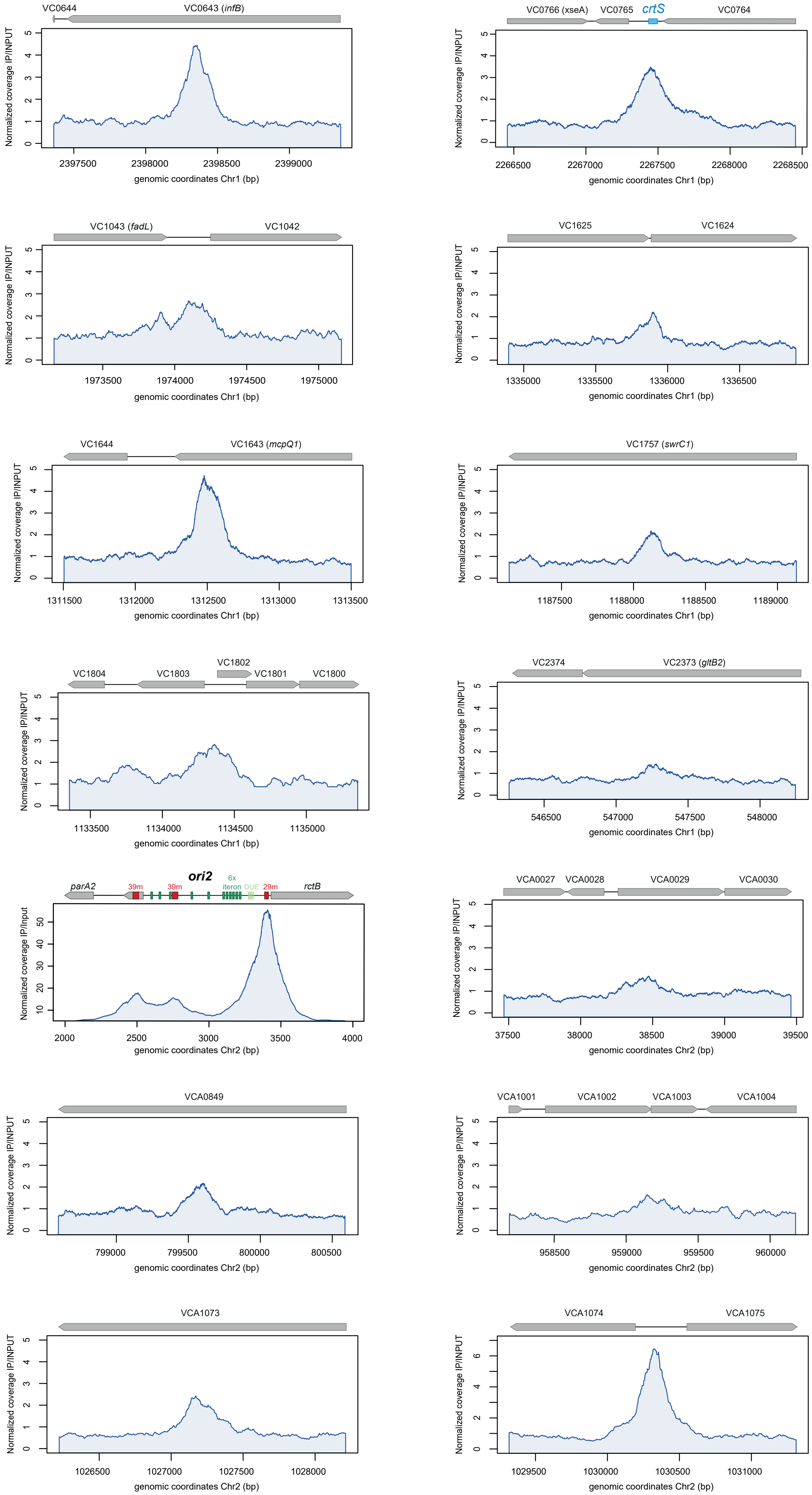

Supplementary Figure 2

Supplementary Figure 3: RctB ChIP profile remains similar between replicating and not replicating states.

On y axis: RctB normalized ChIP signal (IP coverage/INPUT coverage), on x axis: genomic coordinates on *V. cholerae* N16961 reference genome (CP028827.1 and CP028828.1). A 2.000bp window is represented and centered around the peak. ChIP signals are represented in blue for replicating cells (exponential phase - EXP , OD_{600nm}=0.5) and in red for non-replicating cells (stationary phase - STAT , overnight culture). Genetic context (CDS in grey arrows) and known RctB binding sites are depicted on top of the graph: genes as grey arrow, 39mer site as red boxes, iterons as green rectangles, *crtS* as a cyan box.

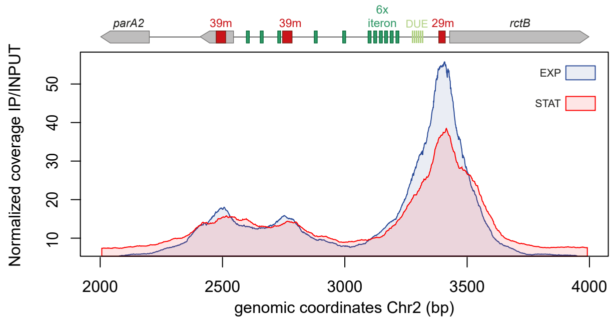

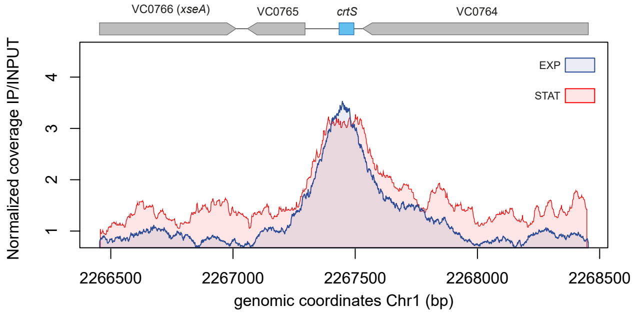

Supplementary Figure 3

Supplementary Figure 4: ParB2 ChIP signal covers the *inc* region of *ori2*.

a. on y axis: coverage from the IP reads, on x axis: genomic coordinates on *Vibrio cholerae* N16961 reference Chr2 (CP028828.1). Raw coverage is represented in pink for ParB2-3xFLAG and in grey for the wt strain (negative control). Already identified *parS* sites [9] are represented on top of the graph. **b.** on left y axis: RctB normalized ChIP signal (IP coverage/INPUT coverage), on right y axis (different scale): ParB2 normalized ChIP signal, on x axis: genomic coordinates on *V. cholerae* N16961 reference Chr2 (CP028828.1). A 2.000bp window containing *ori2* is represented. ChIP signals are represented in blue for RctB and in pink for ParB2. Genetic context (CDS in grey arrows) and known RctB binding sites are depicted on top of the graph: genes as grey arrow, 39mer site as red boxes, iterons as green rectangles and *parS* site as pink rectangle.

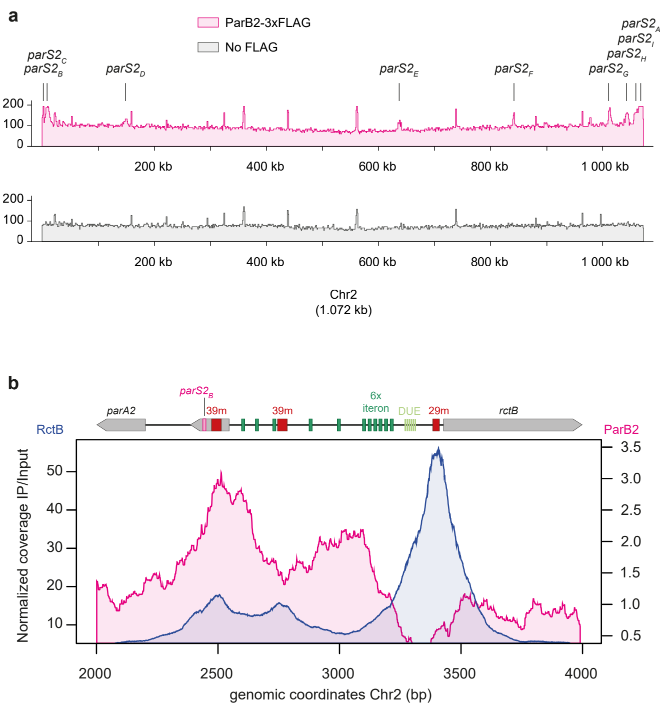

Supplementary Figure 4

Supplementary Figure 5: RctB binding pattern on *ori2* is maintained in absence of Dam methylation.

a. On the left: *V. cholerae* N16961 wt genome organization, on the right: *Δdam* strain genome organization in which *ori2* is inactivated and Chr2 is inserted in Chr1 (mutant Δ*dam*#4 identified in [7]. **b**. on y axis: RctB normalized ChIP signal (IP coverage/INPUT coverage), on x axis: genomic coordinates on *Vibrio cholerae* N16961 reference Chr1 (CP028827.1). A 2.000bp window containing *ori2* is represented. RctB ChIP signal is represented in blue wt strain and in purple for *Δdam* strain. Genetic contexts are depicted on top of the graph as in Fig.2a.

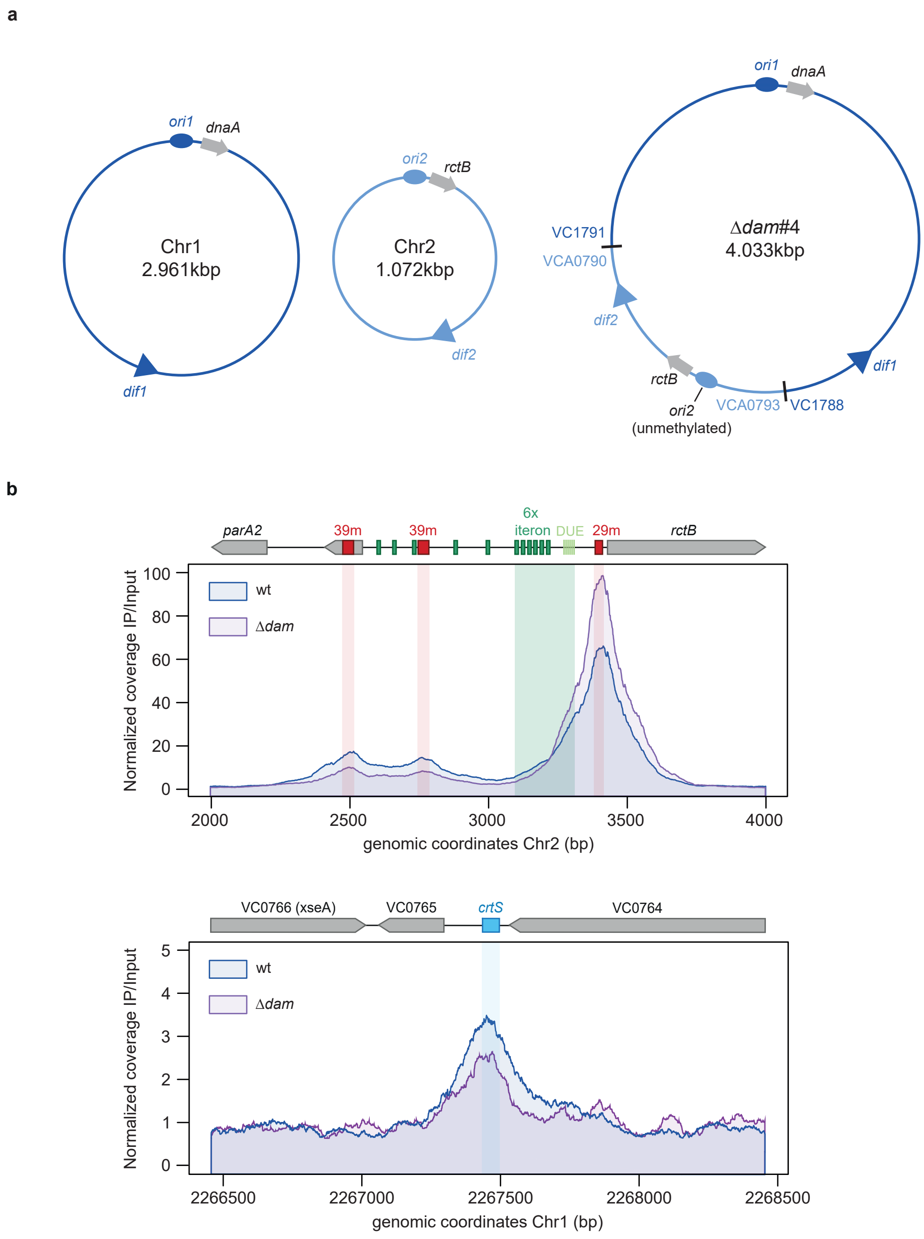

Supplementary Figure 5

Supplementary Figure 6: Darkfield TEM analysis of RctB nucleoprotein complexes formed with DNA fragments containing either the entire *ori2* region or truncated versions.

a. Top panel : TEM images (positive staining) of RctB bound to a 1793 bp DNA fragment containing the full *ori2* region, which includes three RctB binding sites: 39mL, 39mR, and 29m (representation to scale). Image **A** shows the control (DNA only). Images **B1-B16** depict various binding patterns of RctB across the 39m and 29m sites. **B1-B5**: RctB occupies all three sites (39mL, 39mR, and 29m), **B6-B8**: RctB occupies only the two 39m sites, **B9-B10**: RctB occupies only the 29m site, **B11-B16**: RctB occupies one 39m site and the 29m site. Bottom panel : Representative histogram of RctB binding frequency along the ori2 fragment (n=135). The x-axis represents the DNA divided into 60 segments, and the y-axis shows the frequency of RctB binding.

b. Top panel : TEM images of RctB bound to a truncated *ori2* DNA fragment (1555 bp) containing two RctB binding sites: 39mL and 39mR. Image **A** shows the control (DNA only). Images **B1-B8** illustrate various RctB binding patterns to the 39m sites. Bottom panel : Representative histogram of RctB binding frequency along the DNA fragment (n=127).

c. Top panel : TEM images of RctB bound to a truncated *ori2* DNA fragment (1617 bp) containing a single RctB binding site: 29m. Image **A** shows the control (DNA only). Images **B1-B2** show RctB bound to the 29m site. Bottom panel : Representative histogram of RctB binding frequency along the DNA fragment (n=180).

All TEM images were captured at the same magnification (X28980), with a scale bar of 100 nm. All DNA molecules were incubated with the same amount of RctB. DNA was not methylated.

a

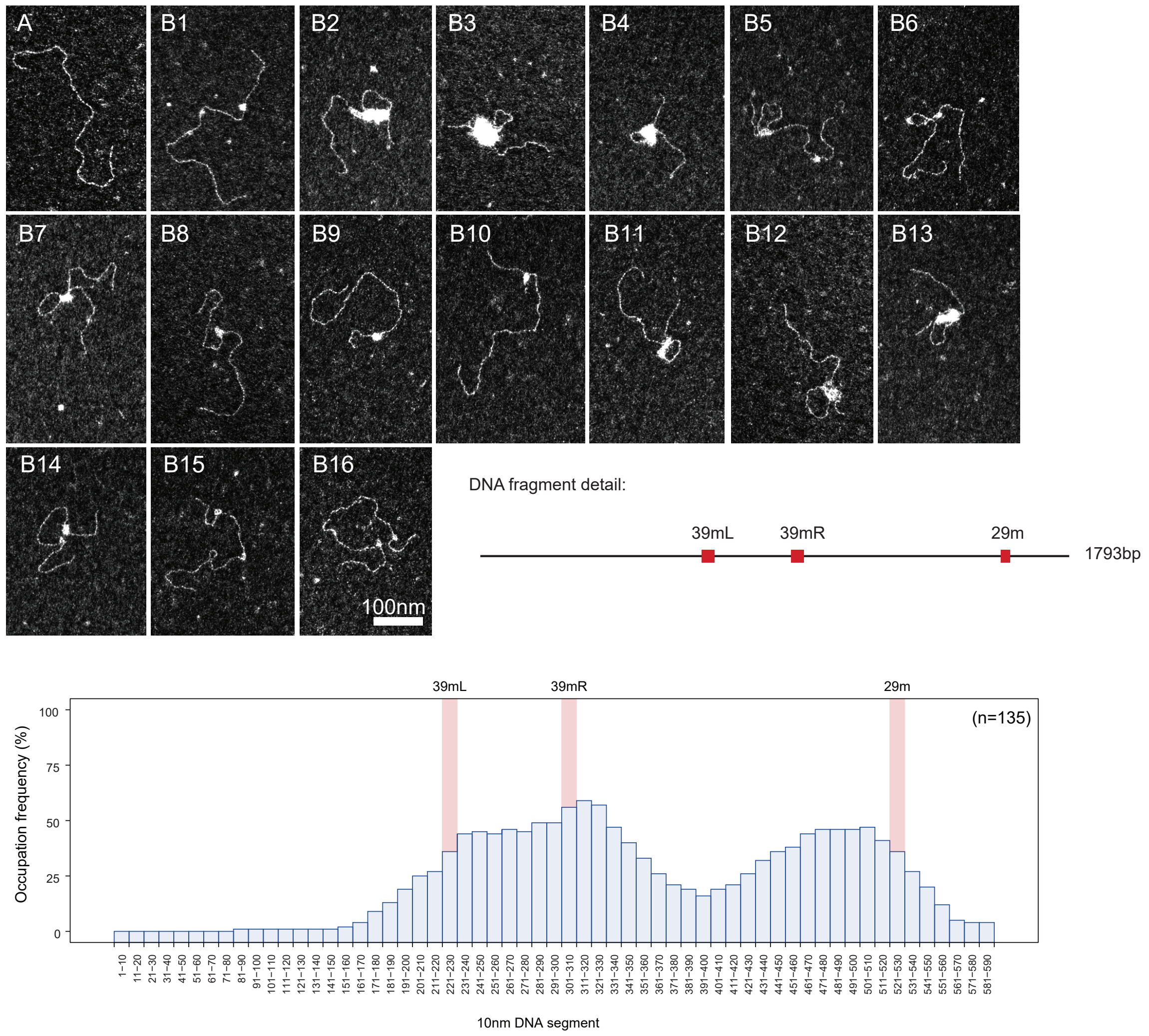

b

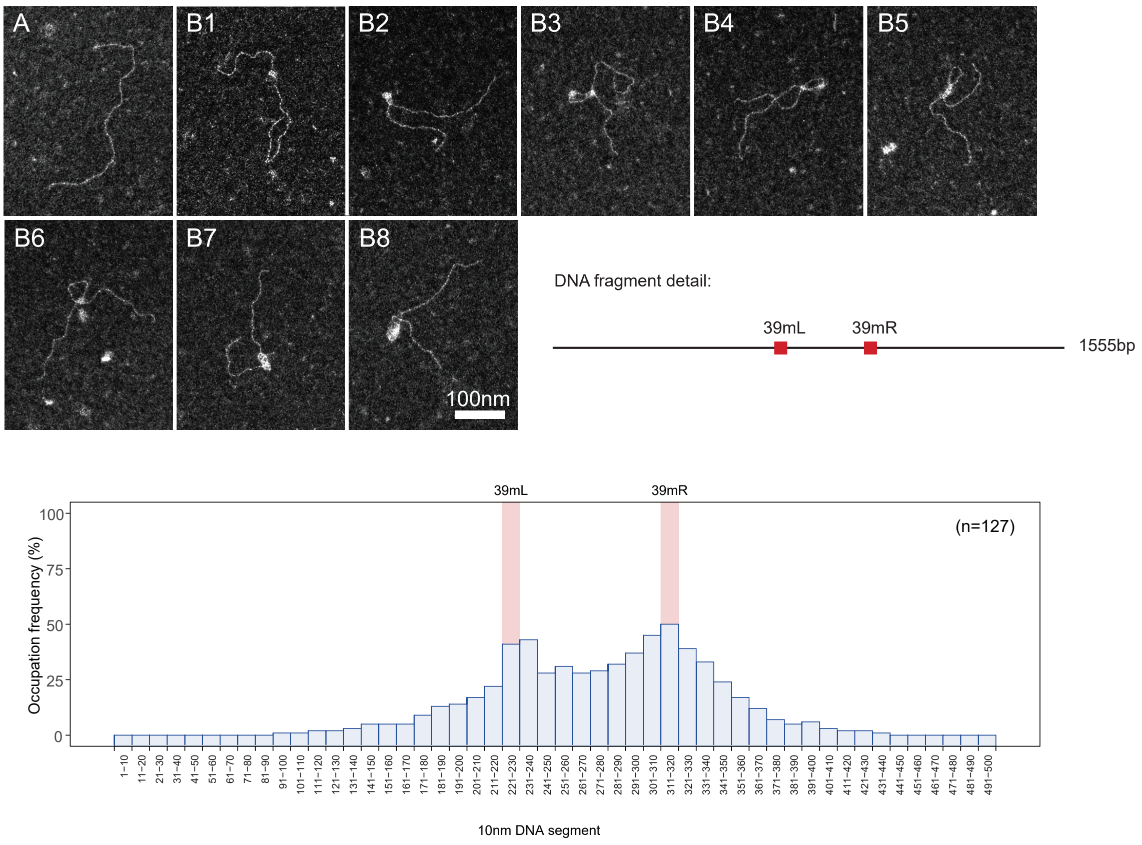

c

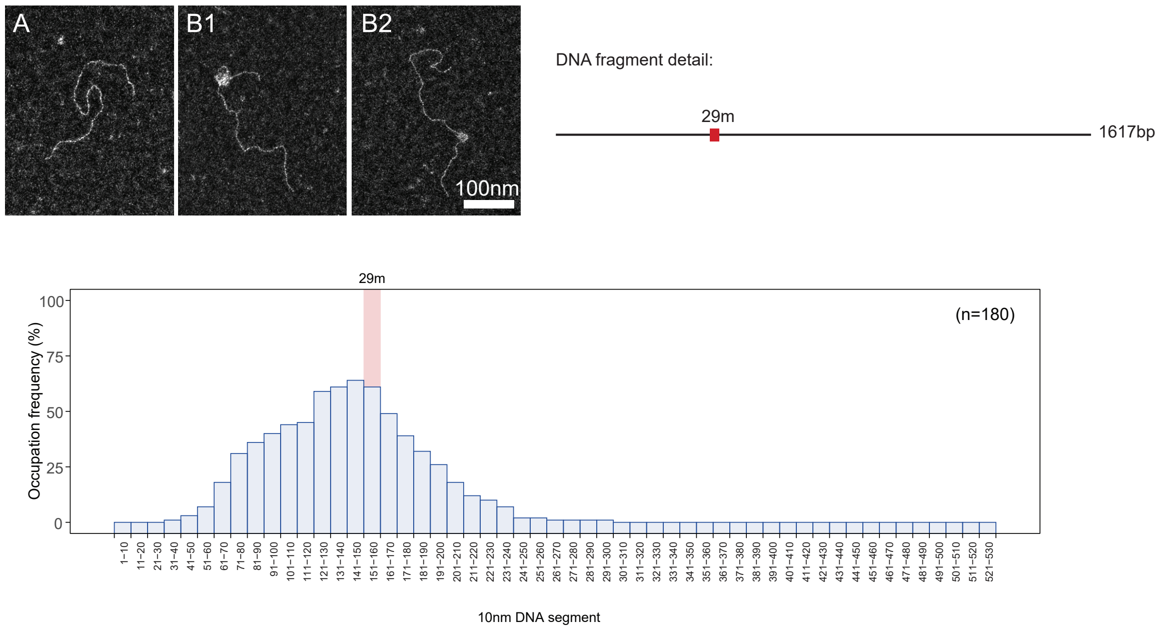

Supplementary Figure 6

Supplementary Figure 7: RctB specifically induces loops when bound to inhibitory sites at *ori2*

a. Schematic representation of the different DNA fragments containing one, two, or three inhibitory sites. Below each fragment, the potential interactions leading to loop formation are indicated by double-headed arrows.

b. Quantification of loop-containing molecules in the presence of RctB based on TEM images. The plot shows the percentage of molecules with loops, along with the number of molecules analyzed (indicated at the top of the plot). No binding: Represents the percentage of free DNA with no RctB bound. Binding: Represents the presence of RctB as indicated by punctual density on the DNA molecule. Looping: represents the presence of a DNA loop, where a loop is considered real only if there is increased density at its base, confirming it is mediated by RctB rather than a random DNA folding. Control DNA contains no RctB binding site.

a

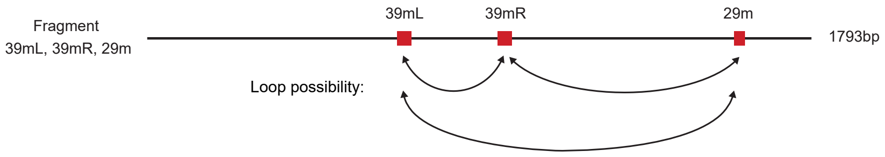

b

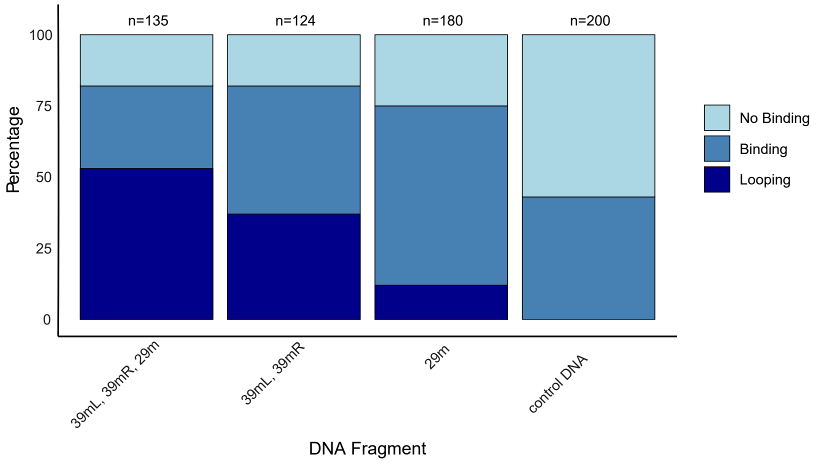

Supplementary Figure 7

Supplementary Figure 8: Size Exclusion Chromatography (SEC) of RctB Domain IV

a. 2Fo – Fc electron density map obtained from the structure factors and the phases calculated from the final model of RctB_DomIV. Map contour level 0.18 e/$Å^3$, and each chain of the homodimer colored in red and yellow, respectively.

b. The SEC profile of $RctB^{IV}$ was obtained on a Citiva Superdex 200 Increase 10/300. The chromatogram shows the signal intensity of $RctB^{IV}$ (blue curve), detected at a wavelength of 280 nm and measured in arbitrary units (a.u.) (left y-axis), plotted against the retention volume in mL (x-axis). The BioRad molecular weight standards (bovine thyroglobulin, 670 kDa, bovine γ-globuline, 158 kDa, chicken ovalbumin, 44 kDa, horse myoglobin, 17 kDa and vitamin B12, 1.35 kDa) were run under the same conditions, and their elution positions are indicated with black circles with the molecular weight in kDa, indicated on the right y-axis.

a

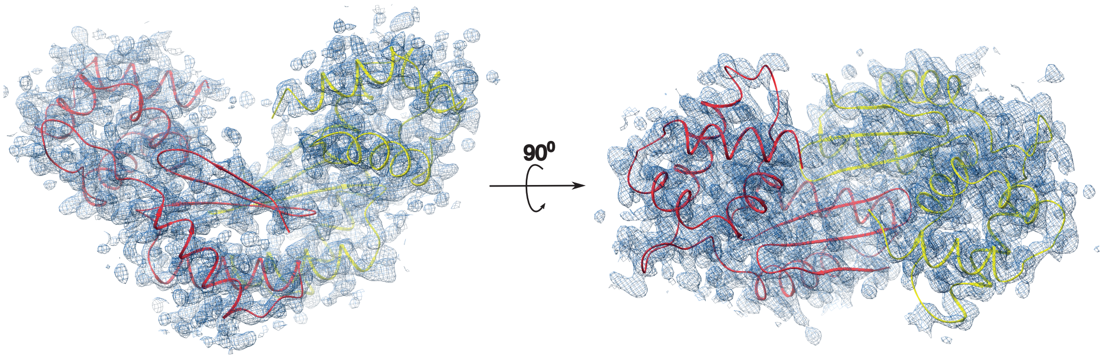

b

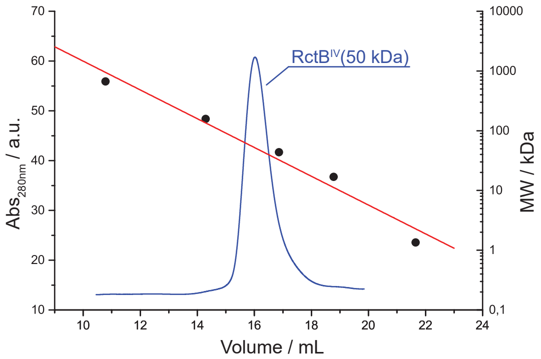

Supplementary Figure 8

Supplementary Figure 9: Characterization of the full-length RctB dimer using AlphaFold 3 and experimental validation

a. Crystal structures of the homodimer of RctB domains II and III (monomers are colored in light and dark blue, PBD 5UBF), and the homodimer of RctB domain IV (monomers are colored in pink and orange, reported in the present work).

b. Predicted AlphaFold 3 (AF3) model of the homodimer formed by the full length RctB (monomers colored in green and red). Each view has the same orientation of crystal structures shown in panel (a).

c. Superimposition of the crystal structures of panel (a) on the predicted model of panel (b).

d. SEC profile of RctB (red curve) obtained on a Citiva Superdex 200 Increase, together with the elution position of the molecular-weight standards (inverted triangles: bovine γ-globuline, 158 kDa; chicken ovalbumin, 44 kDa; equine myoglobin, 17 kDa; vitamin B12, 1.35 kDa). The calculated molecular weight of each RctB peak is indicated on chromatogram.

e. Coomassie stained SDS-PAGE gel of the RctB sample displaying one band under 100 kDa consistent with a single RctB polypeptide chain.

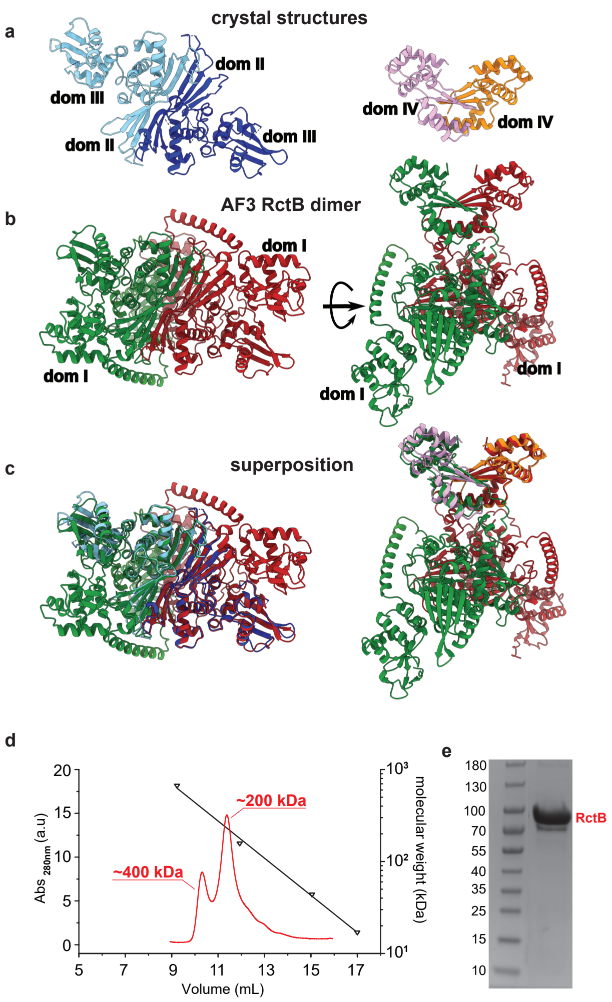

Supplementary Figure 9

Supplementary Figure 10: Effect of the deletion of *crtS* and *dam* on the binding of RctB-L_{651}P. Same legend as in Supplementary Fig.2. **a, b.** RctB ChIP signal is represented in blue for L651P mutant, green in the Δ*crtS* and purple in the Δ*dam* genetic background.

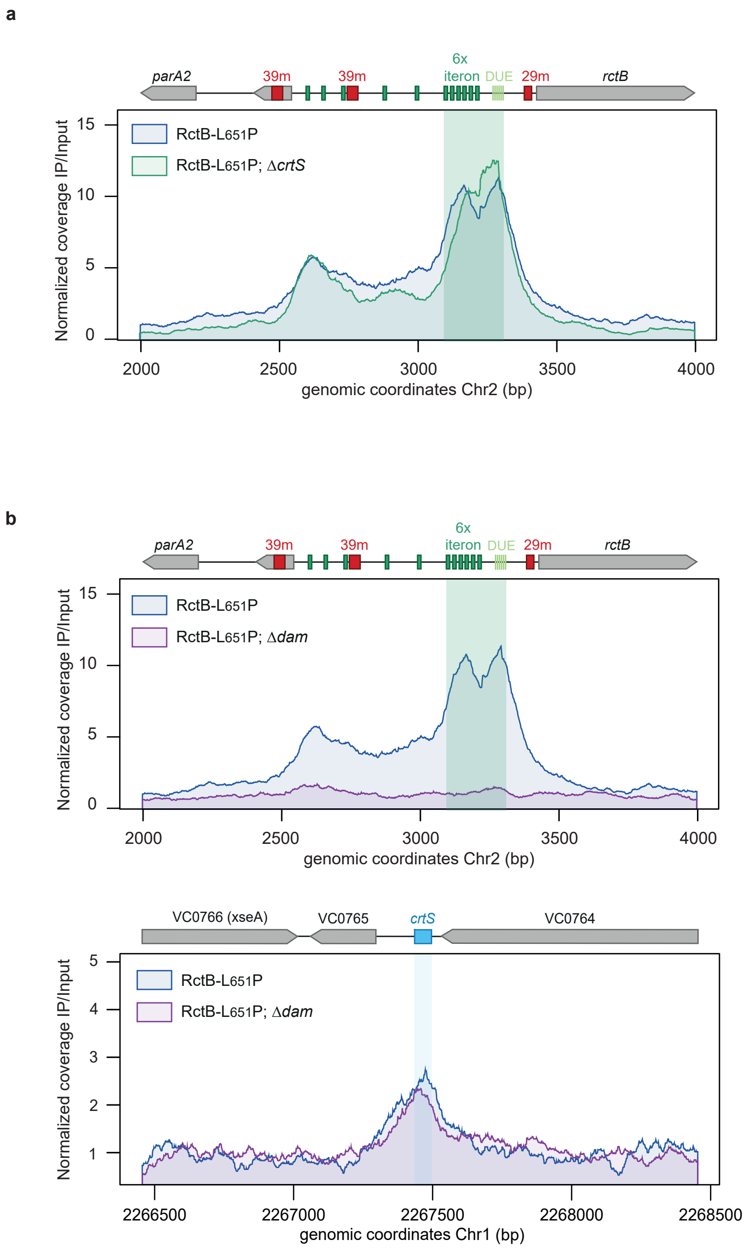

Supplementary Figure 10

Supplementary Figure 11: The effect of *crtS* on RctB binding is restricted to *ori2*

Barplot showing the ratio of the maximum height of RctB ChIP peaks along the genome in a Δ*crtS* strain relative to a wild-type (WT) strain. The ratio calculated from two independent ChIP experiments are shown as black dots, with bars representing the mean values. For *ori2*, the peak height shown corresponds to the peak on the 29mer site.

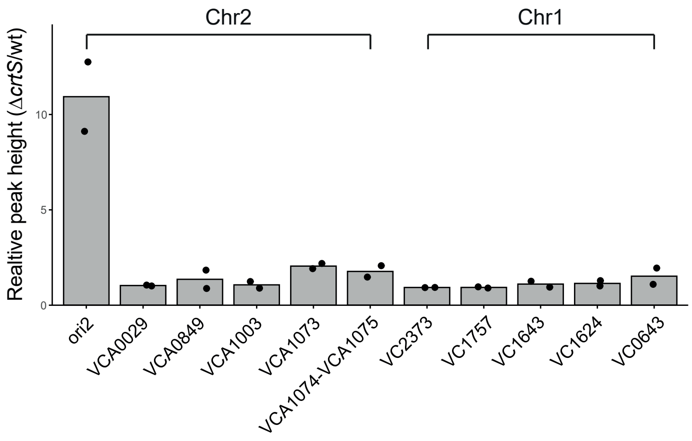

Supplementary Figure 11

Supplementary Figure 12: Darkfield TEM analysis of RctB nucleoprotein complexes formed with DNA fragments containing ori2 or crtS.

a. Top Panel : TEM observations of nucleoprotein complexes formed by RctB on a 944 bp DNA fragment containing an asymmetrically located *crtS* site (represented to scale). **A:** Control DNA only. **B1-B3**: TEM images showing RctB bound to the *crtS* site. RctB binding induces a kink in the DNA. Bottom panel : Histogram of RctB binding frequency along the *crtS* DNA fragment (n=204). The x-axis represents the DNA molecule divided into 31 segments.

b. TEM images of a mixture of DNA substrates containing *crtS* and *ori2* (represented to scale). Sporadic intermolecular contacts mediated by RctB bound to *crtS* and 29/39m sites are observed. White arrows indicate the nucleoprotein complexes.

All TEM images were captured at the same magnification (X28980), with a scale bar of 100 nm. All DNA molecules were incubated with the same amount of RctB. DNA substrates were not methylated.

a

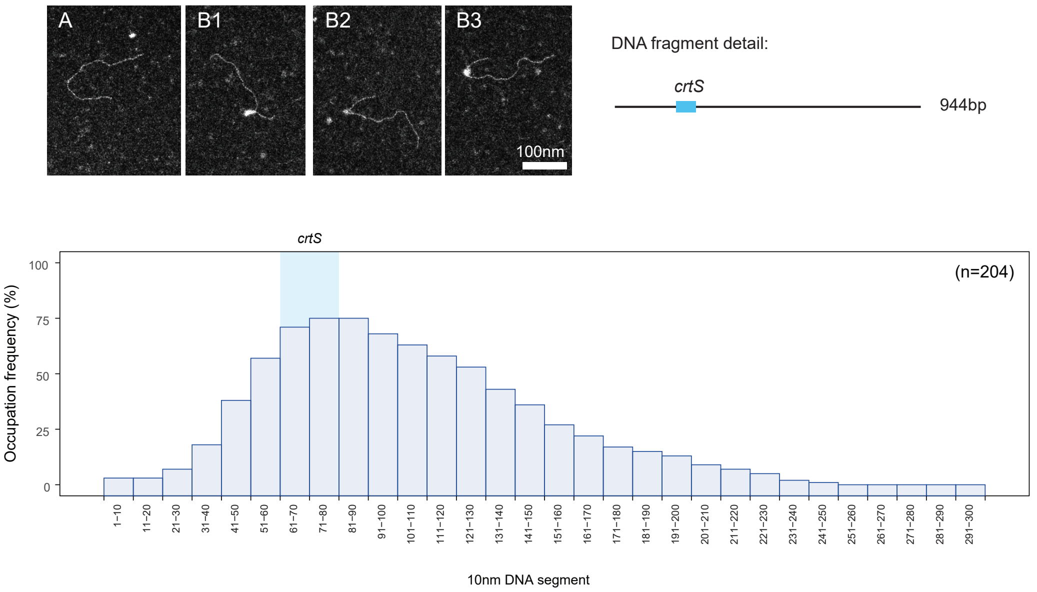

b

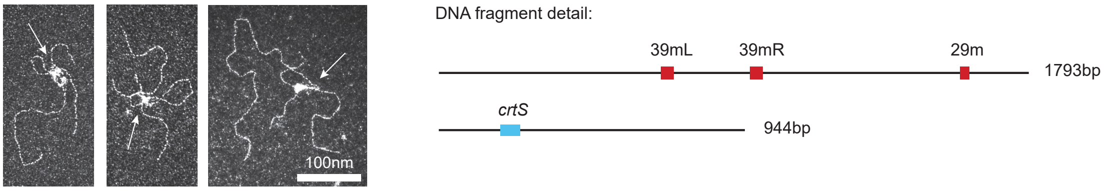

Supplementary Figure 12

Supplementary Figure 13. Two chromosomal copies of *crtS* have no influence on RctB binding at *ori2*.

a. Relative Chr2 copy number to Chr1 (*ori2*/*ori1*) measured by dPCR on gDNA of non-replicating *V. cholerae* (stationary phase), in strains with one chromosomal copy of *crtS* (Δ*crtS*; *attTn7*::*crtS*) or two chromosomal copies of *crtS* (Δ*crtS*; *attTn7*::2x*crtS*). The *attTn7* site is located near gene VC0487 on Chr1. The endogenous *crtS* site is deleted. Measurement were performed on at least 4 biological replicates per strain. **b.** ChIP-seq of RctB in V. cholerae Δ*crtS* mutants carrying either one or two copies of *crtS* in the *attTn7* site of Chr1. Legend same as Fig. 2a.

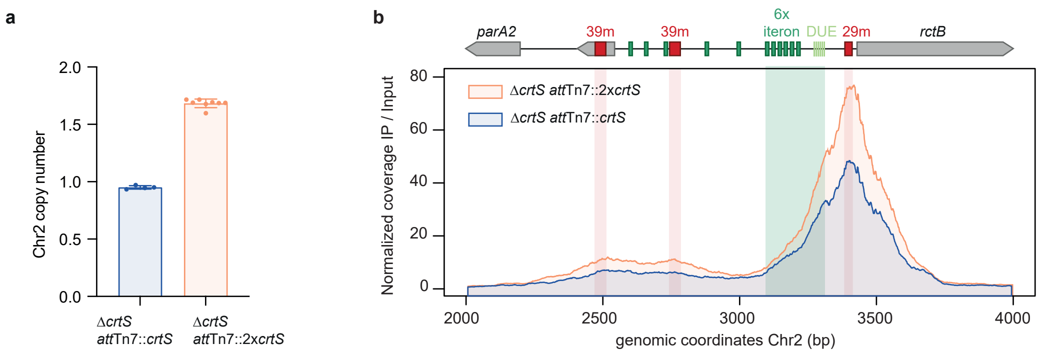

Supplementary Figure 13

Supplementary Figure 14. Live fluorescence microscopy of *V. cholerae* with one or two *crtS* copies

Live fluorescence microscopy in V. cholerae with 1 *crtS* at the native locus (**a**) and 2 *crtS* at the native locus and near *ori1* (**b**). Binding sites for fluorescent proteins were inserted near *ori1*, VC783 (near *crtS*) and *ori2*. The x-axis represents cell length (µm). The y-axis represents the longitudinal position of ori1, VC783, and ori2 foci within the cells relative to the old pole (0 being the old pole and 1 the new pole). The old pole of the cell was defined as the closest pole to one ori1 focus. Total number of cells analyzed n=1796 cells (wt), 1307 cells (2 *crtS*). p : percentage of cells.

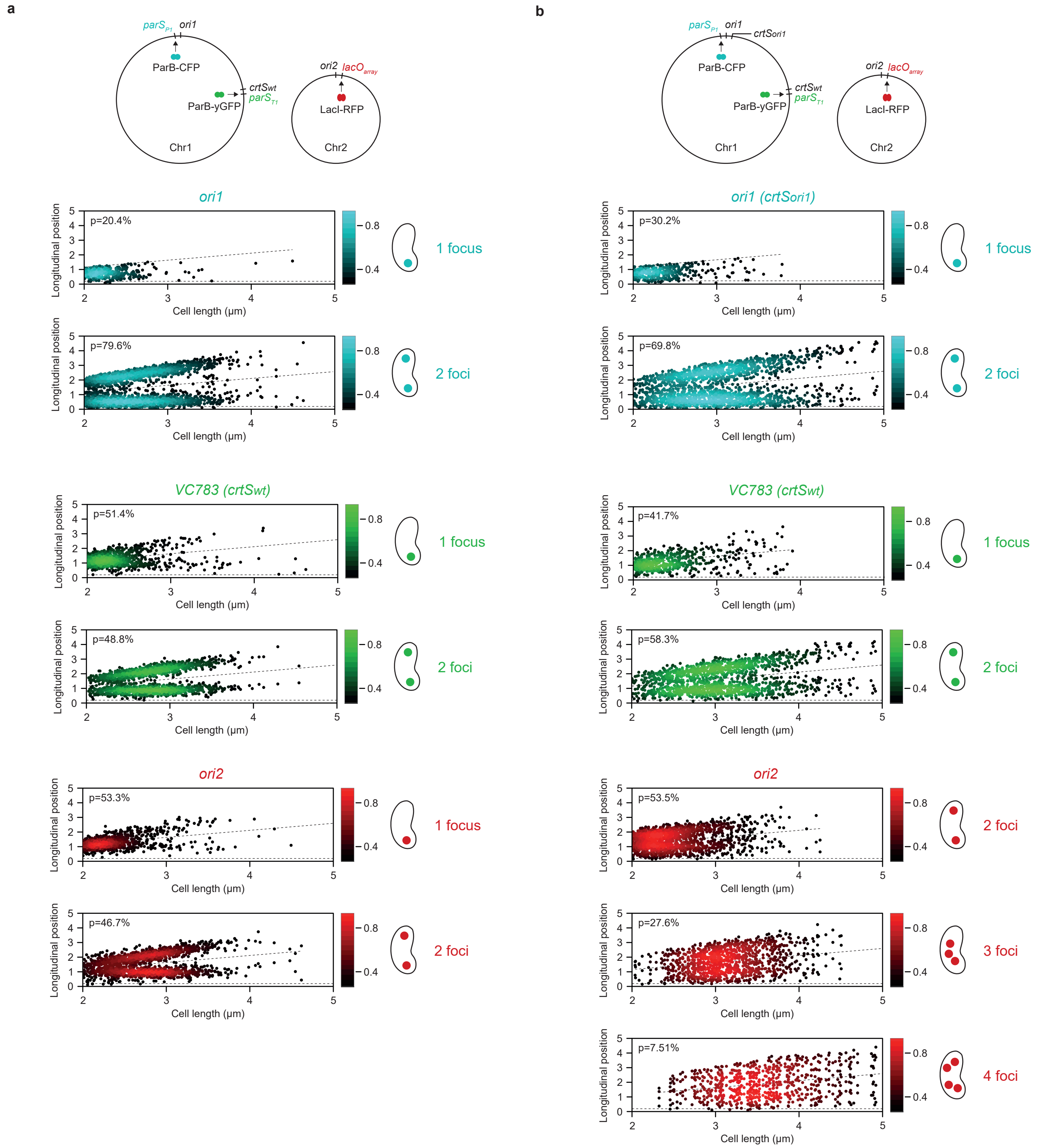

Supplementary Figure 14

Supplementary Figure 15. SHX Treatment halts cell growth in *V.cholerae* while allowing completion of ongoing replication round.

a. V. cholerae growth curve in M9 medium supplemented with 0.2% glucose and 0.4% casamino acids at 30°C. When cells reached OD600 = 0.5, DL-serine hydroxamate (SHX) was added to a final concentration of 1.5 mg/mL. The observed decrease in OD after SHX addition is due to a reduction in cell size, a well-documented phenomenon in *V. cholerae* under stringent response conditions [10]. After 180 minutes of SHX treatment, cells were washed and resuspended in fresh M9 medium to restart replication. **b.** Ratio of *ori1* to *ter1* measured by digital PCR (dPCR) at different time points during and after SHX treatment. During SHX treatment, the ori1/ter1 ratio decreases from 2 to 1, indicating the completion of ongoing replication. Following SHX removal, the ratio increases, marking the restart of replication.

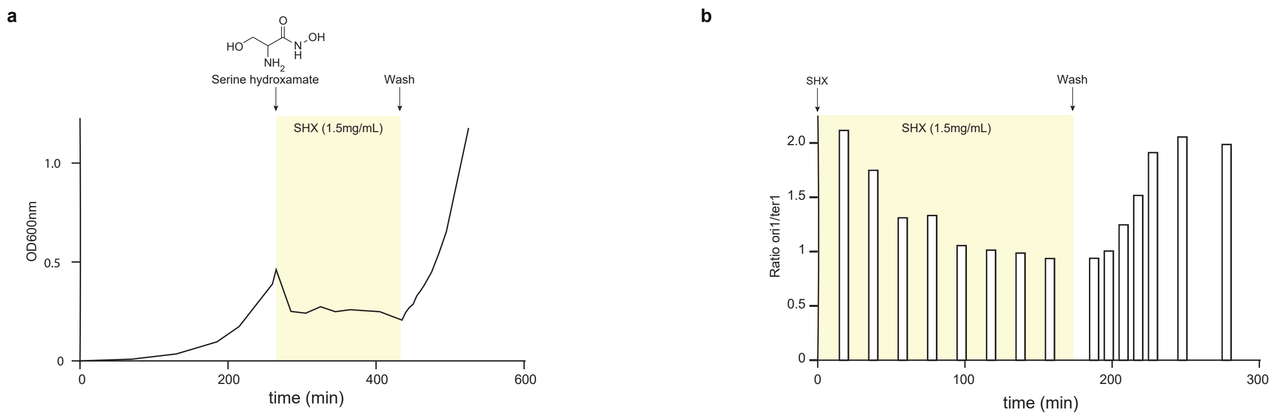

Supplementary Figure 15

Supplementary Figure 16: RctB binding pattern on *crtS* in synchronized population. ChIP-seq experiments on synchronized population at different timepoints after SHX removal (15min, 30min, 45min, 60min). On y axis: RctB normalized ChIP signal (IP coverage/INPUT coverage), on x axis: genomic coordinates on *V. cholerae* N16961 reference Chr1 (CP028827.1). A 2.000bp window is represented centered around *crtS*. Genetic is depicted on top of the graph (genes in grey arrows, *crtS* in light blue square).

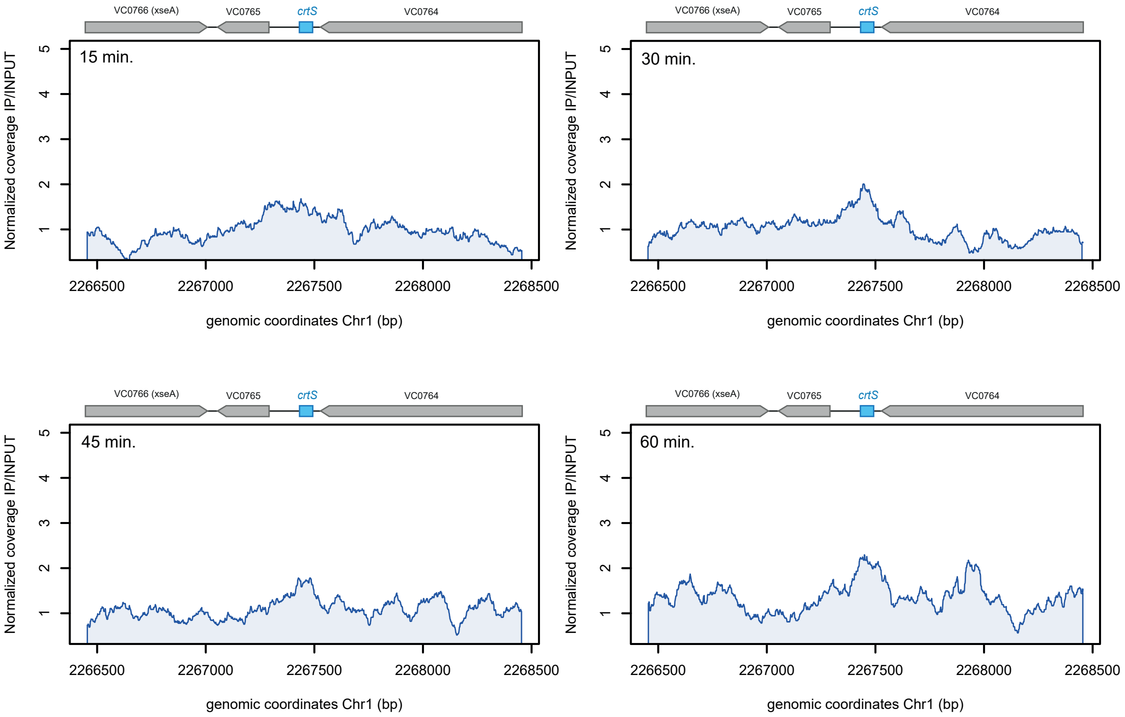

Supplementary Figure 16

Supplementary Figure 17: Marker frequency analysis on the input sample (not immunoprecipitated) from the ChIP experiment. Coverage is represented on y axis and genomic coordinates of both chromosomes (in kilo base pair) are present on the x axis. Coverage has been smoothed with a 10kb sliding window along the genome. Linear regression analysis was performed separately on each half of the chromosomes, and the best-fit lines were superimposed on the plot. The top section (in blue) shows MFA data for wt, ΔcrtS, and ΔcrtS-29mC>A (evolved strain). The bottom section (in green) displays MFA data for strains with RctB-L651P and RctB-L651P-ΔcrtS.

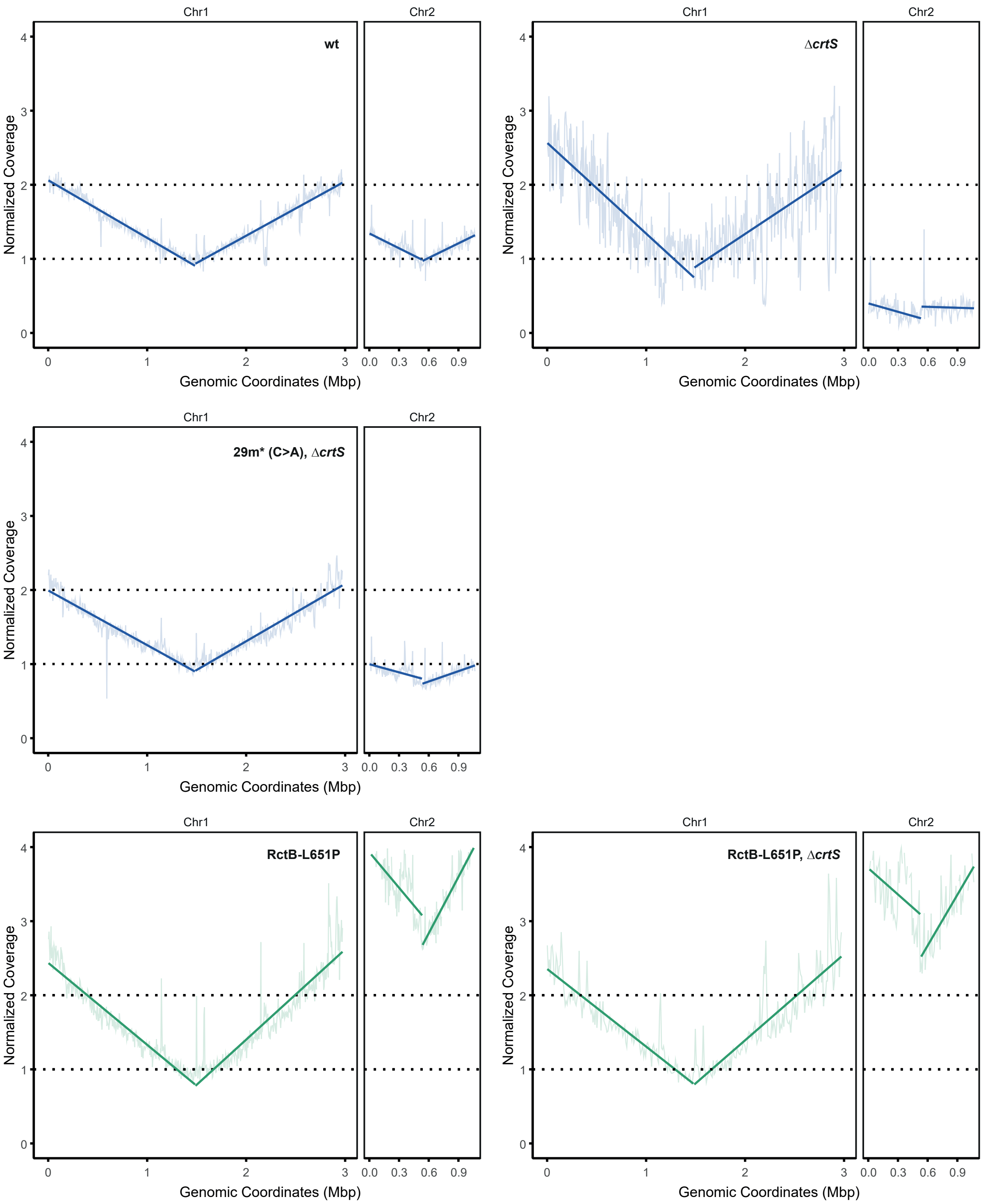

Supplementary Figure 17

SUPPLEMENTARY REFERENCES

1 de Lemos Martins, F., Fournes, F., Mazzuoli, M. V., Mazel, D. & Val, M. E. Vibrio cholerae chromosome 2 copy number is controlled by the methylation-independent binding of its monomeric initiator to the chromosome 1 crtS site. *Nucleic acids research* **46**, 10145-10156, doi:10.1093/nar/gky790 (2018).

2 Karimova, G., Pidoux, J., Ullmann, A. & Ladant, D. A bacterial two-hybrid system based on a reconstituted signal transduction pathway. *Proceedings of the National Academy of Sciences of the United States of America* **95**, 5752-5756, doi:10.1073/pnas.95.10.5752 (1998).

3 Fournes, F. *et al.* The coordinated replication of Vibrio cholerae's two chromosomes required the acquisition of a unique domain by the RctB initiator. *Nucleic acids research* **49**, 11119-11133, doi:10.1093/nar/gkab903 (2021).

4 Val, M. E., Skovgaard, O., Ducos-Galand, M., Bland, M. J. & Mazel, D. Genome engineering in Vibrio cholerae: a feasible approach to address biological issues. *PLoS genetics* **8**, e1002472, doi:10.1371/journal.pgen.1002472 (2012).

5 Le Roux, F., Binesse, J., Saulnier, D. & Mazel, D. Construction of a Vibrio splendidus mutant lacking the metalloprotease gene vsm by use of a novel counterselectable suicide vector. *Applied and environmental microbiology* **73**, 777-784, doi:10.1128/AEM.02147-06 (2007).

6 Kuhn, J. *et al.* Glucose- but not rice-based oral rehydration therapy enhances the production of virulence determinants in the human pathogen Vibrio cholerae. *PLoS neglected tropical diseases* **8**, e3347, doi:10.1371/journal.pntd.0003347 (2014).

7 Val, M. E. *et al.* Fuse or die: how to survive the loss of Dam in Vibrio cholerae. *Molecular microbiology* **91**, 665-678, doi:10.1111/mmi.12483 (2014).

8 Val, M. E. *et al.* A checkpoint control orchestrates the replication of the two chromosomes of Vibrio cholerae. *Science advances* **2**, e1501914, doi:10.1126/sciadv.1501914 (2016).

9 Yamaichi, Y., Fogel, M. A., McLeod, S. M., Hui, M. P. & Waldor, M. K. Distinct centromere-like parS sites on the two chromosomes of Vibrio spp. *Journal of bacteriology* **189**, 5314-5324, doi:10.1128/JB.00416-07 (2007).

10 Kemter, F. S. *et al.* Stringent response leads to continued cell division and a temporal restart of DNA replication after initial shutdown in Vibrio cholerae. *Molecular microbiology* **111**, 1617-1637, doi:10.1111/mmi.14241 (2019).
