# Supplementary material for: Dynamic transitions of initiator binding coordinate the replication of the two chromosomes in Vibrio cholerae
Source: Nat Commun. 2025 Jan 8;16:485. doi: 10.1038/s41467-024-55598-9 (PMC11711613; doi:10.1038/s41467-024-55598-9)
Supplement: Supplementary file 2 — Reporting Summary [file 41467_2024_55598_MOESM2_ESM.pdf]

nature portfolio

Corresponding author(s): VAL Marie-Eve
MAZEL Didier
GARCIA PINO Abel

Last updated by author(s): Dec 4, 2024

Reporting Summary

Nature Portfolio wishes to improve the reproducibility of the work that we publish. This form provides structure for consistency and transparency in reporting. For further information on Nature Portfolio policies, see our Editorial Policies and the Editorial Policy Checklist.

Statistics

For all statistical analyses, confirm that the following items are present in the figure legend, table legend, main text, or Methods section.

n/a	Confirmed	
☐	☒	The exact sample size (*n*) for each experimental group/condition, given as a discrete number and unit of measurement
☐	☒	A statement on whether measurements were taken from distinct samples or whether the same sample was measured repeatedly
☐	☒	The statistical test(s) used AND whether they are one- or two-sided<br>*Only common tests should be described solely by name; describe more complex techniques in the Methods section.*
☒	☐	A description of all covariates tested
☒	☐	A description of any assumptions or corrections, such as tests of normality and adjustment for multiple comparisons
☐	☒	A full description of the statistical parameters including central tendency (e.g. means) or other basic estimates (e.g. regression coefficient) AND variation (e.g. standard deviation) or associated estimates of uncertainty (e.g. confidence intervals)
☐	☒	For null hypothesis testing, the test statistic (e.g. *F*, *t*, *r*) with confidence intervals, effect sizes, degrees of freedom and *P* value noted<br>*Give P values as exact values whenever suitable.*
☒	☐	For Bayesian analysis, information on the choice of priors and Markov chain Monte Carlo settings
☒	☐	For hierarchical and complex designs, identification of the appropriate level for tests and full reporting of outcomes
☒	☐	Estimates of effect sizes (e.g. Cohen's *d*, Pearson's *r*), indicating how they were calculated

Our web collection on statistics for biologists contains articles on many of the points above.

Software and code

Policy information about availability of computer code

Data collection	*Provide a description of all commercial, open source and custom code used to collect the data in this study, specifying the version used OR state that no software was used.*
Data analysis	The workflow for processing Illumina deep sequencing ChIPseq data (including custom code) is provided in Supplementary Information

For manuscripts utilizing custom algorithms or software that are central to the research but not yet described in published literature, software must be made available to editors and reviewers. We strongly encourage code deposition in a community repository (e.g. GitHub). See the Nature Portfolio guidelines for submitting code & software for further information.

Data

Policy information about availability of data

All manuscripts must include a data availability statement. This statement should provide the following information, where applicable:

- Accession codes, unique identifiers, or web links for publicly available datasets
- A description of any restrictions on data availability
- For clinical datasets or third party data, please ensure that the statement adheres to our policy

The sequencing data generated in this study have been deposited in the European Nucleotide Archive under project accession code PRJEB63079 (https://www.ebi.ac.uk/ena/browser/view/PRJEB63079). A detailed list of accession codes is available in the Supplementary Information. The structural data for RctB domain IV have been deposited in the Protein Data Bank (PDB) under entry 8RV3. All additional data generated in this study are provided in the Source Data file.

Research involving human participants, their data, or biological material

Policy information about studies with human participants or human data. See also policy information about sex, gender (identity/presentation), and sexual orientation and race, ethnicity and racism.

Reporting on sex and gender	*Use the terms sex (biological attribute) and gender (shaped by social and cultural circumstances) carefully in order to avoid confusing both terms. Indicate if findings apply to only one sex or gender; describe whether sex and gender were considered in study design; whether sex and/or gender was determined based on self-reporting or assigned and methods used.*<br>*Provide in the source data disaggregated sex and gender data, where this information has been collected, and if consent has been obtained for sharing of individual-level data; provide overall numbers in this Reporting Summary. Please state if this information has not been collected.*<br>*Report sex- and gender-based analyses where performed, justify reasons for lack of sex- and gender-based analysis.*
Reporting on race, ethnicity, or other socially relevant groupings	*Please specify the socially constructed or socially relevant categorization variable(s) used in your manuscript and explain why they were used. Please note that such variables should not be used as proxies for other socially constructed/relevant variables (for example, race or ethnicity should not be used as a proxy for socioeconomic status).*<br>*Provide clear definitions of the relevant terms used, how they were provided (by the participants/respondents, the researchers, or third parties), and the method(s) used to classify people into the different categories (e.g. self-report, census or administrative data, social media data, etc.)*<br>*Please provide details about how you controlled for confounding variables in your analyses.*
Population characteristics	*Describe the covariate-relevant population characteristics of the human research participants (e.g. age, genotypic information, past and current diagnosis and treatment categories). If you filled out the behavioural & social sciences study design questions and have nothing to add here, write "See above."*
Recruitment	*Describe how participants were recruited. Outline any potential self-selection bias or other biases that may be present and how these are likely to impact results.*
Ethics oversight	*Identify the organization(s) that approved the study protocol.*

Note that full information on the approval of the study protocol must also be provided in the manuscript.

Field-specific reporting

Please select the one below that is the best fit for your research. If you are not sure, read the appropriate sections before making your selection.

☒ Life sciences ☐ Behavioural & social sciences ☐ Ecological, evolutionary & environmental sciences

For a reference copy of the document with all sections, see nature.com/documents/nr-reporting-summary-flat.pdf

Life sciences study design

All studies must disclose on these points even when the disclosure is negative.

Sample size	For microscopy, more than 1000 cells were analyzed.
Data exclusions	No data were excluded for the analysis
Replication	Biological replicates
Randomization	Not relevant
Blinding	Not relevant

Reporting for specific materials, systems and methods

We require information from authors about some types of materials, experimental systems and methods used in many studies. Here, indicate whether each material, system or method listed is relevant to your study. If you are not sure if a list item applies to your research, read the appropriate section before selecting a response.

Materials & experimental systems

n/a	Involved in the study
[ ]	[x] Antibodies
[x]	[ ] Eukaryotic cell lines
[x]	[ ] Palaeontology and archaeology
[x]	[ ] Animals and other organisms
[x]	[ ] Clinical data
[x]	[ ] Dual use research of concern
[x]	[ ] Plants

Methods

n/a	Involved in the study
[ ]	[x] ChIP-seq
[ ]	[ ] Flow cytometry
[ ]	[ ] MRI-based neuroimaging

Antibodies

Antibodies used: anti-FLAG antibodies (F7425 Sigma)

Validation: The specificity of anti-FLAG commercial antibodies was confirmed using negative controls (strains lacking FLAG epitope).

Plants

Seed stocks: *Report on the source of all seed stocks or other plant material used. If applicable, state the seed stock centre and catalogue number. If plant specimens were collected from the field, describe the collection location, date and sampling procedures.*

Novel plant genotypes: *Describe the methods by which all novel plant genotypes were produced. This includes those generated by transgenic approaches, gene editing, chemical/radiation-based mutagenesis and hybridization. For transgenic lines, describe the transformation method, the number of independent lines analyzed and the generation upon which experiments were performed. For gene-edited lines, describe the editor used, the endogenous sequence targeted for editing, the targeting guide RNA sequence (if applicable) and how the editor was applied.*

Authentication: *Describe any authentication procedures for each seed stock used or novel genotype generated. Describe any experiments used to assess the effect of a mutation and, where applicable, how potential secondary effects (e.g. second site T-DNA insertions, mosiacism, off-target gene editing) were examined.*

ChIP-seq

Data deposition

[x] Confirm that both raw and final processed data have been deposited in a public database such as GEO.

[ ] Confirm that you have deposited or provided access to graph files (e.g. BED files) for the called peaks.

Data access links
May remain private before publication.
The sequencing data generated in this study have been deposited in the European Nucleotide Archive under project accession code PRJEB63079 (https://www.ebi.ac.uk/ena/browser/view/PRJEB63079).

Files in database submission: For ChIPseq negative control (no FLAG tag): ERR12420106. For RctB ChIP-seq in exponential phase : ERR12420290 and ERR12651384 (IP) - ERR12420289 and ERR12651383 (INPUT). For RctB ChIP-seq in stationary phase: ERR12420287 and ERR12651392 (IP) and ERR12420288 and ERR12651391 (INPUT). For RctB ChIP-seq in 29m C>A mutated context: ERR12420285 and ERR12651382 (IP) - ERR12420286 and ERR12651381 (INPUT). For ParB2 ChIP-seq: ERR12420283 and ERR12651380 (IP) - ERR12420284 and ERR12651379 (INPUT). For RctB-L651P ChIP-seq in wt context: ERR12420292 and ERR12651386 (IP) - ERR12420291 and ERR12651385 (INPUT). For RctB ChIP-seq in ΔcrtS context: ERR12421767 and ERR12651396 (IP) -ERR12421766 and ERR12651395 (INPUT). For RctB-L651P ChIP-seq in ΔcrtS context: ERR12492377 and ERR12651390 (IP) - ERR12492376 and ERR12651389 (INPUT). For RctB ChIP-seq in a context with crtS relocated to attTN7 site: ERR12421765 (IP) and ERR12421764 (INPUT). For RctB ChIP-seq in a context with two crtS sites inserted in attTN7 site, ERR12421763 (IP) and ERR12421762 (INPUT). For RctB ChIP-seq in Δdam context: ERR12493891 and ERR12651394 (IP) - ERR12493890 and ERR12651393 (INPUT). For RctB-L651P ChIP-seq in Δdam context ERR12493889 and ERR12651388 (IP) and ERR12493888 and ERR12651387 (INPUT). For RctB ChIP-seq in synchronized population: 15min after release ERR12421789 and ERR13771528 (IP) - ERR12421788 and ERR13771529 (INPUT), 30min after release ERR12421791 and ERR13771530 (IP) and ERR12421790 and ERR13771531 (INPUT), 45min after release ERR12421793 and ERR13771532 (IP) - ERR12421792 and ERR13771533 (INPUT), 60min after release ERR12421795 and ERR13771534 (IP) - ERR12421794 and ERR13771535 (INPUT). For MFA analysis: wt strain ERR12492335, mutant strain with crtS near ori1 ERR12492336 and mutant with two crtS sites at wt and at ori1 ERR12492337.

Genome browser session
(e.g. UCSC)
no longer applicable

Methodology

Replicates: At least two biological replicates

Sequencing depth	Sequencing depth ranges from 50 to 200 at each position.
Antibodies	anti-FLAG magnetic beads (M8823 Sigma)
Peak calling parameters	We conducted peak calling using MACS2 with standard settings, including --keep-dup 1 to handle duplicate reads, --bdg for generating bedGraph files, --qvalue 0.05 to control the false discovery rate, --mfold 5 50 to detect enriched regions with at least a 5-fold change and a 50-bp minimum length, and --bw 300 for adjusting bandwidth.
Data quality	To ensure the reliability of our data, we assessed sequencing quality using the FastQC program and evaluated peak quality using the MACS2 program, both of which are widely recognized tools for quality control in ChIPseq analysis.
Software	We used a custom R script to process the data and generate informative graphs, the script is included in Supplementary information.

Flow Cytometry

Plots

Confirm that:

☐ The axis labels state the marker and fluorochrome used (e.g. CD4-FITC).

☐ The axis scales are clearly visible. Include numbers along axes only for bottom left plot of group (a 'group' is an analysis of identical markers).

☐ All plots are contour plots with outliers or pseudocolor plots.

☐ A numerical value for number of cells or percentage (with statistics) is provided.

Methodology

Sample preparation	*Describe the sample preparation, detailing the biological source of the cells and any tissue processing steps used.*
Instrument	*Identify the instrument used for data collection, specifying make and model number.*
Software	*Describe the software used to collect and analyze the flow cytometry data. For custom code that has been deposited into a community repository, provide accession details.*
Cell population abundance	*Describe the abundance of the relevant cell populations within post-sort fractions, providing details on the purity of the samples and how it was determined.*
Gating strategy	*Describe the gating strategy used for all relevant experiments, specifying the preliminary FSC/SSC gates of the starting cell population, indicating where boundaries between "positive" and "negative" staining cell populations are defined.*

☐ Tick this box to confirm that a figure exemplifying the gating strategy is provided in the Supplementary Information.

Magnetic resonance imaging

Experimental design

Design type	*Indicate task or resting state; event-related or block design.*
Design specifications	*Specify the number of blocks, trials or experimental units per session and/or subject, and specify the length of each trial or block (if trials are blocked) and interval between trials.*
Behavioral performance measures	*State number and/or type of variables recorded (e.g. correct button press, response time) and what statistics were used to establish that the subjects were performing the task as expected (e.g. mean, range, and/or standard deviation across subjects).*

Acquisition

Imaging type(s)	*Specify: functional, structural, diffusion, perfusion.*
Field strength	*Specify in Tesla*
Sequence & imaging parameters	*Specify the pulse sequence type (gradient echo, spin echo, etc.), imaging type (EPI, spiral, etc.), field of view, matrix size, slice thickness, orientation and TE/TR/flip angle.*
Area of acquisition	*State whether a whole brain scan was used OR define the area of acquisition, describing how the region was determined.*

Diffusion MRI ☐ Used ☐ Not used

Preprocessing

Preprocessing software	*Provide detail on software version and revision number and on specific parameters (model/functions, brain extraction, segmentation, smoothing kernel size, etc.).*
Normalization	*If data were normalized/standardized, describe the approach(es): specify linear or non-linear and define image types used for transformation OR indicate that data were not normalized and explain rationale for lack of normalization.*
Normalization template	*Describe the template used for normalization/transformation, specifying subject space or group standardized space (e.g. original Talairach, MNI305, ICBM152) OR indicate that the data were not normalized.*
Noise and artifact removal	*Describe your procedure(s) for artifact and structured noise removal, specifying motion parameters, tissue signals and physiological signals (heart rate, respiration).*
Volume censoring	*Define your software and/or method and criteria for volume censoring, and state the extent of such censoring.*

Statistical modeling & inference

Model type and settings	*Specify type (mass univariate, multivariate, RSA, predictive, etc.) and describe essential details of the model at the first and second levels (e.g. fixed, random or mixed effects; drift or auto-correlation).*
Effect(s) tested	*Define precise effect in terms of the task or stimulus conditions instead of psychological concepts and indicate whether ANOVA or factorial designs were used.*

Specify type of analysis: ☐ Whole brain ☐ ROI-based ☐ Both

Statistic type for inference (See Eklund et al. 2016)	*Specify voxel-wise or cluster-wise and report all relevant parameters for cluster-wise methods.*
Correction	*Describe the type of correction and how it is obtained for multiple comparisons (e.g. FWE, FDR, permutation or Monte Carlo).*

Models & analysis

n/a	Involved in the study
☐	☐ Functional and/or effective connectivity
☐	☐ Graph analysis
☐	☐ Multivariate modeling or predictive analysis

Functional and/or effective connectivity	*Report the measures of dependence used and the model details (e.g. Pearson correlation, partial correlation, mutual information).*
Graph analysis	*Report the dependent variable and connectivity measure, specifying weighted graph or binarized graph, subject- or group-level, and the global and/or node summaries used (e.g. clustering coefficient, efficiency, etc.).*
Multivariate modeling and predictive analysis	*Specify independent variables, features extraction and dimension reduction, model, training and evaluation metrics.*
