# Supplementary material for: Dynamic transitions of initiator binding coordinate the replication of the two chromosomes in Vibrio cholerae
Source: Nat Commun. 2025 Jan 8;16:485. doi: 10.1038/s41467-024-55598-9 (PMC11711613; doi:10.1038/s41467-024-55598-9)
Supplement: Supplementary file 3 — Description of Additional Supplementary Files [file 41467_2024_55598_MOESM3_ESM.pdf]

Description of Additional Supplementary Files

Supplementary Data 1: RctB binding sites identified from ChIP-seq experiment. ChIP reads data were aligned on Vibrio cholerae N16961 reference genome (CP028827.1 for Chr1 and CP028828.1 for Chr2). Peak summit, p-value and fold enrichment are calculated with MACS2.0 program by comparing RctB-FLAG versus wt (no FLAG) ChIP-seq data. ChIP peaks motif are found using the MEME suite, two sequences are significantly enriched and correspond to already known iterons and 39m sites. N /S = No similarity with known sequences.

Supplementary Movie 1: Animation depicting replication dynamics interpreted from the MFA data shown in Figure 4e (Left Panel: wild type).

Supplementary Movie 2: Animation depicting replication dynamics interpreted from the MFA data shown in Figure 4e (Middle Panel: mutant with relocated crtS near ori1).

Supplementary Movie 3: Animation depicting replication dynamics interpreted from the MFA data shown in Figure 4e (Right Panel: mutant with two crtS sites).

Supplementary Software: Workflow for processing Illumina deep sequencing ChIP-seq data
