# Supplementary material for: Dynamic transitions of initiator binding coordinate the replication of the two chromosomes in Vibrio cholerae
Source: Nat Commun. 2025 Jan 8;16:485. doi: 10.1038/s41467-024-55598-9 (PMC11711613; doi:10.1038/s41467-024-55598-9)
Supplement: Supplementary file 8 — Supplementary Software [file 41467_2024_55598_MOESM8_ESM.zip › NCOMMS-24-05351B Supplementary Software/482965_2_data_set_10036673_s4bq1b/Supplementary Software/ChIP-seq analysis Workflow.docx]

**Workflow for processing Illumina deep sequencing ChIP-seq data:**

Step 1: mapping reads to reference genome

1. Input: Raw reads from Illumina deep sequencing.

2. Mapping: use Bowtie with standard parameters to map reads onto the Vibrio cholerae N16961 reference genome.

3. Generate coverage data: Use samtools depth to calculate coverage from the mapped reads. Use the option Output all positions (including those with zero depth) (-a).

Step 2: Adjusting Total Coverage

This step adjusts the total coverage from the INPUT file to match the total coverage of the IP file.

In R:

# Import INPUT file (coverage for the control sample without immunoprecipitation)

input_file <- file_INPUT

colnames(input_file) <- c("chrom", "pos", "cov") # Assign column names

total_cov_input <- sum(input_file$cov) # Calculate total coverage for the INPUT file

# Import IP file (coverage for the sample with immunoprecipitation)

ip_file <- file_IP

colnames(ip_file) <- c("chrom", "pos", "cov") # Assign column names

total_cov_ip <- sum(ip_file$cov) # Calculate total coverage for the IP file

# Adjust INPUT file coverage to match IP file coverage

adjustment_factor <- total_cov_ip / total_cov_input

adjusted_cov <- input_file$cov * adjustment_factor

# Create a new data frame with adjusted coverage

adjusted_input <- data.frame(pos = input_file$pos, cov = adjusted_cov)

# Save the adjusted coverage data to a CSV file

output_file <- "file_INPUT_adjusted.csv"

write.csv(adjusted_input, file = output_file, row.names = FALSE)

Step 3: Normalizing IP Coverage Using a Local Median Window

This script normalizes the IP coverage using a local median window of 1000 bp and generates a normalized coverage plot.

In R:

# Load INPUT file (normalized)

input_file <- read.csv("file_INPUT_adjusted.csv") # Replace with normalized INPUT file

colnames(input_file) <- c("X", "pos", "cov") # Assign column names

# Load IP file

ip_file <- file_IP # Replace with IP file

colnames(ip_file) <- c("X", "pos", "cov") # Assign column names

# Parameters for analysis

locus_coord <- XXX # Replace with the genomic coordinate for the locus of interest

local_window <- 1000 # Define the local window size

# Normalize by a local window around the locus

local_start <- max(1, locus_coord - local_window) # Ensure bounds are valid

local_end <- min(nrow(input_file), locus_coord + local_window)

local_median <- median(input_file$cov[local_start:local_end]) # Compute local median

# Normalize IP coverage using the local median

normalized_cov <- ip_file$cov / local_median

normalized_data <- data.frame(pos = input_file$pos, cov = normalized_cov)

# Generate the normalized coverage plot

plot(

x = normalized_data$pos[local_start:local_end],

y = normalized_data$cov[local_start:local_end],

type = "l",

xlab = "Genomic Coordinates",

ylab = "Normalized Coverage (IP/INPUT)",

main = "Normalized Coverage",

col = "blue"

)
