# Supplementary material for: Dynamic transitions of initiator binding coordinate the replication of the two chromosomes in Vibrio cholerae
Source: Nat Commun. 2025 Jan 8;16:485. doi: 10.1038/s41467-024-55598-9 (PMC11711613; doi:10.1038/s41467-024-55598-9)
Supplement: Supplementary file 9 — Transparent Peer Review file [file 41467_2024_55598_MOESM9_ESM.pdf]

nature portfolio

Peer Review File

Dynamic transitions of initiator binding coordinate the replication of the two chromosomes in Vibrio cholerae

Corresponding Author: Dr Marie-Eve Val

This file contains all reviewer reports in order by version, followed by all author rebuttals in order by version.

Version 0:

Reviewer comments:

Reviewer #1

(Remarks to the Author)
Some bacteria contain multiple replicons and how these species coordinate the replication of different replicons is an outstanding question. Currently, V. cholerae is the only model bacterium in which a specific regulation system is identified: the replication of the crtS sequence on Chr1 triggers Chr2 replication initiation, and this trigger signal seems to be mediated by the initiator of Chr2 replication, RctB. Niault et al aimed to understand the molecular mechanism of this regulation. The authors used ChIP-seq to find the binding sites of RctB in different strains and in different point of replication cycle, and proposed that RctB shifts its binding from inhibitory sites to initiation sites after replication of crtS. The molecular mechanism of how duplication of crtS shifts RctB binding is not addressed.

Specific points:

1) In the model proposed by the authors, RctB bridges the 29m/39m at ori2 to form a DNA loop, and RctB bridges the interaction between crtS and ori2. TEM was used to provide evidence for the intra-molecular (Fig. 2b) and inter-molecular interactions (Fig.S9). But these TEM experiments are missing critical controls. In Fig 2b, S9a and S9b, the positions of contact points need to be analyzed to address whether these contact points correlate with the position of 29/39ms or crtS. For Fig 2b, mutant DNA (e.g. 29m* C/A) should be used in TEM to show the absence of loops. Similarly, in S9a and S9b, control experiments should be done using DNA without crtS site. Without these controls, it is premature to conclude that RctB specifically bridges 29/39m at ori2, and that the inter-molecular contacts between DNA pieces seen in TEM are related to crtS.

2) In Fig 2c, when the mutant DNA (29m* C/A) was used, RctB had higher binding to interons array and the DUE. The authors proposed that preventing RctB binding to 29m alleviates the constraint on ori2 initiation. This would predict that 29m* C/A mutant would have higher replication initiation on Chr2. However, the marker frequency analysis in Fig S12 showings that Chr2 has lower copy number in 29m* C/A than in WT. What is the explanation for this?

3) Fig 4: the data from microscopy (Fig. 4d) and MFA (Fig 4e right plot) are inconsistent. The microscopy shows that cells were born with one ori1 focus and two ori2 foci, but the MFA plot shows that ori2 copy number is lower than ori1 copy number. What is the explanation for this inconsistency?

4) Fig 3e: the bacterial-hybrid result is ambiguous. What are the blue speckles on white patches?

5) Fig 3f should be plotted on top of fig 2a for comparison.

6) Page 10, Line 10, the authors wrote “While synchronization by SHX is not as perfect in V. cholerae as it is in other bacteria, it provided a reasonable approximation to a synchronized population.” This is an overstatement. Although the replication arrest by SHX seems to be fine (Fig 5c), the replication reinitiation after SHX wash-off is rather asynchronous, i.e. in Fig 5d, Chr1 replication profile has a very shallow gradient rather than a sharp peak; in Fig 5e and f, the replication profiling of Ch1 is very similar exponentially growing, unsynchronized cells. This is not an “approximation to a synchronized population”. The authors should modify their wording according to the data and modify their interpretation of the ChIP-seq result. In Fig 5f, MFA panel, Chr2 seems to have an asymmetric replication profile, in which the right arm replicates faster than the left arm as shown by the different slopes. What is the explanation for this?

7) In Fig S4, ParB2-3FLAG ChIP-seq signal is barely above the background. Is it possible that the FLAG tag is not exposed well for successful ChIP pull down? The authors could consider a different tag or put the FLAG tag on the N-terminus of the protein. Clearer ParB2 ChIP-seq results are needed to support the authors' conclusions.

Reviewer #2

(Remarks to the Author)
The work presented in the manuscript has medical relevance because Vibrio cholerae causes disease in humans when the genome carries the TCP pathogenicity island. The study should also appeal to a broad readership because it addresses an interesting biological problem that is not understood. Vibrio cholerae has two chromosomes named Chr1 and Chr2. DNA replication of Chr2 depends on duplication of a locus, crtS encoded by Chr1. Evidence suggests that DNA replication of crtS bound by RctB leads to Chr2 DNA replication, but the molecular mechanism underlying this process is not known. The manuscript attempts to address this issue. Unfortunately, the experiments do not lead to a better understanding of the mechanism whereby duplication of the crtS locus gives rise to DNA replication of Chr2.

Major issues
Page 6, line 7-11 and Fig. 2b. The problem with the TEM data is that quantitative analysis of many complexes was not apparently performed. The authors do not state that the image analysis was unbiased; what were the criteria used to select the complexes of Fig. 2b? What was their ratio compared with other DNAs? Were negative controls analyzed by TEM such as the 29m* mutant DNA?

Page 9, line 22-28 and Supplementary Fig. 9. The problem is like that raised for Fig. 2b and the text on page 6.

Page 10, line 22-23. Figure 5c-f doesn't convincingly show that RctB leaves 39m or that it also dissociates from 29m and then goes to the iterons and the DUE. Another explanation is that RctB newly synthesized after the 45 min time point localizes to the iterons, the DUE and to 29m. This key experiment is central to the manuscript (Title, Abstract, Results, Discussion, and the models proposed).

Fig. 5c-f. Are the results reproducible?

Minor problems
Supplementary Fig. 2. For consistency with Fig. 2, please add the 39m, iteron and 29m sites to the map at the top of ori2 of Supplementary Fig. 2.

Page 5, line 27-28 and Supplementary Fig. 4. The results are _consistent_ with competitive binding of ParB2 and RctB. Please revise the text.

Page 5, line 30 and Supplementary Fig. 5b. Shouldn't RctB bind to the iterons in the dam+ strain?

Fig. 2a versus 2c. Why wasn't RctB observed to bind to the iterons and the DUE in Fig. 2a?

Page 6, line 31. Please replace "predicted" with a more appropriate word. The SEC experiment does not predict that RctBIV is a dimer.

Fig. 3d and 3c. Are the 2D class averages consistent with a RctBIV tetramer? The text on page 7 describing Fig. 3d is not clear.

Page 7, line 35. The ChIP-seq profiles are not the same but essentially the same. Please revise the text.

Fig. 5a. The growth curve prior to the addition of SHX suggests that the culture was not growing exponentially.

Reviewer #3

(Remarks to the Author)
General comments:
The manuscript by Théophile Niault and colleagues considers an important question about chromosomal DNA replication initiation in pathogen Vibrio cholerae. The authors address the question of how the replication initiation of second chromosome (Chr2), also considered as mega plasmid, is controlled by the crtS site which is present on the first chromosome (Chr1). The work uncovers the mechanism that handcuffs Chr2 with Chr1, resulting in Chr2 replication initiation arrest. After Chr1 DNA replication initiation and the progression of the replication fork through the crtS site resulting crtS duplication, the Chr2 replication initiation inhibition is released. Authors propose a synchronization model in which the RctB inhibitory complex formed on crtS site and 39/29mer inhibitory site at Chr2 origin is remodeled when crtS is duplicated. It is an interesting mechanism linking Chr1 and Chr2 replication initiation together or rather timing Chr2 replication initiation with Chr1 replication initiation. The work is very interesting and important. It contributes a general understanding of the regulatory mechanisms of DNA replication. It also has important evolutionary implications, providing a mechanism in which bacteria can control mega plasmids. The work is generally well done and conclusions are well supported by the obtained data. Some modifications should be introduced to improve both text and data presentation.

Remarks to the author:
Major comments
1. The title suggests that the work provides data on cell-cycle-dependent regulatory mechanism. It is rather the mechanism that coordinates the replication of multiple replicons in the same cell. However, the Chr1 replication initiation and its correlation with cell cycle do not depend on crtS and RctB at all. The crtS and RctB are replicon elements and are not directly linked with cell cycle. The described mechanism timely links the replication initiation of Chr2 with the replication of Chr1. The title could be corrected to more precisely describe and highlight the contents of the work.
2. The discussion is very short. The work would benefit with some general statements pointing out the importance of obtained data in understanding evolutionary aspects. How plasmids were domesticated and controlled. The work opens the door to elucidate on this.
3. The TEM experiments require clarification and better presentation. In Supplementary Figure 9 b, the specimens should be measured, identified and marked (or a graphic drawing provided showing intermolecular coupling between DNA fragments). What fragments are involved? What is the statistic? It is also unclear what is observed when RctB complex is analyzed on a 944bp DNA fragment containing a single crtS site (panel a). It looks like a loop. Please comment?
Minor comments
1. In “Abstract, "crtS replicates" . crtS site cannot replicate itself. It is not a replicon. It would be more accurate to say that it can be replicated or duplicated after the progression of the replication fork. Abstract text could be improved for more precision.
2. “Introduction” Please add information about the oligomeric conformations of RctB
3. Page 3 lines 11-12 (mechanism?)
4. Page 4 lines 29-34 Although the obtained result is clear, the description of the experiment design is very elusive. It should be described better in the figure legend maybe.
5. Figure 1. The Figure axis description should be corrected. It is not as in the Figure legend description.
6. Page 5 lines 21-23 The statement is misleading or rather over-interpreted at the moment. In the exponential phase, an unsynchronized culture was used, so replicating and nonreplicating cells were present. No conclusion should be made on the RctB interaction with 29/39. Someone can speculate that RctB interacts 29/39 when DNA replication is arrested and does not interact when it is initiated.
7. Based on the results presented in Fig. 2a, it is unclear if RctB is not interacting iterons. The normalized IP/Input is different in panels a and b. Could it be compared? Is it possible that the observed bulge/hump in iterons/DUE on the panel a is a replicating Chr2. Please comment on it.
8. Page 7 lines 4-5 and lines 8-9 The two sentences are generally repetition. No data is provided on RctB oligomeric structures on DNA. The data provided suggests that the protein can form different oligomeric assemblies. The statement lane 5 "when recruited to DNA" should be deleted. Please correct the repetition.
9. Page 7 lines 16-17 It will be accurate to say “that one RctB molecule could interact with other RctB molecule”
10. Page 7 lines 24-25 It is not clear what results and how?
11. Page 8 line 9 The sentence (title/statement) is a shortcut. Replication initiation? Involving crtS? Inhibitory complex? Please correct.
12. Page 8 lines 26-27 The sentence should be corrected please delete "direct." Please see also general comment 2 on TEM experiments.
13. Page 9 lines 9-10 The sentence requires correction.
14. Page 9 lines 21-22 The sentence requires correction. It is rather “the location (position) of the crtS on Chr1 affect on the time (timing) of replication initiation of Chr2....”
15. Figure 1 legend …attTn7 site or LacZ ? …region containing RctB binding site…?
16. Figure 3 legend (DP) DP is not used anymore. Please delete.
17. Figure 4 Representative images of cells containing the analyzed fluorescent signals could be added to better visualize and document the obtained data.
18. Figure 5 Why is OD decreased after SHX addition?
19. Figure 5 The Figure panels should be rearranged for better data presentation. Panels a and b could be moved to supplement. ChIP datais placed in one column (15, 30, 45 and 60 min) and on the side the MFA data (15, 30, 45 and 60 min).
20. Figure 5 What is the sample collection time? SHX incubation seems to be longer than 120 min. (as stated in the text in the figure legend and methods).

Version 1:

Reviewer comments:

Reviewer #1

(Remarks to the Author)
The authors have done a great job in the revision. The added experiments, analyses, and edits greatly improved the manuscript. I have no further concerns.

Reviewer #2

(Remarks to the Author)
Thank you for analyzing the protein-DNA complexes in more detail. Have you considered only analyzing complexes in which the approximate diameter of the bound protein was in the range of 10 nm? What evidence supports the idea that the

large complexes presumed to arise by aggregation are functional? Specifically, some of the panels of Figure S6 and S12 show very large protein aggregates, which seem incompatible with the model of Figure 6. Is it possible that DnaK and DnaJ remain bound to RctB to explain the observations?

Page 6, line 22. Please delete “known to . . . in vivo.”

Page 16, line 20-21. The statement that the DNA was divided into 10 nm segments is not correct.

Reviewer #3

(Remarks to the Author)
The revised version of the manuscript is improved. The title and the text were modified to better describe the experimental content. The discussion section was improved by incorporating broader evolutionary insights. The major improvement brings also the more extensive and more detailed TEM data analyses. Authors reorganized TEM data presentation. It is now presented in Figure 2, Figure S6 and Figure S12. The interaction of RctB with 39/29mer sites on Chr2 DNA fragments is now well documented. TEM experiments demonstrating nucleoprotein complex involving ctrS site, 39/29mer sites bridged by RctB is less convincing. The TEM images are essentially as in previous version. Authors point out that this complex is rarely observed. The application of crosslinking could possibly solve this problem in future investigations. This complex is an important element in the proposed model. Regardles of the RctB detection on ctrS site demonstrated in ChiPseq experiments it would be reasonably to consider transient dissociation of RctB from ctrS when replisome passes the site. All minor point were specifically addressed which improved and clarified text of the manuscript. The work is an important contribution to understanding of the regulatory mechanisms of DNA replication of Vibrio cholerae chromosomes. The proposed synchronization model assumes that the RctB inhibitory complex formed on crtS site and 39/29mer inhibitory site at Chr2 origin is remodeled when crtS is duplicated after fork progression on Chr1. The model is predominately supported by extensive ChIPseq analyses demonstrating RctB occupancy of 39/29mer sites on Chr2 during Chr1 fork progression.

Open Access This Peer Review File is licensed under a Creative Commons Attribution 4.0 International License, which permits use, sharing, adaptation, distribution and reproduction in any medium or format, as long as you give appropriate credit to the original author(s) and the source, provide a link to the Creative Commons license, and indicate if changes were made.
In cases where reviewers are anonymous, credit should be given to 'Anonymous Referee' and the source.
The images or other third party material in this Peer Review File are included in the article’s Creative Commons license, unless indicated otherwise in a credit line to the material. If material is not included in the article’s Creative Commons license and your intended use is not permitted by statutory regulation or exceeds the permitted use, you will need to obtain permission directly from the copyright holder.
To view a copy of this license, visit https://creativecommons.org/licenses/by/4.0/

We wish to sincerely thank all reviewers for their thoughtful and constructive comments.

In our study, we observed a transition in RctB binding from inhibitory to activatory sites at *ori2* following *crtS* replication. We show that *ori2* replication inhibition appears to be mediated by RctB-induced loops between inhibitory sites, which may prevent RctB from binding to activatory sites. Our structural data suggest that RctB can multimerize via its domain IV, playing a critical role in this inhibitory binding. Mutating residues involved in the dimerization interface of domain IV appears to release RctB from inhibitory sites, allowing it to bind to activatory sites. Consequently, *crtS* is no longer required for this transition and Chr2 replication initiation. Our findings also indicate that *crtS* activation upon duplication follows a one-to-one activation model at *ori2*, as demonstrated by fluorescence microscopy.

Based on all these observations, we propose a model in which *crtS* duplication destabilizes the inhibitory complex mediated by RctB domain IV, facilitating RctB binding to activatory sites at *ori2*. To our knowledge, this transition in the binding of a replication initiator from one type of site to another during the cell cycle has not been previously observed, marking a novel and significant contribution to the field. While there is still more to uncover, our findings represent an important step toward understanding how *crtS* coordinates Chr2 replication initiation and provide a strong foundation for future research.

Reviewer #1 (Remarks to the Author):

Some bacteria contain multiple replicons and how these species coordinate the replication of different replicons is an outstanding question. Currently, V. cholerae is the only model bacterium in which a specific regulation system is identified: the replication of the crtS sequence on Chr1 triggers Chr2 replication initiation, and this trigger signal seems to be mediated by the initiator of Chr2 replication, RctB. Niault et al aimed to understand the molecular mechanism of this regulation. The authors used ChIP-seq to find the binding sites of RctB in different strains and in different point of replication cycle and proposed that RctB shifts its binding from inhibitory sites to initiation sites after replication of crtS. The molecular mechanism of how duplication of crtS shifts RctB binding is not addressed.

We thank the reviewer for their valuable feedback. Please find below a point-by-point response to the reviewer’s specific comments.

Specific points:

1) In the model proposed by the authors, RctB bridges the 29m/39m at ori2 to form a DNA loop, and RctB bridges the interaction between crtS and ori2. TEM was used to provide evidence for the intra-molecular (Fig. 2b) and inter-molecular interactions (Fig.S9). But these TEM experiments are missing critical controls. In Fig 2b, S9a and S9b, the positions of contact points need to be analyzed to address whether these contact points correlate with the position of 29/39ms or crtS. For Fig 2b, mutant DNA (e.g. 29m* C/A) should be used in TEM to show the absence of loops. Similarly, in S9a and S9b, control experiments should be done using DNA without crtS site. Without these controls, it is premature to conclude that RctB specifically bridges 29/39m at ori2, and that the inter-molecular contacts between DNA pieces seen in TEM are related to crtS.

We thank reviewer #1 for providing insightful comments and the opportunity to clarify and reinforce our TEM experiments.

In response to the concern regarding the absence of critical controls in our TEM experiments, we have now conducted additional TEM experiments incorporating more rigorous controls. These experiments included five different DNA fragments:

-F1 (Full-length ori2): Contains all three inhibitory sites (39mL, 39mR, 29m) – 1793 bp.
-F2 (Two sites, 39mL-39mR): Lacking the 29m site – 1555 bp.
-F3 (Single site, 29m): Contains only the 29m site – 1617 bp.
-F4 (crtS site): Contains only the crtS site – 944 bp.
-F5 (Control, no sites): No inhibitory sites present – 1984 bp.

For each DNA fragment, we characterized at least 100 molecules, capturing 100 images at a consistent magnification (X28980), with each image containing at least one DNA molecule. Asymmetrical site distribution allowed us to orient the fragments and measure the occupancy of RctB along the DNA molecules. Each DNA molecule was divided into 10 nm segments, and the presence or absence of RctB binding (density) was quantified. The results are shown in Supplementary Figures 6 and 12. Quantification confirmed that the RctB binding matches the theoretical location of its specific sites, with occasional binding observed at other locations, which we attribute to non-specific DNA scanning. This indicates that RctB predominantly binds to the expected sites with high specificity.

We also analyzed the proportion of loop-containing DNA fragments. A protein-mediated DNA loop was defined as a loop with increased density at the base, indicating the presence of RctB. The following results were observed:

-F1 (three sites, 39mL-39mR-29m): 53% of observed fragments contained loops.
-F2 (two sites, 39mL-39mR): 37% of observed fragments contained loops.
-F3 (one site, 29m): Only 12% of observed fragments contained loops.
-F4 and F5: No loops were observed.

These data, presented in Supplementary Figure 7, demonstrate that loop formation is significantly enhanced with multiple 39m sites, confirming that RctB specifically induces loop formation between these sites. We hypothesize that the loops observed in fragment F3 arise from interactions between RctB bound specifically to 29m and RctB bound nonspecifically to other DNA regions.

Regarding the suggestion to use the 29mC/A* mutant : We chose not to use the 29mC/A* mutant in these experiments, as our previous study (Fournes, NAR, 2021 PMC8565311) showed that this mutation has minimal to no impact on RctB binding *in vitro* (though it completely abolishes binding *in vivo*). Due to this discrepancy, we choose to use fragments with full deletion of 39m sites rather than partial mutations, providing clearer insights into the role of these sites in loop formation.
We added this fact to the text : "To test this hypothesis, we performed a ChIP-seq of RctB in a mutant with the C21 > A mutation at the 29m site (29m*(C/A)) known to disrupt RctB binding ***in vivo***"

About the intermolecular contacts involving *crtS* : As previously reported, contacts between crtS and 39m sites were observed in fewer than 1% of molecules. While we acknowledge the importance of these interactions, their infrequency impeded quantification. However, we believe it is still valuable to report these findings in supplementary figure, even though they fall outside the primary scope of this study. Future work using other approaches, such as Cryo-EM, may further clarify these interactions.

These additional controls and analyses provide stronger evidence that RctB specifically bridges the 39m sites at *ori2* to form DNA loops. We hope these new experiments and clarifications address the reviewer's concerns and further solidify the conclusions of our study.

2) In Fig 2c, when the mutant DNA (29m* C/A) was used, RctB had higher binding to iterons array and the DUE. The authors proposed that preventing RctB binding to 29m alleviates the constraint on ori2 initiation. This would predict that 29m* C/A mutant would have higher replication initiation on Chr2. However, the marker frequency analysis in Fig S12 showings that Chr2 has lower copy number in 29m* C/A than in WT. What is the explanation for this?

The experiments now presented in Figure S17 were conducted using a strain with both the 29m* C/A mutation and a deletion of the crtS site (ΔcrtS). This combined genotype is essential for understanding the context of the observed results. Comparing the marker frequency analysis (MFA) profiles between the ΔcrtS strain and the double mutant strain (29m* C/A, ΔcrtS), we found that the copy number of Chr2 is indeed higher in the 29m* C/A ΔcrtS strain than in the ΔcrtS strain alone. This observation is consistent with our previous findings (Val, Science Advances, 2016, PMID: 27152358, Fig. S14 strain D247), where the 29m* mutation led to increased copy numbers in the absence of crtS.

The observed MFA profiles support our model. In the double mutant strain (29m* C/A ΔcrtS), Chr2 replication initiation no longer depends on crtS, as reflected by the elevated Chr2 levels, nearly matching those of the wt strain. Without crtS to synchronize replication, we hypothesize that Chr2 initiations occur randomly, with some starting at the beginning of the cell cycle and others initiating later.

For added clarity, we specified in the figure that the strain is a double mutant (29m* C/A and ΔcrtS) and updated the legend accordingly.

3) Fig 4: the data from microscopy (Fig. 4d) and MFA (Fig 4e right plot) are inconsistent. The microscopy shows that cells were born with one ori1 focus and two ori2 foci, but the MFA plot shows that ori2 copy number is lower than ori1 copy number. What is the explanation for this inconsistency?

We thank reviewer #1 for his comment.
We believe that the apparent discrepancy lies in the different growth conditions. In microscopy, we use minimal medium (M9 + fructose), whereas in MFA, cells are grown to steady state in LB medium. Previously, we reported that in the strain with two crtS sites in stationary phase, the Chr2 copy number in LB is slightly below 2 (Fig. 1E, de Lemos Martins, 2018, doi: 10.1093/nar/gky790). We think this could be due to asymmetric division, which sometimes leads to an uneven distribution of Chr2 (where, after division, a cell ends up with 1 Chr2). This problem could occur more frequently under fast growth conditions. In the M9 medium used for microscopy, we observed a few cells with only 1 Chr2 (Fig. 4b), probably resulting from such asymmetric division, which would occur less frequently under slower

growth conditions. Microscopy growth conditions cannot fully reproduce the replication dynamics observed in the steady-state culture used for MFA, on which we rely to simulate our replication model. We would like to refer the reviewer to Supplementary movie 3, which visually illustrates these replication dynamics and the timing differences between Chr1 and Chr2 replication initiation.

4) Fig 3e: the bacterial-hybrid result is ambiguous. What are the blue speckles on white patches?

The blue speckles observed on the white patches in Fig. 3e are likely lac+ revertants, which are a common occurrence in bacterial adenylate cyclase two-hybrid (BACTH) experiments (as noted in Olson, Methods in Molecular Biology, 2018, PMID: 29855952). These revertants can cause isolated blue dots within otherwise white colonies. However, these blue speckles do not reflect the overall interaction strength or the primary outcome of the experiment.
To accurately assess the interactions, we focus on the global color of the colony rather than the isolated blue speckles. When comparing our mutants to the positive controls (Zip-Zip and D314P-D314P), the mutants exhibit a clearly less blue phenotype overall. This indicates weaker interaction and supports our conclusions about the nature of these mutations.

Text change: added to Figure 3 legend "Blue colonies indicate a positive protein-protein interaction, while white colonies indicate no interaction. Any blue dots within white colonies are due to *lacZ+* revertant and should not be considered as evidence of interaction."

5) Fig 3f should be plotted on top of fig 2a for comparison.

We understand the reviewer's suggestion to plot Fig. 3f on top of Fig. 2a for direct comparison. However, these figures represent ChIP-seq results for two different proteins (RctB-wt in Fig. 2a and RctB-L651P in Fig. 3f), each with different scales and signal intensities. Due to these differences, direct comparison on the same plot would not be meaningful. The reproducibility of the ChIP-seq results is strong when comparing replicates of the same protein, but not necessarily across different proteins with distinct binding properties. Therefore, combining these plots could lead to misinterpretation of the data.
We believe that keeping the plots separate provides a clearer and more accurate representation of the individual binding patterns and differences between the wild-type and mutant protein.

6) Page 10, Line 10, the authors wrote "While synchronization by SHX is not as perfect in V. cholerae as it is in other bacteria, it provided a reasonable approximation to a synchronized population." This is an overstatement. Although the replication arrest by SHX seems to be fine (Fig 5c), the replication re-initiation after SHX wash-off is rather asynchronous, i.e. in Fig 5d, Chr1 replication profile has a very shallow gradient rather than a sharp peak; in Fig 5e and f, the replication profiling of Ch1 is very similar exponentially growing, unsynchronized cells. This is not an "approximation to a synchronized population". The authors should modify their wording according to the data and modify their interpretation of the ChIP-seq result. In Fig 5f, MFA panel, Chr2 seems to have an asymmetric replication profile, in which the right arm replicates faster than the left arm as shown by the different slopes. What is the explanation for this?

We appreciate Reviewer#1 detailed observations and agree that clarification is necessary. We agree that the original statement regarding SHX synchronization in *Vibrio cholerae* may have been overstated.

While the SHX method for synchronization is not perfect, it has been previously published and utilized in *Vibrio cholerae* studies (Kemter, PLoS Genet, 2018, PMID: 29505558). Despite its limitations, SHX remains the only known method to achieve any level of synchronization in *Vibrio cholerae* cells. To address his concern, we will revise the text to more accurately describe the process as replication initiation arrest, completion of the replication cycle, and re-initiation of replication. This revised wording better reflects the observed replication dynamics and avoids suggesting that full synchronization is achieved.

Text change: from "While synchronization by SHX is not as perfect in *V. cholerae* as it is in other bacteria, it provided a reasonable approximation to a synchronized population. Thus, we decided to use it to perform ChIP-seq of RctB at different time points after removal of SHX (15, 30, 45, 60 minutes) to follow the binding cycle of RctB." **to** "While SHX synchronization in V.cholerae is not perfect and does not achieve full synchronization, it is a useful method for studying replication dynamics in this organism. We used this method to arrest replication initiation, allow completion of the replication cycle, and then re-initiate replication. This approach enabled us to perform ChIP-seq of RctB at different time points after SHX removal (15, 30, 45, 60 minutes) to track the binding cycle of RctB."

Regarding the asymmetric replication profile observed for Chr2, particularly the difference in slopes between the right and left arms. The left arm contains the superintegron region (approximately 130 kb). This region is known to exhibit higher coverage in MFA than the rest of the genome, as previously reported (Richard, Science Advances, 2024, PMID: 38215203, Fig. S8). This increased coverage likely accounts for the observed asymmetric replication profile.

7) In Fig S4, ParB2-3FLAG ChIP-seq signal is barely above the background. Is it possible that the FLAG tag is not exposed well for successful ChIP pull down? The authors could consider a different tag or put the FLAG tag on the N-terminus of the protein. Clearer ParB2 ChIP-seq results are needed to support the authors' conclusions.

While it is true that the ParB signal at *ori2* is not particularly high, we observed clear enrichment for all the other *parS2* sites on Chr2, as shown in Supp Fig. 4a. The reduced presence of ParB at the *parS* site on *ori2* may have biological significance, potentially indicating interference or competition with RctB binding to 39mL. Importantly, this pattern was consistently observed in two independent experiments, suggesting that it is a reproducible and meaningful result. Additionally, a ParB2 ChIP-on-chip experiment has previously been conducted, and although this technique provides less resolution, it revealed a similar pattern for ParB2 binding (Venkova-Canova, PLoS Genet, 2013, PMCID: PMC3688505). This consistency across different experimental approaches reinforces our findings.

Reviewer #2 (Remarks to the Author):

The work presented in the manuscript has medical relevance because Vibrio cholerae causes disease in humans when the genome carries the TCP pathogenicity island. The study should also appeal to a broad readership because it addresses an interesting biological problem that is not understood. Vibrio cholerae has two chromosomes named Chr1 and Chr2. DNA replication of Chr2depends on duplication of a locus, crtS encoded by Chr1. Evidence suggests that DNA replication of crtS bound by RctB leads to Chr2 DNA replication, but the molecular mechanism underlying this process is not known. The manuscript attempts to address this issue. Unfortunately, the experiments do not lead to a better understanding of the mechanism whereby duplication of the crtS locus gives rise to DNA replication of Chr2.

We appreciate the reviewer's positive and thoughtful feedback. Below is our point-by-point response.

Major issues:

Page 6, line 7-11 and Fig. 2b. The problem with the TEM data is that quantitative analysis of many complexes was not apparently performed. The authors do not state that the image analysis was unbiased; what were the criteria used to select the complexes of Fig. 2b? What was their ratio compared with other DNAs? Were negative controls analyzed by TEM such as the 29m* mutant DNA?

Page 9, line 22-28 and Supplementary Fig. 9. The problem is like that raised for Fig. 2b and the text on page 6.

We thank reviewer #2 for providing insightful comments and the opportunity to clarify and reinforce our TEM experiments.

In response to the concern regarding the absence of critical controls in our TEM experiments, we have now conducted additional TEM experiments incorporating more rigorous controls. These experiments included five different DNA fragments:

-F1 (Full-length ori2): Contains all three inhibitory sites (39mL, 39mR, 29m) – 1793 bp.
-F2 (Two sites, 39mL-39mR): Lacking the 29m site – 1555 bp.
-F3 (Single site, 29m): Contains only the 29m site – 1617 bp.
-F4 (crtS site): Contains only the crtS site – 944 bp.
-F5 (Control, no sites): No inhibitory sites present – 1984 bp.

For each DNA fragment, we characterized at least 100 molecules, capturing 100 images at a consistent magnification (X28980), with each image containing at least one DNA molecule. Asymmetrical site distribution allowed us to orient the fragments and measure the occupancy of RctB along the DNA molecules. Each DNA molecule was divided into 10 nm segments, and the presence or absence of RctB binding (density) was quantified. The results are shown in Supplementary Figures 6 and 12. Quantification confirmed that the RctB binding matches the theoretical location of its specific sites, with occasional binding observed at other locations, which we attribute to non-specific DNA scanning. This indicates that RctB predominantly binds to the expected sites with high specificity.

We also analyzed the proportion of loop-containing DNA fragments. A protein-mediated DNA loop was defined as a loop with increased density at the base, indicating the presence of RctB. The following results were observed:

-F1 (three sites, 39mL-39mR-29m): 53% of observed fragments contained loops.
-F2 (two sites, 39mL-39mR): 37% of observed fragments contained loops.
-F3 (one site, 29m): Only 12% of observed fragments contained loops.
-F4 and F5: No loops were observed.

These data, presented in Supplementary Figure 7, demonstrate that loop formation is significantly enhanced with multiple 39m sites, confirming that RctB specifically induces loop formation between these sites. We hypothesize that the loops observed in fragment F3 arise from interactions between RctB bound specifically to 29m and RctB bound nonspecifically to other DNA regions.

Regarding the suggestion to use the 29mC/A* mutant : We chose not to use the 29mC/A* mutant in these experiments, as our previous study (Fournes, NAR, 2021 PMC8565311) showed that this mutation has minimal to no impact on RctB binding in vitro (though it completely abolishes binding in vivo). Due to this discrepancy, we choose to use fragments with full deletion of 39m sites rather than partial mutations, providing clearer insights into the role of these sites in loop formation.
We added this fact to the text : "To test this hypothesis, we performed a ChIP-seq of RctB in a mutant with the C21 > A mutation at the 29m site (29m*(C/A)) known to disrupt RctB binding in vivo"

About the intermolecular contacts involving crtS : As previously reported, contacts between crtS and 39m sites were observed in fewer than 1% of molecules. While we acknowledge the importance of these interactions, their infrequency impeded quantification. However, we believe it is still valuable to report these findings in supplementary figure, even though they fall outside the primary scope of this study. Future work using other approaches, such as Cryo-EM, may further clarify these interactions.

These additional controls and analyses provide stronger evidence that RctB specifically bridges the 39m sites at ori2 to form DNA loops. We hope these new experiments and clarifications address the reviewer's concerns and further solidify the conclusions of our study.

Page 10, line 22-23. Figure 5c-f doesn't convincingly show that RctB leaves 39m or that it also dissociates from 29m and then goes to the iterons and the DUE. Another explanation is that RctB newly synthesized after the 45 min time point localizes to the iterons, the DUE and to 29m. This key experiment is central to the manuscript (Title, Abstract, Results, Discussion, and the models proposed).

We thank the reviewer for his comment.
Clarification on RctB Binding Dynamics: Our model does not suggest that RctB dissociates from the 29m or 39m sites after *crtS* duplication. Instead, we propose that the interaction between RctB molecules on 39m and 29m forms a loop structure, which maintains the inhibition of replication initiation at *ori2*. When crtS is replicated, we hypothesize that the RctB bound to *crtS* interacts with RctB either at the 29m or 39m sites, which disrupts the loop structure. This disruption reduces the steric hindrance, allowing access to the iterons and the DUE for the initiation of replication. Thus, our model does not

imply that RctB leaves the 29m or 39m sites; rather, it undergoes a structural reorganization that facilitates replication initiation.

Role of RctB Binding to Iterons and DUE: The temporal shift in ChIP-seq signals observed in now Figure 5d suggests an increase in RctB association with the iterons and DUE. We interpret this as indicative of a reorganization rather than complete dissociation from inhibitory sites. Newly synthesized RctB could contribute to this observed pattern; however, the timing of *crtS* replication and subsequent changes in RctB binding at *ori2* are consistent with our model that *crtS* replication triggers the disruption of the inhibition complex, allowing RctB to access the iterons and DUE more effectively.

<u>We have corrected the text as follows:</u>

-Original “Here, we were able to capture a release of RctB from the inhibitory sites (29/39m) towards the activating sites (iteron) and towards the DUE. This temporal shift in the binding pattern of RctB from inhibitory to activating sites on ori2 underscored the opening of ori2 that resulted in the initiation of Chr2 replication. As expected, ori2 activation follows crtS replication on Chr1.”

-Revised: “Here, we detected a change in RctB binding dynamics at *ori2* over time, where RctB molecules initially primarily interact with inhibitory sites (29/39m). Subsequently, there is an increased engagement with activating sites (iteron) and the DUE. This shift suggests that rather than completely dissociating from the inhibitory sites, RctB molecules undergo a structural rearrangement that alleviates the inhibition, making the iterons and DUE accessible. This rearrangement likely disrupts the inhibitory loop, facilitating the opening of ori2 and initiating Chr2 replication. As expected, this process is triggered after *crtS* replication on Chr1.”

Fig. 5c-f. Are the results reproducible?

All ChIP-seq experiments were performed in duplicates. Only one of the duplicates for each time point is shown in the figure. Similar patterns were observed in both duplicates, confirming the reproducibility of the results. The data are publicly available on the ENA website. See “Data Materials Availability” for accession numbers, where duplicates accession numbers are now also provided.

Minor problems:

Supplementary Fig. 2. For consistency with Fig. 2, please add the 39m, iteron and 29m sites to the map at the top of ori2 of Supplementary Fig. 2.

Supplementary Fig. 2 has been modified accordingly.

Page 5, line 27-28 and Supplementary Fig. 4. The results are consistent with competitive binding of ParB2 and RctB. Please revise the text.

We have corrected the text as follows:

-Original: “Confirming our suspicion, our ChIP-seq of ParB2 on ori2 (Supplementary Fig. 4) revealed that ParB2 does indeed compete with RctB at both 39m sites and not at the 29m site.”

-Revised: “The ChIP-seq results of ParB2 on ori2 (Supplementary Fig. 4) are consistent with competitive binding of ParB2 and RctB at both 39m sites and not at the 29m site.”

-Page 5, line 30 and Supplementary Fig. 5b. Shouldn’t RctB bind to the iterons in the dam+ strain? AND Fig. 2a versus 2c. Why wasn’t RctB observed to bind to the iterons and the DUE in Fig. 2a?

We understand from Reviewer #2’s comment that further clarification is needed. The absence of RctB binding to the iterons and the DUE in Fig. 2a is our initial observation and the question we have been trying to understand. We have revised the text to better explain this initial observation and our hypothesis.

Text revised :
“We observed that the RctB ChIP signal was about 10-fold higher at the *ori2* region than at other chromosomal loci (Supplementary Fig. 2). Notably, RctB showed a strong preference for binding to the 29/39m inhibitory sites (Fig. 2a). To further explore this binding pattern, we performed ChIP-seq on RctB during the stationary phase, when cells are not replicating (Supplementary Fig. 3). Interestingly, the RctB binding pattern at *ori2* in non-replicating cells closely mirrored what we observed during the replicating state of exponential growth. This predominant binding at the inhibitory sites suggests that Chr2 replication initiation is largely impeded throughout most of the cell cycle, reflecting similar behavior in both replicating and non-replicating cultures. Given that exponential-phase cultures consist of a mixture of cells at different stages of the replication cycle (asynchronous culture), we hypothesized that RctB interactions with the iterons and DUE are likely very transient, occurring only during short intervals of the replication cycle. This transient nature may explain why these interactions are not readily detected in a mixed population of replicating cells.”

Our model indeed supports the idea that RctB can bind to the iterons in the *dam+* strain. However, ChIP-seq data show that RctB predominantly binds to the inhibitory sites (29m and 39m) at *ori2* rather than to the iterons.

This predominant binding at the inhibitory sites represents the most frequent state of RctB interaction with *ori2* during the cell cycle, reflecting a biologically significant regulatory mechanism.

According to our model, ori2 is locked in an inhibited state for most of the cell cycle because RctB is bound to the 29m and 39m sites, maintaining a repressive loop structure.

We propose that RctB's binding to the iterons and DUE occurs, but these interactions are likely transient.

The high frequency of RctB at the inhibitory sites in our ChIP-seq data, also in a dam+ strain, aligns with this model, suggesting that the brief binding to the iterons and DUE is hard to capture in non-synchronized cell population.

Page 6, line 31. Please replace “predicted” with a more appropriate word. The SEC experiment does not predict that RctBIV is a dimer.

We have corrected the sentence as follows:

-Original: “In the crystal, RctB-IV retains a dimeric association as predicted by Size Exclusion Chromatography (SEC).”

-Revised: “In the crystal, RctB-IV retains a dimeric association, confirmed by Size Exclusion Chromatography (SEC).”

Fig. 3d and 3c. Are the 2D class averages consistent with a RctBIV tetramer? The text on page 7describing Fig. 3d is not clear.

Thanks to the reviewer for pointing this out. The 2D classes obtained from Cryo EM are for the full-length RctB. We have clarified this point and added new data in supplementary Fig. 9.

To make this more clear now this section reads like:

“Cryo electron microscopy on full length RctB shows two main class averages which, given their dimensions, are consistent with the dimeric and tetrameric forms of RctB, respectively (Fig. 3d). These findings, together with the presence of multiple intermolecular interfaces in $RctB^{IV}$, inferred from the X-ray structure, suggest that $RctB^{IV}$ may associate in multiple ways and potentially allows RctB to form higher-order oligomeric structures.
To further explore the quaternary structural arrangement of the full-length RctB in the context of our structure of domain IV, we used AlphaFold 3 to calculate the structure of the full length RctB dimer. As shown in **Supplementary Fig. 9a-c** the predicted dimeric structure recapitulates both the previous dimerization interface via domains II [14] and the dimerization via domain IV (this work). The formation of this oligomeric structure involves a total interface of 4327 Å. This dimeric arrangement suggests the homodimer is likely a very stable state. The two independent dimerization cores perfectly superpose with the X-ray structures of the truncated domains, which are connected by highly flexible interdomain linking regions. Indeed, SEC of pure full length RctB, shows a complex pattern of two peaks consistent with the dimeric and tetrameric forms of RctB (**Supplementary Fig. 9d-e**).“

Page 7, line 35. The ChIP-seq profiles are not the same but essentially the same. Please revise the text.

We have corrected the sentence as follows:

-Original: “However, we observed the same binding profile at crtS between RctB-L651P and RctB wt”
-Revised: “However, we observed similar binding profile at crtS between RctB-L651P and RctB wt”

Fig. 5a. The growth curve prior to the addition of SHX suggests that the culture was not growing exponentially.

We agree with Reviewer #2 that the culture was not in the fastest phase of exponential growth. To accurately reflect the growth phase, we have revised the text to specify that the culture was in mid-log phase at the time of SHX addition.

-Original: “In our hands, the addition of SHX to 1.5mg/mL final concentration to an exponentially growing culture of V. cholerae caused an instantaneous growth arrest (Fig. 5a).”

-Revised: “In our hands, the addition of SHX to 1.5mg/mL final concentration to a mid-log growing culture of V. cholerae caused an instantaneous growth arrest (Supplementary Fig. 15a).”

Reviewer #3 (Remarks to the Author):

General comments:

The manuscript by Théophile Niault and colleagues considers an important question about chromosomal DNA replication initiation in pathogen Vibrio cholerae. The authors address the question of how the replication initiation of second chromosome (Chr2), also considered as megaplasmid, is controlled by the crtS site which is present on the first chromosome (Chr1). The work uncovers the mechanism that handcuffs Chr2 with Chr1, resulting in Chr2 replication initiation arrest. After Chr1 DNA replication initiation and the progression of the replication fork through the crtS site resulting crtS duplication, the Chr2 replication initiation inhibition is released. Authors propose a synchronization model in which the RctB inhibitory complex formed on crtS site and 39/29merinhibitory site at Chr2 origin is remodeled when crtS is duplicated. It is an interesting mechanism linking Chr1 and Chr2 replication initiation together or rather timing Chr2 replication initiation withChr1 replication initiation. The work is very interesting and important. It contributes a general understanding of the regulatory mechanisms of DNA replication. It also has important evolutionary implications, providing a mechanism in which bacteria can control mega plasmids. The work is generally well done and conclusions are well supported by the obtained data. Some modifications should be introduced to improve both text and data presentation. Remarks to the author:

We acknowledge Reviewer #3 for the positive feedback. Below is a point-by-point response to the comments.

Major comments

1. The title suggests that the work provides data on cell-cycle-dependent regulatory mechanism. It is rather the mechanism that coordinates the replication of multiple replicons in the same cell. However, the Chr1 replication initiation and its correlation with cell cycle do not depend on crtS and RctB at all. The crtS and RctB are replicon elements and are not directly linked with cell cycle. The described mechanism timely links the replication initiation of Chr2 with the replication of Chr1. The title could be corrected to more precisely describe and highlight the contents of the work.

We agree with the reviewer that the title should be more descriptive of the work. We have changed the title to :

Dynamic transition of initiator binding coordinates replication of Vibrio’s two chromosomes

2. The discussion is very short. The work would benefit with some general statements pointing out the importance of obtained data in understanding evolutionary aspects. How plasmids were domesticated and controlled. The work opens the door to elucidate on this.

We appreciate the reviewer’s suggestion to expand the discussion by incorporating broader evolutionary insights. Indeed, the results of our study offer valuable implications for understanding the evolution and control of multipartite genomes.

We added this paragraph to the discussion: "Other Bacterial species with multipartite genome, such as *Agrobacterium tumefaciens* (Ren, PNAS, 2021 - Martin, NAR, 2024) or Pseudoalteromonas (Xie, mBio, 2021), exhibit synchronous termination of secondary chromosome replication with that of the primary chromosome, as demonstrated by MFA during exponential growth. This suggests that coordinated replication timing between chromosomes is a conserved evolutionary feature in species with multiple chromosomes. The mechanisms we have uncovered for the regulation of the secondary chromosome in *V. cholerae* raise interesting questions about how plasmids were domesticated and controlled as they evolved into stable secondary chromosomes. Future studies could further explore these evolutionary parallels by conducting ChIP-seq experiments on the initiators of secondary chromosomes in other bacteria. Investigating whether these initiators bind to checkpoints on the primary chromosome and whether their binding dynamics vary throughout the cell cycle could shed light on the evolutionary transition from plasmid to chromosome. Such research would expand our understanding of how bacteria have evolved to stably maintain and regulate multipartite genomes, offering insights into the broader principles of genome organization and evolution."

3. The TEM experiments require clarification and better presentation. In Supplementary Figure 9 b, the specimens should be measured, identified and marked (or a graphic drawing provided showing intermolecular coupling between DNA fragments). What fragments are involved? What is the statistic? It is also unclear what is observed when RctB complex is analyzed on a 944bp DNA fragment containing a single crtS site (panel a). It looks like a loop. Please comment?

Thank you for your valuable comments. In response to the concerns raised:

When we mixed DNA substrates containing unmethylated *ori2* and *crtS* with RctB, we observed rare RctB-mediated contacts between *ori2* and *crtS* in less than 1% of the complexes (Supplementary Fig. 9b). This suggests the possibility of interactions between RctB molecules bound to crtS and those bound to the 29/39m inhibitory sites at ori2.

Due to the rarity of these interactions, they are extremely challenging to quantify accurately but we believe they provide valuable insights into potential intermolecular interactions.

As for Supplementary Fig. 9a, the structure observed on the 944bp DNA fragment containing the crtS site does indeed resemble a loop. This could reflect a structural conformation induced by RctB when binding to crtS, although the exact mechanism remains unclear. We have now replaced this figure by a larger analysis (Supp. Fig. 12a)

The binding of RctB independently to ori2 and crtS are now shown in Supp Figure 6 & 12. The rare event of RctB-mediated contacts between ori2 and crtS are shown in Supp Figure 12b.

Minor comments

1. In "Abstract, "crtS replicates". crtS site cannot replicate itself. It is not a replicon. It would be more accurate to say that it can be replicated or duplicated after the progression of the replication fork. Abstract text could be improved for more precision.

We have corrected the sentence as follows:

-Original: “This inhibitory effect is counteracted when crtS replicates on Chr1”

-Revised: “This inhibitory effect is counteracted when crtS is replicated on Chr1”

2. “Introduction” Please add information about the oligomeric conformations of RctB

We thank the reviewer for the suggestion. We added this sentence to the introduction: “The C-terminal domain (IV) is essential for down-regulating Chr2 initiation and has been shown in vitro to play a crucial role in self-interaction, promoting cooperative DNA binding and oligomerization”

3. Page 3 lines 11-12 (mechanism?)

We have corrected the sentence as follows:

-From: “The objective of the present study is to elucidate the molecular mechanisms by which crtS orchestrates this synchronization process.”

-To: “The objective of the present study is to elucidate the molecular mechanism by which crtS orchestrates this synchronization process.”

4. Page 4 lines 29-34 Although the obtained result is clear, the description of the experiment design is very elusive. It should be described better in the figure legend maybe.

We have corrected the legend as follows:

-in fig1, legend c, we added the sentence: “pORI2 copy number (pORI2/oriC) in E. coli strains containing different RctB binding regions inserted into the lacZ locus. Each E. coli strain carries a plasmid (pORI2) that replicates using the Vibrio cholerae Chr2 origin (ori2). The ratio of pORI2 to the chromosomal origin (oriC) serves as a measure of ori2 replication activity. By inserting specific RctB binding regions into the genome, we can assess how these regions influence the replication of pORI2”

5. Figure 1. The Figure axis description should be corrected. It is not as in the Figure legend description.

We'd like more clarification on the issues concerning the axis description in Figure 1, as it seems consistent with the figure legend. If there is any particular inconsistency or point of confusion, we'd be happy to modify it.

6. Page 5 lines 21-23 The statement is misleading or rather over-interpreted at the moment. In the exponential phase, an unsynchronized culture was used, so replicating and nonreplicating cells we represent. No conclusion should be made on the RctB interaction with 29/39. Someone

can speculate that RctB interacts 29/39 when DNA replication is arrested and does not interact when it is initiated.

We would like to clarify that, although the culture was unsynchronized during the exponential phase, all cells are actively replicating, just not at the same stage of the cell cycle. We thank Reviewer #3 for highlighting this point of confusion. We have modified the text to clarify this potentially misleading passage as follows :

"Interestingly, the RctB binding pattern at *ori2* in non-replicating cells closely mirrored what we observed during the replicating state of exponential growth. This predominant binding at the inhibitory sites suggests that Chr2 replication initiation is largely impeded throughout most of the cell cycle, reflecting similar behavior in both replicating and non-replicating cultures. Given that exponential-phase cultures consist of a mixture of cells at different stages of the replication cycle (asynchronous culture), we hypothesized that RctB interactions with the iterons and DUE are likely very transient, occurring only during short intervals of the replication cycle. This transient nature may explain why these interactions are not readily detected in a mixed population of replicating cells."

7. Based on the results presented in Fig. 2a, it is unclear if RctB is not interacting iterons. The normalized IP/Input is different in panels a and b. Could it be compared? Is it possible that the observed bulge/hump in iterons/DUE on the panel a is a replicating Chr2. Please comment on it.

The bulge observed in the ChIP-seq signal at the iterons/DUE region in Figure 2a is unlikely to represent RctB binding to iterons during active replication. This interpretation is supported by the ChIP-seq data obtained from the Δdam strain (Figure S5). In the Δdam strain, where RctB cannot bind iterons due to the absence of Dam methylation (as demonstrated in Figure S8b), a similar bulge is still observed. This suggests that the signal in question does not result from RctB directly binding to the iterons.

Additionally, the SHX experiment presented in Figure 5f shows that, when cells enter a replication-permissive window, RctB binding can indeed be detected at both the 39m sites and the iterons/DUE. This further supports the idea that binding to iterons and the DUE is a transient event that occurs only during active replication initiation, and is not observable in unsynchronized growing cultures.

We believe these points clarify the nature of the ChIP-seq signals observed in Figure 2a and support the conclusion that the bulge is not indicative of stable RctB binding to iterons.

We mentioned in the manuscript :
"In the Δdam#4 mutant, the RctB binding pattern at ori2 was identical to that of the wild-type strain (Supplementary Fig. 5b). This result demonstrates that RctB molecules can form a complex at ori2 strictly through their interaction with 29/39m sites, without any engagement with iterons."

8. Page 7 lines 4-5 and lines 8-9 The two sentences are generally repetition. No data is provided on RctB oligomeric structures on DNA. The data provided suggests that the protein can form different oligomeric assemblies. The statement lane 5 "when recruited to DNA" should be deleted. Please correct the repetition.

We have corrected the sentence as follows:

-Original : "These findings suggest that RctBIV may associate in multiple ways and potentially form higher-order oligomeric structures when recruited to DNA. The presence of multiple intermolecular interfaces in RctBIV inferred from the X-ray structure, together with class averages of RctB obtained by cryo electron microscopy (Fig. 3d) are consistent with the presence of dimeric and tetrameric forms RctB. These findings suggest that RctBIV may associate in multiple ways and potentially form higher-order oligomeric structures when recruited to DNA."

-Revised: "The presence of multiple intermolecular interfaces in RctBIV inferred from the X-ray structure, together with class averages of RctB obtained by cryo electron microscopy (Fig. 3d) are consistent with the presence of dimeric and tetrameric forms of RctB. These findings suggest that RctBIV may associate in multiple ways and potentially form higher-order oligomeric structures."

9. Page 7 lines 16-17 It will be accurate to say "that one RctB molecule could interact with other RctB molecule"

We have corrected the sentence as follows:

-Original: "In Fig. 3e, blue colonies (D314P/D314P) indicated that RctB could interact with other RctB through another domain, as already reported to be via domain IV"

-Revised: "In Fig. 3e, blue colonies (D314P/D314P) indicated that one RctB molecule could interact with other RctB molecule through another domain, as already reported to be via domain IV"

10. Page 7 lines 24-25 It is not clear what results and how?

We have corrected the sentence as follows:

-Original: "The I625P and L651P substitutions in two different α-helices (α3 and α6) of the C-terminal subdomain also abrogate RctB interactions confirming our previous results"

-Revised: "The I625P and L651P substitutions in two different α-helices (α3 and α6) of the C-terminal subdomain also abrogate $RctB^{IV}$-$RctB^{IV}$ interaction"

11. Page 8 line 9 The sentence (title/statement) is a shortcut. Replication initiation? Involving crtS? Inhibitory complex? Please correct.

We have revised the sentence for clarity:

-Original: "crtS exclusively affects the RctB inhibition complex at ori2"

-Revised: "crtS exclusively affects the RctB inhibition complex organization at ori2 to promote initiation"

12. Page 8 lines 26-27 The sentence should be corrected please delete "direct." Please see also general comment 2 on TEM experiments.

We have corrected the sentence as follows:

-Original: “When we mixed DNA substrates containing unmethylated ori2 and crtS with RctB, we detected sporadic RctB-mediated contacts between ori2 and crtS (Supplementary Fig. 9b), suggesting the possibility of direct interactions between crtS and the 29/39m inhibitory sites in ori2.”

-Revised: “When we mixed DNA substrates containing unmethylated ori2 and crtS with RctB, we detected sporadic RctB-mediated contacts between ori2 and crtS (less than 1% of observed complexes) (Supplementary Fig. 12b), suggesting the possibility of interactions between RctB molecules bound to crtS and the ones bound to 29/39m inhibitory sites.”

13. Page 9 lines 9-10 The sentence requires correction.

We have corrected the sentence as follows:

- Original: “We followed the dynamics of both crtS and ori2 loci using three different DNA binding protein fluorescent proteins and their cognate sites inserted in the genome: ParBP1-CFP (parSP1 site near crtSori1), ParBMT1-yGFP (parSMT1 site near crtSwt), LacI-RFP (lacO array near ori2)

- Revised: “We tracked the dynamics of the two crtS sites and ori2 within the cell using three distinct fluorescent DNA-binding proteins, each specifically targeted to their binding sites in the genome: ParBP1-CFP bound to the parSP1 site near crtSori1, ParBMT1-yGFP bound to the parSMT1 site near crtSwt, and LacI-RFP bound to the lacO array near ori2”

14. Page 9 lines 21-22 The sentence requires correction. It is rather “the location (position) of the crtSon Chr1 affect on the time (timing) of replication initiation of Chr2....”

We have corrected the sentence as follows:

-Original: “First, we confirm that the location of crtS on Chr1 controls the time of initiation of Chr2 and thereby the copy number of Chr2, as in crtSwt it is 0.484 and in crtSori1 it is 0.601 (Fig. 4e, left and center) as observed before”

-Revised: “First, we confirmed that the position of crtS on Chr1 affects the timing of replication initiation of Chr2, and thereby influences the copy number of Chr2. In the strain with crtSwt, the Chr2 copy number is 0.484, while in the strain with crtSori1, it is 0.601 (Fig. 4e, left and center), consistent with previous observations”

15. Figure 1 legend …attTn7 site or LacZ ? …region containing RctB binding site…?

We appreciate the reviewer for pointing out this mistake. All RctB binding sites were inserted into the *lacZ* gene of the E. coli MG1655 chromosome. The figure legend has been corrected accordingly:

“pORI2 copy number (pORI2/oriC) in E. coli strains containing different RctB binding regions inserted into the lacZ locus. Each E. coli strain carries a plasmid (pORI2) that replicates using the Vibrio cholerae Chr2 origin (ori2). The ratio of pORI2 to the chromosomal origin (oriC) serves as a measure of ori2 replication activity. By inserting specific RctB binding regions into the genome, we can assess how these regions influence the replication of pORI2”

16. Figure 3 legend (DP) DP is not used anymore. Please delete.

We have removed the (DP) notation from the Figure 3 legend as suggested.

17. Figure 4 Representative images of cells containing the analyzed fluorescent signals could be added to better visualize and document the obtained data.

We thank the reviewer for this valuable suggestion. In response, we have included representative images of cells displaying the fluorescent signals at various stages of the replication cycle in Figure 4c,d. These images illustrate the observed duplication order for the different strains, enhancing the clarity of our microscopy results. To streamline the figure, we have moved the snapshot analysis to Supplementary Figure 14.

For the wild-type strain, the order of duplication is: ori1, followed by crtS, and then ori2.
For the strain containing an additional crtS site close to *ori1*, cells are born with two copies of ori2. The duplication sequence begins with ori1 (first crtS), followed by the duplication of the first ori2, then second crtS (original position), and finally the second ori2.

18. Figure 5 Why is OD decreased after SHX addition?

The decrease in OD following SHX addition is due to a reduction in cell size, a well-documented phenomenon in Vibrio cholerae under stringent response conditions, as described in previous studies (Kemter, Mol Microbiol., 2019, PMID: 30873684). This reflects the physiological changes induced by SHX treatment rather than cell death or lysis.

19. Figure 5 The Figure panels should be rearranged for better data presentation. Panels a and b could be moved to supplement. ChIP data is placed in one column (15, 30, 45 and 60 min) and on the side the MFA data (15, 30, 45 and 60 min).

We appreciate the reviewer’s suggestion to improve the clarity of Figure 5. In response, we have rearranged the panels as recommended. Panels a and b have been moved to the supplementary figures, and the ChIP data is now presented in a single column, with the corresponding MFA data aligned alongside for the 15, 30, 45, and 60-minute time points.

Additionally, we have enhanced the readability of the MFA by normalizing the data to the ter region of Chr1. In this revised version, non-replicating cells show a flat MFA at 1, while fully replicated cells display

the ori at 2 and the ter at 1. This adjustment should improve the clarity of replication progression throughout the time points, providing a more accurate and intuitive representation of the data. We modified fig5 legend accordingly.

20. Figure 5 What is the sample collection time? SHX incubation seems to be longer than 120 min. (as stated in the text in the figure legend and methods).

We thank the reviewer for pointing out this mistake. The correct SHX incubation period is 180 minutes, not 120 minutes as previously stated. We have revised the text in both the figure legend and methods section to reflect this correction.

Point-by-point response to the reviewers' comments

Reviewer #1 (Remarks to the Author):

The authors have done a great job in the revision. The added experiments, analyses, and edits greatly improved the manuscript. I have no further concerns.

We sincerely thank the reviewer for their kind comments on our revisions. We are grateful for the constructive suggestions, which significantly contributed to improving the quality of the manuscript.

Reviewer #2 (Remarks to the Author):

Thank you for analyzing the protein-DNA complexes in more detail.

We thank the reviewer for their valuable suggestions, particularly regarding the reanalysis of TEM data, which helped refine our approach and interpretation.

Have you considered only analyzing complexes in which the approximate diameter of the bound protein was in the range of 10 nm?

Thank you for this suggestion. We acknowledge the limitations of the TEM technique used in this study. To address this, we are currently investigating RctB binding on 39m sites using Cryo-EM, which we believe will provide more detailed and accurate insights into the structural properties of the complexes.

What evidence supports the idea that the large complexes presumed to arise by aggregation are functional?

At this stage, we do not have direct evidence that the large complexes formed by RctB aggregation are functional. However, we do observe that the DNA looping we describe is dependent on the 39mer sites, which occurs even in the absence of large aggregates. Interestingly, RctB shows a strong propensity to form aggregates, a characteristic also studied in the context of the initiator protein RepA by Rafael Giraldo's group (10.1038/srep25425). They have demonstrated that RepA can form amyloid structures *in vivo*, suggesting that this aggregation propensity may be conserved among initiator proteins. Further investigation will be needed to determine whether these large aggregates have a functional role for RctB.

Is it possible that DnaK and DnaJ remain bound to RctB to explain the observations?

We cannot rule out this possibility at this stage. Further studies will be required to assess whether DnaK and DnaJ contribute to the observed structures. Prior studies have shown that treatment with DnaKJ can alter RctB's behavior (10.1093/nar/gky790). For example, RctB treated with DnaKJ exhibits a higher band in EMSA experiments on 39mer, suggesting the formation of higher-order complexes. However, the exact mechanism of DnaKJ in this context remain unclear. Cryo-EM studies, which we are currently pursuing, should provide additional insights into these questions.

Page 6, line 22. Please delete “known to . . . in vivo.”

We modified the text accordingly: “To test this hypothesis, we performed a ChIP-seq of RctB in a mutant with the C21 > A mutation at the 29m site (29m*(C/A)) as this mutation has already been shown to reduce RctB binding in vivo”.

Page 16, line 20-21. The statement that the DNA was divided into 10 nm segments is not correct.

We modified the text accordingly: “For measurement, the DNA image was divided into segments of approximately 10 nm”.

Reviewer #3 (Remarks to the Author):

The revised version of the manuscript is improved. The title and the text were modified to better describe the experimental content. The discussion section was improved by incorporating broader evolutionary insights. The major improvement brings also the more extensive and more detailed TEM data analyses. Authors reorganized TEM data presentation. It is now presented in Figure 2, Figure S6 and Figure S12. The interaction of RctB with 39/29mer sites on Chr2 DNA fragments is now well documented. TEM experiments demonstrating nucleoprotein complex involving ctrS site, 39/29mer sites bridged by RctB is less convincing. The TEM images are essentially as in previous version. Authors point out that this complex is rarely observed. The application of crosslinking could possibly solve this problem in future investigations. This complex is an important element in the proposed model. Regardles of the RctB detection on ctrS site demonstrated in ChiPseq experiments it would be reasonably to consider transient dissociation of RctB from ctrS when replisome passes the site. All minor point were specifically addressed which improved and clarified text of the manuscript. The work is an important contribution to understanding of the regulatory mechanisms of DNA replication of Vibrio cholerae chromosomes. The proposed synchronization model assumes that the RctB inhibitory complex formed on crtS site and 39/29mer inhibitory site at Chr2 origin is remodeled when crtS is duplicated after fork progression on Chr1. The model is predominately supported by extensive ChIPseq analyses demonstrating RctB occupancy of 39/29mer sites on Chr2 during Chr1 fork progression.

We sincerely thank the reviewer for their thoughtful feedback and for recognizing the improvements in our manuscript. We appreciate the suggestion to apply crosslinking and will consider it in future investigations. Additionally, we are currently performing Cryo-EM studies, which we anticipate will provide further insights into the nucleoprotein complexes.
